# Anti-inflammatory effects of Chaishi Tuire Granules on influenza A treatment by mediating TRAF6/MAPK14 axis

**DOI:** 10.3389/fmed.2022.943681

**Published:** 2022-11-14

**Authors:** Lili Wang, Jiamei Guo, Yingying Wang, Pengcheng Zhao, Bin Liu, Yan Zhang, Yibai Xiong, Qing Chen, Lin Lin, Li Li, Xiaojuan He, Yong Tan, Mengmeng Cao, Jianfeng Yi, Tao Deng, Cheng Lu

**Affiliations:** ^1^Institute of Basic Research in Clinical Medicine, China Academy of Chinese Medical Sciences, Beijing, China; ^2^College of Chemical and Biological Engineering, Yichun University, Yichun, China; ^3^Institute of Microbiology, Chinese Academy of Sciences, Beijing, China; ^4^School of Life Science, Northwestern Polytechnical University, Xi'an, China; ^5^Institute of Pathogen Biology, NHC Key Laboratory of Systems Biology of Pathogens, Chinese Academy of Medical Sciences and Peking Union Medical College, Beijing, China; ^6^Research Center for Differentiation and Development of Traditional Chinese Medicine Basic Theory, Jiangxi University of Traditional Chinese Medicine, Nanchang, China

**Keywords:** Chaishi Tuire Granules, influenza A, anti-inflammation, TRAF6, MAPK14

## Abstract

**Objectives:**

Influenza is an infectious respiratory disease that can cause severe inflammatory reactions and threaten human life. Chaishi Tuire Granules (CSTRG), a Chinese patent medicine widely used clinically in the treatment of respiratory diseases in China, has a definite anti-inflammatory effect. However, the mechanism of CSTRG in the treatment of influenza is still unclear. This study aimed to demonstrate the anti-inflammatory effect of CSTRG on influenza A treatment and potential mechanisms.

**Methods:**

Influenza-associated mice pneumonia model was used to explore the antiviral and anti-inflammatory effects of CSTRG *in vivo*. Bioinformatics analysis methods such as network pharmacology and molecular docking were carried out to predict the main active components and potential anti-inflammatory targets of CSTRG. The anti-inflammatory activity of CSTRG was determined using the lipopolysaccharide (LPS)-induced macrophages RAW264.7 cells *in vitro*.

**Results:**

*In vivo* results showed that CSTRG can reduce the viral load in the lung tissue of infected mice, reduce the expression of TNF-α and IL-6 in lung tissue and serum, and regulate the host inflammatory response. Additionally, CSTRG treatment markedly improves the sick signs, weight loss, lung index, and lung pathological changes. Bioinformatics analysis predicted that six active compounds of CSTRG including quercetin, kaempferol, luteolin, beta-sitosterol, sitosterol, and stigmasterol could contribute to the anti-influenza activity through regulating the TRAF6/MAPK14 axis. The following research confirmed that CSTRG significantly inhibited pro-inflammatory cytokines (TNF-α and IL-6) by suppressing the expression of TRAF6 and MAPK14 in LPS-stimulated macrophages RAW264.7 cells.

**Conclusion:**

CSTRG might inhibit the inflammatory response by mediating the TRAF6/MAPK14 axis. In the future, in-depth research is still needed to verify the mechanism of CSTRG in the treatment of influenza.

## Introduction

The influenza viruses have a high incidence and are one of the most common respiratory viruses and mortality (1). And severe complications such as pneumonia and acute respiratory distress syndrome or ultimately death caused by severe influenza virus infection are often associated with cytokine storm characterized by hyper-induction of proinflammatory cytokine production (2). Several antiviral drugs, such as neuraminidase inhibitors, matrix-2 channel blockers, RNA polymerase inhibitors, and vaccines, have been developed to prevent and treat influenza virus infection. However, drug resistance and adverse effects seriously limit the efficacy of the available choices (3, 4).

With the characterizations of low toxicity, multi-components, multi-targets, and multi-pathway in the therapy of intricacy illness (5), Traditional Chinese Medicine has become a favorably and easy to accept therapy, and bioinformatics techniques could give rise to new drug research. Many herbs exhibit beneficial immunomodulatory effects and might be an effective therapy for influenza virus infection (2). Chaishi Tuire Granules (CSTRG) developed from a Chinese Herbal Formula and is mainly composed of ten herbs including Lonicera japonica Thunb, Rheum officinale, Bupleuri Radix, Isatis indigotica, Forsythia suspense (Thunb, Vahl), Artemisiae annuae herba, Rhizoma anemarrhenae, Dandelion, Scutellaria baicalensis Georgi, and Gypsum Fibrosum. CSTRG has the efficacy of clearing heat and detoxicating, and relieving superficies and clearing interior (6, 7), and it has been widely used to treat influenza virus infection for exceeding 20 years with obvious therapy effect and excellent safety (8). However, the mechanism of CSTRG in the treatment of influenza is still unclear.

**Figure 1:**
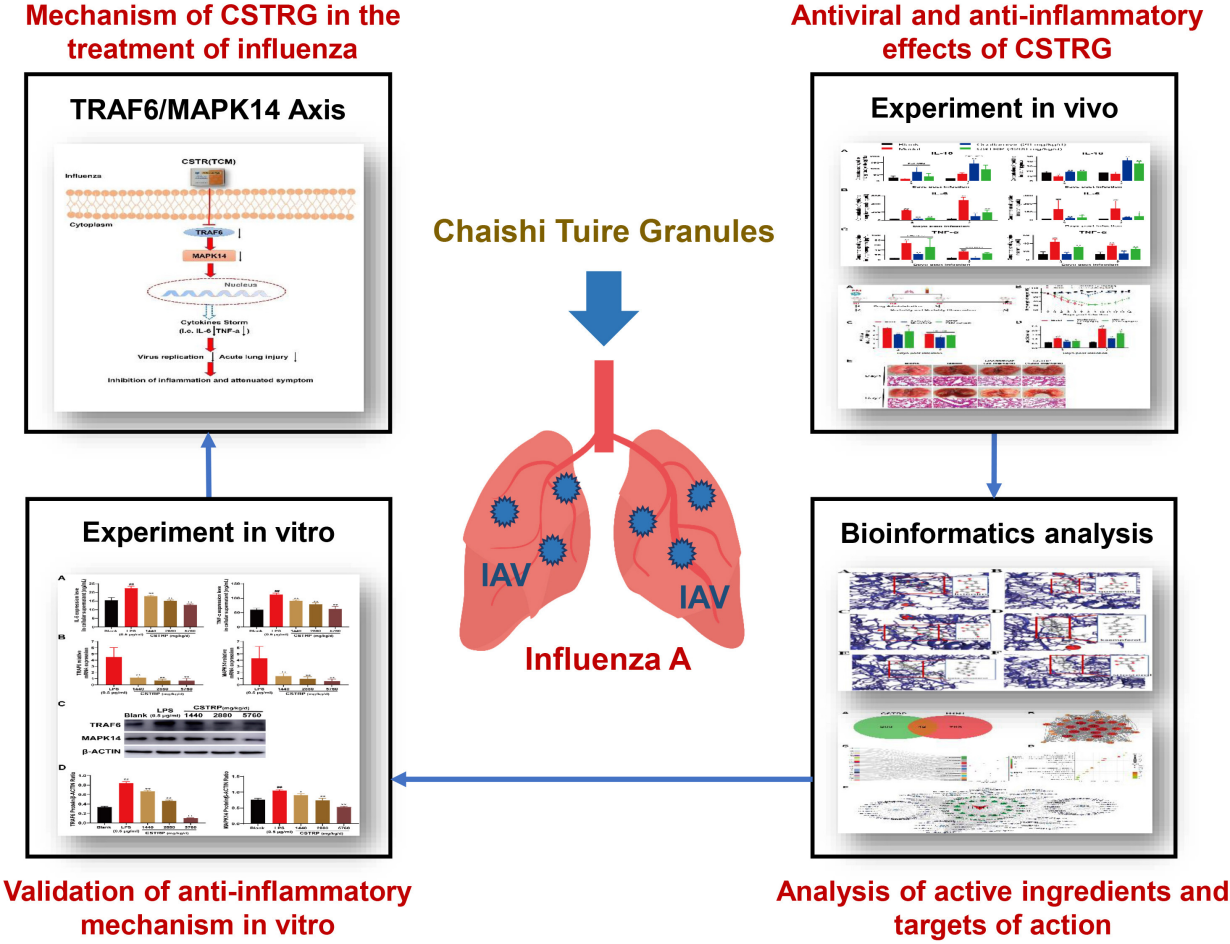
Graphical summary of this study.

In this article, the influenza-associated mice pneumonia model was used to study the antiviral and anti-inflammatory effects of CSTRG *in vivo*. Network pharmacology and molecular docking were carried out to predict the main active components and potential anti-inflammatory targets of CSTRG. We followed and verified the prediction with lipopolysaccharide (LPS)-induced macrophages RAW264.7 cells *in vitro* and demonstrate the anti-inflammatory effect of CSTRG on influenza A treatment and potential mechanisms (Figure 1).

## Materials and methods

### Reagents and drugs

CSTRG and oseltamivir phosphate were provided by Sinopsin Holding Guangdong Global Pharmaceutical Co., LTD, and Shanghai Roche Pharmaceutical Co., LTD. The CSTRG brown powder was dissolved in sterile water to 420 mg/ml (Z20010012, Lot no. 200301, China). Oseltamivir phosphate was soluble in PBS to 2 mg/mL (J20140121, M1066, China). The mouse IL-6 (Lot: EM004-96) and TNF-α (Lot: EM008-96) ELISA kits were obtained from Dakewe Biotech Co., Ltd. (Shenzhen, China). Recombinant Anti-TRAF6 antibody (ab33915) and Anti-p38 alpha/MAPK14 antibody (ab170099) rabbit polyclonal antibody were provided by Abcam, Inc. (Cambridge, Massachusetts, UK), β-actin (lot: AF0003) antibodies were purchased from Beyotime Biotechnology Co., Ltd. Multifactor detection Kit (Lot: L138965) was purchased from R&D SYSTEMS a biotech brand. CCK8 detection Kit (Lot: ZY121205) was purchased from Shanghai Zeyun Biotechnology Co., Ltd.

### Viruses and cells

The RAW264.7 macrophage cell line was obtained from the National Infrastructure of Cell Line Resource (China). Madin-Darby canine kidney (MDCK) cells were gained from the American Type Culture Collection (ATCC). Both RAW264.7 and MDCK were cultured in Dulbecco's modified Eagle's medium (Gibco, USA) supplemented with 10% fetal bovine serum (Gibco, USA) and 1% penicillin, and streptomycin at 37°C. Influenza A/Puerto Rico/8/34 strain (PR8) was preserved at our laboratory, which was propagated in 9–11 days old to maintain specific pathogen-free (SPF) embryona eggs, and the viral titers were determined by plaque assay.

### *In vivo* antiviral effects of CSTRG against influenzas virus

The animal experiments were approved by the Ethics Committee of the Institute of Microbiology, Chinese Academy of Sciences (202010002), and humane care for animals was compiled with the guidelines. SPF female BALB/c mice weighing 16–18 g purchased from Charles River (Beijing, China) were randomly divided into the following four groups (12 mice/group): Normal control group, Model group (PR8 + sterile water), Oseltamivir group (PR8 + Oseltamivir 20 mg/kg/d), and CSTRG group (PR8 + CSTRG 4,200 mg/kg/d); the daily dosage for mice was transmitted from the clinical dosage of an adult human (70 kg). Mice were mildly anesthetized with 3% isoflurane by intranasal infusion containing 6 Mouse Lethal Dose 50 (MLD_50_) PR8 virus suspension 20 μl, except for control mice. Then, the infected mice were orally administrated with CSTRG, Oseltamivir, or sterile water daily for successive 7 days. On day 4 and day 7 post-infection, six mice in each group (3 mice/day) were sacrificed to collect lungs and blood for viral titer, lung index, and lung pathological examination, as well as for inflammatory factor detection. And other 6 mice in each group were observed daily for 14 days to record the clinical signs, body weight change, and survival rate. A mice's body weight meets the criteria for euthanasia when it loses more than 30% of its body weight.

### Plaque assay

MDCK cells were seeded in 12-well tissue culture plates (2 × 10^5^ cells/well) and maintained at 37°C in a humidified atmosphere containing 5% CO_2_ for 24 h. The cells were inoculated with the supernatant of lung-homogenate. After adsorption at room temperature for 1 h, the inoculum was removed and the cells were overlaid with agarose (1%) medium containing 2.5 μg/ml TPCK-treated trypsin and then maintained for 3 days. The cells were then fixed with 4% paraformaldehyde and stained with 2% crystal violet solution.

### Lung index detection

The collected mouse lung tissue was cleaned using PBS buffer solution, dried with gauze, and then weighed. The lung index was calculated as follows: Lung index = lung weight/bodyweight × 100%.

### Histopathological staining

The lung tissue of mice was maintained by 4% paraformaldehyde, embedded in paraffin, and the section thickness reached 5 μm. The stained sections were observed and photographed under a 100× double-blind microscope with hematoxylin and eosin reagents. Based on the degree of lung injury, they were divided into four grades using the scoring criteria: no abnormal bronchioles and bronchial epithelium. Extravasation of bronchioles and bronchial lumen plasma cells; Inflammatory cells (mainly lymphocytes and neutrophils) in bronchioles, peribronchiolar and alveolar stroma; Collapse of alveoli or bronchus (atelectasis); and Diffuse or multifocal interstitial edema. No injury was 0, the mild injury was 1, the moderate injury was 2, and the severe injury was 3.

### Multi-factor detection

The IL-6 and TNF-α levels in mouse serum and lung homogenates were measured using an ELISA kit (R&D SYSTEMS, USA). The cytokine concentrations are calculated using the instruction manual using standard curves.

### Candidate compound screening

All chemical components in CSTRG were screened from the TCMSP database and wide-scale searches of the literature, including PubMed and China National Knowledge Infrastructure (CNKI) databases. Compounds that met the criteria of oral bioavailability (OB) ≥30% and drug similarity (DL) ≥0.14 were selected as candidate compounds for subsequent analysis.

### Potential targets fishing

Based on the GeneCards database, OMIM database and CNKI literature review and search for genes related to diseases caused by the H1N1 influenza virus, there were 810 genes related to the H1N1 influenza virus. The gene name was given as input into the functional protein association network database STRING to find out the target proteins related to the above genes, and construct the gene-target protein relationship library related to the H1N1 influenza virus. Almost every chemical component is associated with multiple targets or proteins, so regulating one target alone is difficult to effectively regulate the entire H1N1 influenza disease network. Regulating multiple targets at the same time is the key to searching for drugs and disturbing the influenza network. Using the Draw Venn Diagram tool (http://bioinfogp.cnb.csic.es/tools/venny/index.html), to obtain the target genes for the interaction between the CSTRG and influenza. Finally, 42 potential targets of CSTRG against H1N1 were screened, Venn diagram was drawn after intersecting and removing duplications.

### GO and KEGG enrichment analysis

Based on the DAVID database, GO function and KEGG pathway enrichment analysis were performed on 42 potential targets. According to the *p*-value < 0.05 condition, the GO and pathway of the analysis results were the next study.

### Protein interaction network

Input differentially expressed proteins using the String 11.0 tool (https://string-db.org/), species selection Homo Sapiens, a database of protein interactions, and the protein network diagram of differential proteins were obtained. The analysis results were then derived using Cytoscape 3.7.2 to plot the protein interaction network.

### Complex-target-disease (C-T-D) network composite diagram

The C-T-D network composite diagram was constructed using Cytoscape 3.7.2 software tool again. By analyzing network topology properties with the help of Network Analyzer, the network core node is analyzed by cytohubba plug-in, to roundly analyze the molecular mechanism of CSTRG against influenza A virus (IAV).

### Molecular docking simulation

The protein builds of the obtained compounds were derived from the PDB database; the 3D structure of the drug small molecule ligand was obtained from the PubChem database. Dockthor software was used for molecular docking, and the affinity-binding force value (kcal/mol) of each docking model was obtained. The binding energy of 5.0 kcal/mol was used as a screening criterion, then PyMOL and LigPlot+ 2.2.4 software were used to visually display the results.

### Preparation of drug-containing serum

Purchased female Sprague Dawley (SD) rats, weighing 250–300g, were randomly split into four groups (3 rats/group). CSTRG were orally administrated at 5,760 mg/kg/day, 2,880 mg/kg/day, and 1,440 mg/kg/day, respectively (9). 2 days after administration, the blood was collected and centrifuged at 3,000 RPM for 10 min to obtain the supernatant as serum. The water bath is heated for 30 min and filtered for sterilization and stored in refrigerator at −80°C for further use.

### CCK8 assay

Logarithmic growth RAW264.7 cells were digested with 0.25% trypsin (including EDTA), Inoculated with 1 × 10^4^/well in 96-well-plates, and incubated overnight in a 37°C, 5% CO_2_ incubator. The experiment set negative control holes, only 100 μl cell suspension was added into the negative control holes, blank holes were set at the same time, and the blank holes were added into the culture medium without cells. Each group was set with 6 multiple holes, after incubation for 24 h in the incubator, add 10 μl CCK-8 solution to each well, and the 96-well-plate was gently shaken and mixed. After incubation in the incubator for 2 h, the microplate reader was detected at 450 nm wavelength.

### *In vitro* anti-inflammatory effects of CSTRG

RAW264.7 cells were pretreated with different doses of serum-containing CSTRG for 1 h before the LPS (0.5 μg/ml) challenge, and the culture supernatants were collected 8-h post-incubation. And the mouse TNF-α (EM008-96) and IL-6 (EM004-96) ELISA kits were used to detect the secretion of inflammatory factors in cell supernatant.

### Real-time quantitative PCR

Total mRNA was obtained by TRIZOL reagent (Invitrogen, USA). RNA samples were reverted using the PrimeScript RT Master Mix kit (Aidlab Biotechnologies Co., Ltd., CHINA). GAPDH was selected as the internal control. The data were compared with Ct (2^−ΔΔ^ Ct). Primer 6.0 was used to design Primer sequences (Supplementary Table S1).

### Western blotting

Lysed cells can do this using the RIPA buffer; protein concentration was determined using a BCA protein assay kit (Beyotime Biotechnology, China). The protein was prepared in equal quantities (40 μg), separated by electrophoresis, and supported on 12% SDS-PAGE. Transferred to PVDF imprinting membrane. After 5% skim milk blockade, the specific primary antibody was incubated overnight at 4°C, and then the secondary antibody was used for incubation and internal reference with the aid of β-actin. The detection bands were visualized and analyzed using chemiluminescence imaging systems (FluorChem, ProteinSimple, San Jose, CA, USA) to obtain the results.

### Statistical analysis

All statistical analyses were conducted with Graphpad Prism 8.0 (Graphpad, San Diego, CA, USA) and data were presented as the mean ± standard deviation (S. D.). One-way ANOVA was used for multiple-group comparison. *p*-values of≤0.05 were considered statistically significant.

## Results

### CSTRG exhibited anti-inflammatory and therapeutic effect against IAV infection *in vivo*

#### CSTRG improved clinical signs and weight loss in mice caused by IAV infection

**Figure 2:**
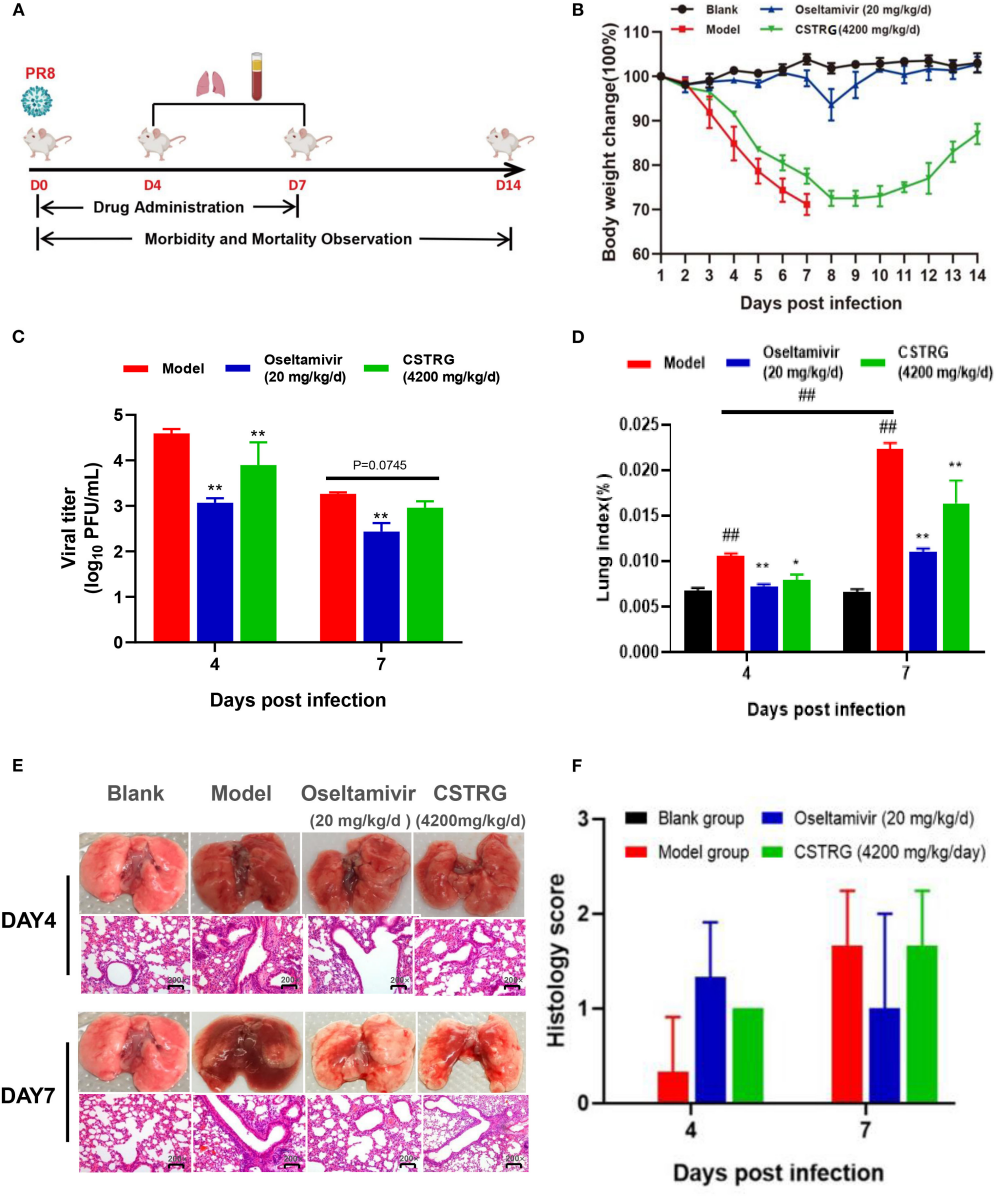
CSTRG shows anti-viral activity and alleviates lung injury caused by IAV infection in mice. **(A)** Female BALB/c mice were infected with 6 MLD_50_ of A/PR/8/34 (H1N1) virus and then were orally administered with CSTRG (4,200 mg/kg/d), Oseltamivir (20 mg/kg/d) for 7 successive days. Samples were collected on the 4th and 7th days post-infection (three mice/group/day). The clinical signs and weight change of infected mice were recorded for 14 successive days (six mice/group/day). **(B)** The body weight changes. **(C)** The influenza virus titers in mice lung tissues at the 4th and 7th days post-infection. **(D)** Lung index was recorded. **(E)** Sections of the lung tissues were visualized using hematoxylin and eosin (H&E) staining (Magnification: 200×). **(F)** Pathological score of lung tissue in mice. The data are presented as the means ± SD of 3 or 6 mice per group; compared with blank group, ^#^*p* < 0.05; ^##^*p* < 0.01; compared with model group, *p < 0.05; **p < 0.01.

To confirm the viral inhibitor properties of CSTRG *in vivo*, groups of mice are inoculated with A/PR/8 (H1N1) virus in 6 MLD_50_ and subsequently administered CSTRG, Oseltamivir, or placebo daily for 7 days, then we recorded the clinical signs and weight change for 14 days (Table 1 and Figures 2A,B). Results show that mice in the model group appeared obvious flu-like symptoms such as anorexia, huddling, ruffled fur, hunching, and lethargy. In contrast, CSTRG administration markedly improved the above illness symptoms.

**Table 1:** Assessment of morbidity in mice.

**Group**	**Dose**	**Clinical signs**				
		**Anorexia**	**Huddling**	**Ruffled fur**	**Hunching**	**Lethargy**
Control	/	-	-	-	-	-
Model	/	++++	++++	++++	++++	++++
Oseltamivir	20 mg/kg/d	++	++	+	+	+
CSTRG	4200 mg/kg/d	+++	++	++	+	++

-, None; +, Minimal; ++, Mild; +++, Moderate; ++++, Severe.

As for bodyweight changes, the weight of mice in the model group changed rapidly after infection with the virus, losing about 30% of their initial weight, and all mice died 7 days later. However, the weight loss of the mice treated with CSTRG was slightly improved and the bodyweight finally recovered to 90% of their initial body weight 14-day post-infection. These results indicated that CSTRG administration effectively improved clinical signs and weight loss in mice caused by IAV infection, although the protective effect was weaker than that of Oseltamivir administration.

#### CSTRG decreased virus load in mice lung tissues

To further identify the therapeutic effect of CSTRG on influenza, lungs were collected from infected mice at 4th and 7th days post-infection for detection of the virus titer. As shown in Figure 2C, the plaque assay results showed that Oseltamivir administration significantly reduced lung viral titers (>1 log) compared with placebo at 4th and 7th days post-infection. CSTRG treatment group also exhibited significantly decreased lung viral titers at 4-day post-infection, and presented reduced viral titers at 7-days post-infection, although the value was not significantly different from that of the placebo group. These results suggested that CSTRG had a certain effect on the treatment of IAV in mice.

#### CSTRG relieved lung injury caused by IAV infection

Lung index and histology were further examined to determine whether CSTRG administration alleviated lung injury induced by the IAV. Based on the observation of lung index changes in model group and control group mice, the model group was the highest as indicated by the results (Figure 2D). However, CSTRG treatment mice exhibited significantly reduced lung indexes compared with placebo at 4th and 7th days post-infection. The inhibition effect of Oseltamivir administration on lung index was slightly better than CSTRG. As shown in Figure 2E, histopathologic examination of lung tissue showed that the model group mice presented mild infiltration of monocytes and lymphocytes in alveolar space and thickened alveolar walls at 4 days post-infection, and moderate pathological damage was observed at day 7 post-infection. While Oseltamivir treatment only exhibited obvious lung pathology improvement on day 7 post-infection, at 4th and 7th days after infection, CSTRG was observed to improve lung pathological damage. Although the results of the pathological scores showed different trends, there were not statistically different (Figure 2F). These results indicated that CSTRG treatment improved lung injury caused by IAV infection.

#### CSTRG inhibited the pro-inflammatory response in the lungs and serum of mice infected with IAV

**Figure 3:**
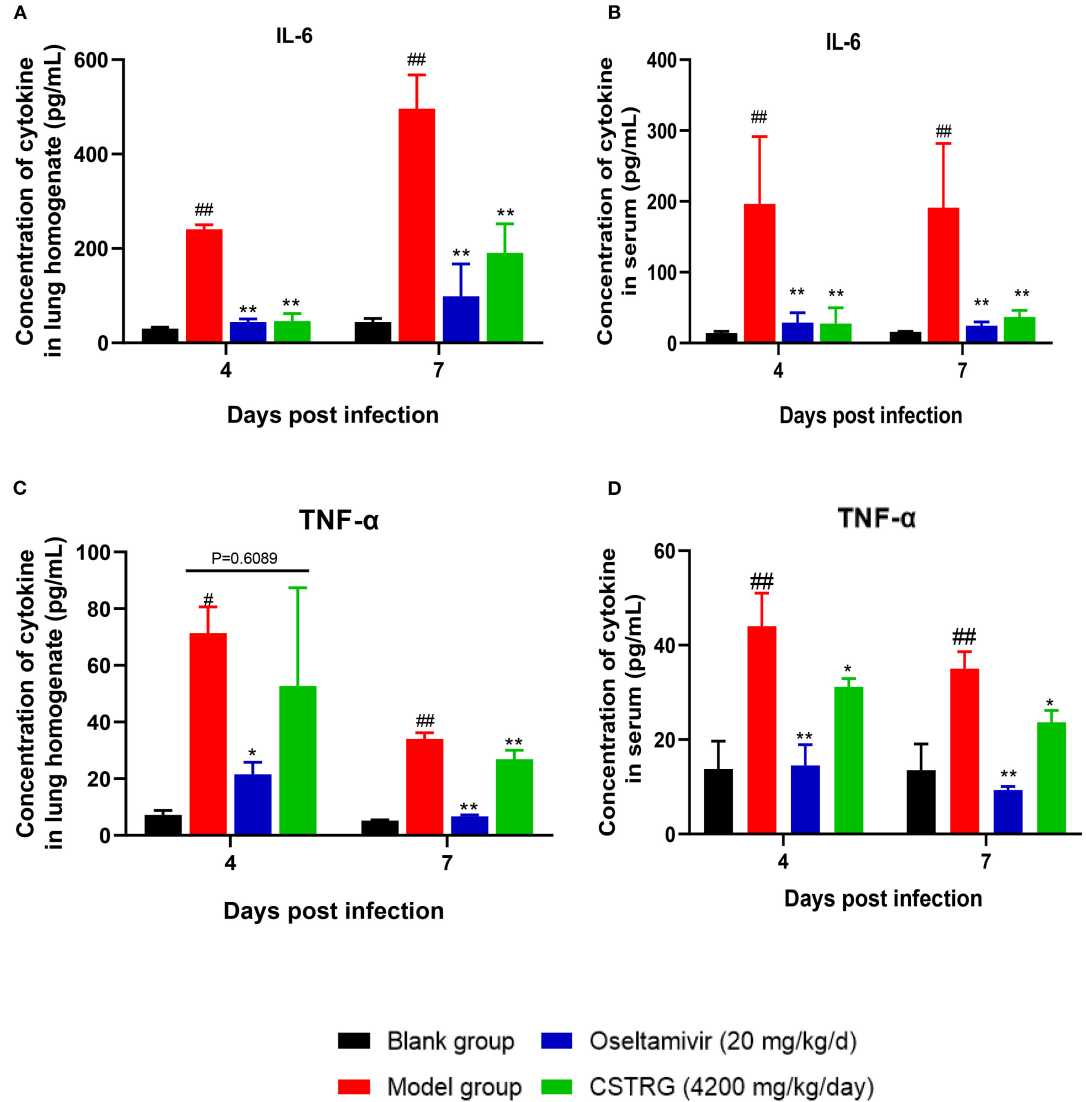
CSTRG shows anti-inflammatory activity against IAV infection in mice. **(A,B)** IL-6 secretion levels in lung tissues and serum. **(C,D)** TNF-α secretion levels in lung tissues and serum. The data are presented as the means ± standard deviations of the results (*n* = 3), compared with blank group, ^**#**^*p* < 0.05; ^**##**^*p* < 0.01; compared with model group, *p < 0.05; **p < 0.01.

To study the effect of CSTRG on the expression of inflammatory factors induced by IAV infection, we further tested the expression of IL-6 and TNF-α in the lungs and serum by ELISA. As shown in Figure 3, the levels of IL-6 and TNF-α in the model group were significantly increased on both day 4 and day 7 post-infection when compared with the control group. In contrast, similar to the positive control Oseltamivir, CSTRG treatment markedly suppressed the IL-6 and TNF-α expression compared with that of the model group. These results showed that CSTRG inhibited the inflammation in mice lungs and serum after IAV infection.

### Explore the potential active compounds and the anti-influenza mechanism of CSTRG through network pharmacology and bioinformatics analyses

#### Active ingredients screening

In total, 9 kinds of Chinese medicine compounds in the CSTRG formula were collected from the TCMSP database (not containing gypsum). All 168 candidate compounds were obtained by screening and passed with ADME (OB ≥ 30%, DL ≥ 0.14). The ultimate candidate compounds are shown in Supplementary Table S2.

#### Compounds in CSTRG active against influenza

**Figure 4:**
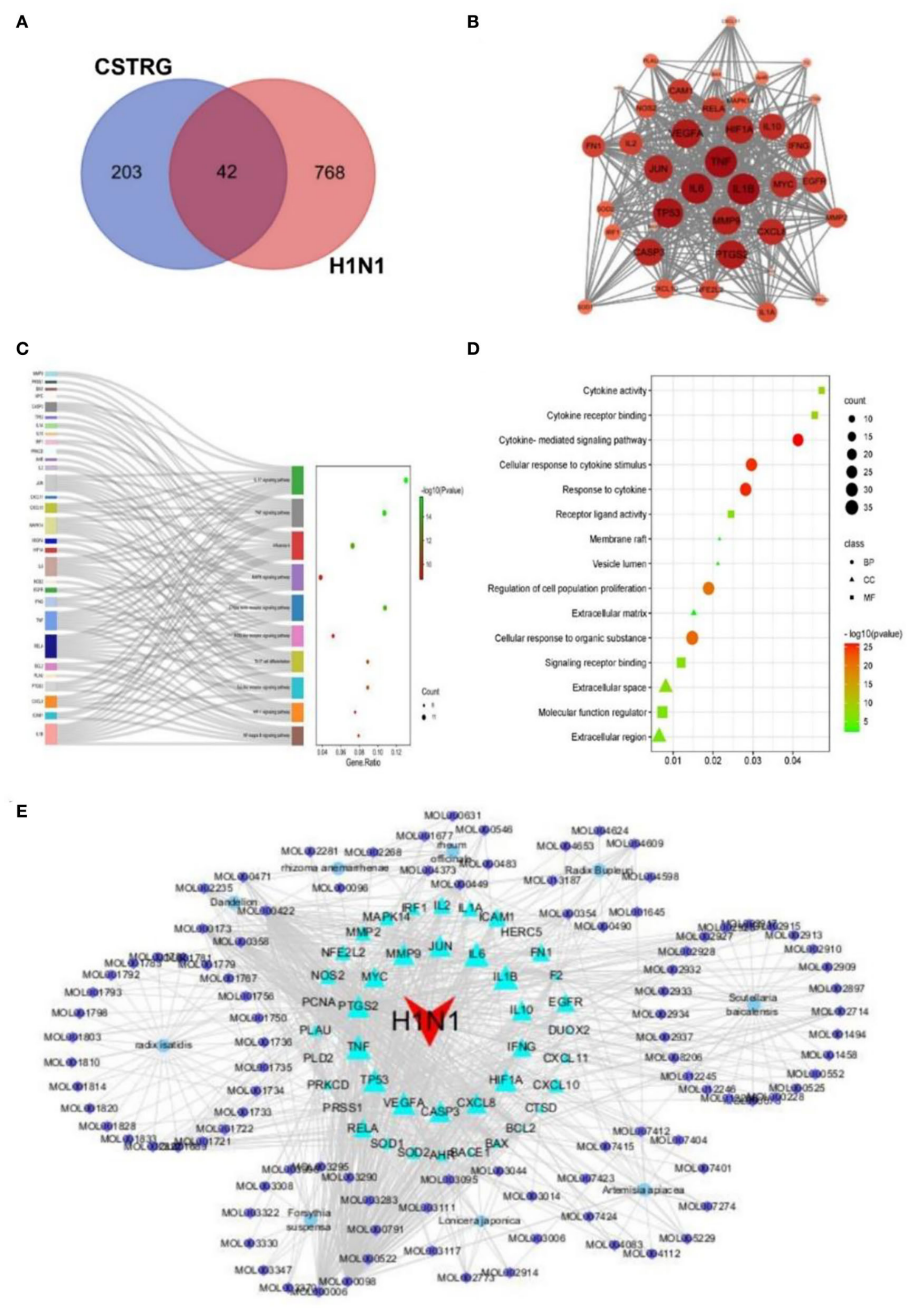
**(A)** 42 related intersections of drug (CSTRG) targets and influenza-related targets. **(B)** Protein-protein interaction (PPI) networks of CSTRG components with anti-influenza activity. The nodes represent related genes, and the strength of the data is supported when the circle is larger and the color is darker. **(C)** The functional enrichment analysis of CSTRG anti-influenza therapeutic targets was among the top 10 terms with the most significant enrichment in KEGG. **(D)** The five most significantly enriched BP, CC, and MF terms. **(E)** CSTRG component-target-disease network. The network was constructed to reveal interactions between active compounds and potential targets of CSTRG. Red triangular nodes represent the disease, cyan triangular nodes represent genes, blue round nodes represent Chinese medicines, and purple circular nodes represent active ingredients. The size of each target is positively related to its degree in the network.

All 810 targets directly and indirectly associated with influenza were obtained based on GENECARDS and OMIM databases (Supplementary Table S3). By employing the available TCMSP databases and wide-scale searches of the literature, including PubMed and CNKI databases, we obtained 245 CSTRG-related targets (Supplementary Table S4). Using the Draw Venn Diagram tool to obtain the overlapping targets, a total of 42 potential target gene-associated compounds were obtained, as shown in Figure 4A. Furthermore, detailed information on potential targets and active compounds in CSTRG has been given in Table 2.

**Table 2:** Information on 42 potential target gene-associated compounds.

**Uniprot ID**	**Protein name**	**Gene name**	**Degree**
P01584	Interleukin-1 beta	IL1B	72
P01375	Tumor necrosis factor	TNF	72
P05231	Interleukin-6	IL6	70
P04637	Cellular tumor antigen p53	TP53	68
P35354	Prostaglandin G/H synthase 2	PTGS2	66
P15692	Vascular endothelial growth factor-A	VEGFA	66
Q16665	Hypoxia-inducible factor 1-alpha	HIF1A	64
P05412	Transcription factor AP-1	JUN	64
P14780	Matrix metalloproteinase-9	MMP9	64
P42574	Caspase-3	CASP3	62
P10145	Interleukin-8	CXCL8	60
P22301	Interleukin-10	IL10	60
P01106	Myc proto-oncogene protein	MYC	60
P01579	Interferon gamma	IFNG	56
P00533	Epidermal growth factor receptor	EGFR	54
P05362	Intercellular adhesion molecule 1	ICAM1	54
Q04206	Transcription factor p65	RELA	54
P02751	Fibronectin	FN1	52
P60568	Interleukin-2	IL2	52
P01583	Interleukin-1 alpha	IL1A	46
P08253	72 kDa type IV collagenase	MMP2	46
Q16236	Nuclear factor erythroid 2-related factor 2	NFE2L2	46
P35228	Nitric oxide synthase, inducible	NOS2	46
P02778	C-X-C motif chemokine 10	CXCL10	44
Q16539	Mitogen-activated protein kinase 14	MAPK14	42
P10914	Interferon regulatory factor 1	IRF1	38
P04179	Superoxide dismutase [Mn], mitochondrial	SOD2	38
P00749	Urokinase-type plasminogen activator	PLAU	34
P00441	Superoxide dismutase [Cu-Zn]	SOD1	34
P35869	Aryl hydrocarbon receptor	AHR	32
Q05655	Protein kinase C delta type	PRKCD	28
Q07812	Apoptosis regulator BAX	BAX	26
O14625	C-X-C motif chemokine 11	CXCL11	26
P07339	Cathepsin D	CTSD	24
P00734	Prothrombin	F2	22
P56817	Beta-secretase 1	BACE1	18
P10415	Apoptosis regulator Bcl-2	BCL2	14
Q9NRD8	Dual oxidase 2	DUOX2	14
O14939	Phospholipase D2	PLD2	6
Q9UII4	E3 ISG15–protein ligase HERC5	HERC5	2
P12004	Proliferating cell nuclear antigen	PCNA	2
P07477	Trypsin-1	PRSS1	2

#### PPI network of target genes

The PPI network of target genes analysis showed that a total of 42 active ingredients acted on influenza-related target genes in CSTRG, and the PPI relationships among 42 CSTRG anti-influenza-related target genes were obtained using the STRING tool. The highly correlated and complex interleaving networks between targets can be shown by the results, which were the core targets of CSTRG in the treatment of H1N1 (Figure 4B). Among them, IL1B, TNF, IL6, TP53, PTGS2, VEGFA, HIF1A, JUN, MMP9, CASP3, CXCL8, IL10, MYC, IFNG, EGFR, ICAM1, RELA, FN1, IL2, IL1A, MMP2, NFE2L2, NOS2, CXCL10, and MAPK14 were considered to be hub genes.

#### KEGG and GO pathway enrichment analysis

According to KEGG analysis results, excluding irrelevant items, the top 10 pathways were found to be closely related to anti-inflammatory and antiviral influenza (*p* < 0.05). As shown in Figure 4C, KEGG enrichment analysis showed that the key targets were mainly concentrated in the IL-17 signaling pathway, TNF signaling pathway, C-type lectin receptor signaling pathway, influenza A, Toll-like receptor signaling pathway, and so on. As an important pathway of the inflammatory response, the TNF signaling pathway plays an important role (10). Among the core genes, IL-6 and TNF were most involved in the important gene core of the TNF signaling pathway. Moreover, IL-6 and TNF were inflammatory factors involved in the process of inflammatory response. The production of cytokines such as IL-6 is induced by TNF and is involved in inflammatory responses and oxidative stress processes (11, 12). The C-type lectin receptor signaling pathway plays an important role in host fungal infection by coordinating the immune system against infection (13).

Previous studies have shown that innate immune signaling through TLR4-TRIF-TRAF6 is a key genetic pathway that determines the susceptibility to acute lung failure *in vivo* (14), and plays an important role in initiating the innate immune response (15), and influenza A. GO enrichment analysis was performed for 42 candidate targets, a total of 972 biological processes (BP) terms, 13 cellular components (CC) terms, and 48 molecule function (MF) terms were obtained by GO analysis, from which the top 5 terms were selected (*p* < 0.05), as shown in Figure 4D. For BP, the targets mainly participated in the regulation of proliferation, apoptosis, and response to cytokine. For MF, the targets were mainly responsible for the activity of cytokine activity, as well as the binding of protein, cytokine receptor, and transcription factor. For CC, the targets were mainly distributed in vesicle lumen, extracellular space, and matrix. GO results show that immune and inflammatory responses play an important role. These results suggest that the symptoms and pathogenesis of lethal H1N1 virus infection are primarily caused by an excessive inflammatory response (16).

#### C-T-D network analysis

Through the construction and analysis of a C-T-D network, the multi-component, multi-target effect of CSTRG in the treatment of influenza was also demonstrated, and the mechanism of action was also discovered. This network reflects the complex relationships between active compounds and their potential targets (17). In the network, the principle of molecular docking ligand selection was carried out according to the degree value, and the top six compounds were obtained, the number of connections between molecules and targets in the network core structure is represented by the degree value of molecules (18). The more important the components of CSTRG are screened, the greater the degree value. As shown in Figure 4E, some compounds can take effect on multiple targets concurrently; meanwhile, multiple targets can be taken effect on by multiple compounds concurrently. This consequence reveals the multi-component and multi-target traits of TCMs. Some compounds, for instance MOL000098 (quercetin), MOL000006 (luteolin), MOL000422 (kaempferol), MOL000358 (beta-sitosterol), and MOL000449 (Stigmasterol), have been lectured to restrain activity against the influenza virus (19–22).

#### Acquisition of major active compounds and molecular docking

**Figure 5:**
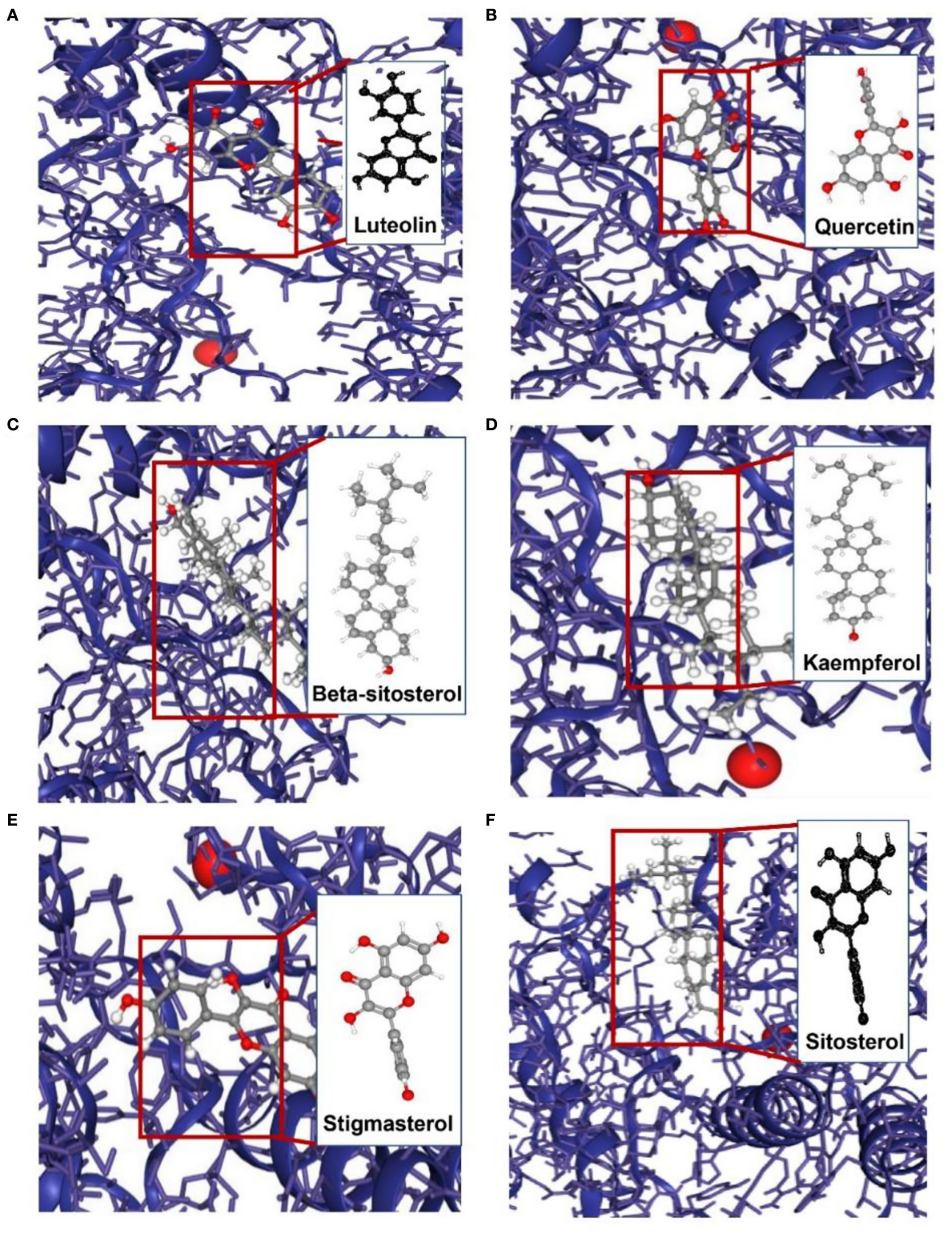
Molecular models of the binding of MAPK14 with **(A)** luteolin, **(B)** quercetin, **(C)** beta-sitosterol, **(D)** kaempferol, **(E)** stigmasterol and **(F)** sitosterol.

To appraise the binding potential of the significant bioactive compounds of the CSTRG with the major target proteins, the first was a search for the active ingredients of CSTRG formulations obtained from TCMSP and OMIM databases. The screening principle is that the active compounds in CSTRG are common to the lowest three TCMs. Meanwhile, to determine the frequencies of these ingredients and their mechanism of action, this screening method resulted in 6 compounds that are possibly responsible for the influenza virus properties of the TCMs. These active ingredients are quercetin, kaempferol, luteolin, beta-sitosterol, sitosterol, and stigmasterol. These compounds may underpin the anti-inflammatory and antiviral activity of these TCMs, but the exact mechanism is unknown. While each viral enzyme of the influenza virus is a potential drug target, the key target protein MAPK14 which is closely related to influenza A was screened from the core genes by referring to the literature (23, 24), MAPK14 was selected to be the focus of our further analyses. MAPK14 is essential in mediating viral sensor signaling cascades that regulate the expression of antiviral chemokines and cytokines (25). An extracellular signal-regulated kinase (ERK), P38 MAPK, and C-Jun N-terminal kinase are members of the mitogen-activated protein kinase (MAPKs) family, which play an important role in the expression of pro-inflammatory cytokines (26). Studies have shown that the activation of the MAPK14 protein is a key to elevated circulating pro-inflammatory cytokine levels in patients with severe influenza (3, 4). And participated in several steps of the IAV infection cycle. Binding energy ≤-5.0 kcal/mol was selected as the screening criteria. Molecular docking results showed that all the compounds were lower than −5 kcal/mol (Table 3). These important core compounds have a certain affinity to the protein crystal structure of MAPK14 (Figure 5). Hence, these core compounds have a good binding ability with MAPK14 protein, and the conformation of conjugated compounds is stable, which indicates that core compounds are indeed the key components of the TCMs. Of all the six compounds, the corresponding drug active ingredients that could interact with MAPK14 pro are the phenomenon of high binding, low binding energy, and conformation stability.

**Table 3:** Docking model of the common active ingredients of CSTRG with MAPK14 protein.

**Mol ID**	**Compound**	**Formula**	**MW (g/mol)**	**OB (%)**	**DL index**	**3CLpro (kcal/mol)**
MOL000006	Luteolin	C_15_H_10_O_6_	286.250	36.163	0.246	−7.903
MOL000098	Quercetin	C_15_H_10_O_7_	302.250	46.433	0.275	−8.037
MOL000358	Beta-sitosterol	C_30_H_52_O	414.790	36.914	0.751	−9.376
MOL000359	Sitosterol	C_29_H_50_O	414.790	36.914	0.751	−9.376
MOL000422	Kaempferol	C_15_H_10_O_6_	286.250	41.882	0.241	−7.474
MOL000449	Stigmasterol	C_29_H_48_O	412.770	43.830	0.757	−9.573

### CSTRG exhibits anti-inflammatory activity by mediating TRAF6/MAPK14 axis *in vitro*

As the network analysis and molecular docking results showed that the TRAF6/MAPK14 axis might play the key pathogenic role in the treatment of influenza by CSTRG, we further examined the anti-inflammatory activity of CSTRG using LPS-stimulated RAW264.7 macrophages *in vitro* model, in which the stimulation of LPS can cause RAW264.7 differentiated into spindle-shaped M1-like macrophages accompanied by upregulation of TNF-α and followed by the release of inflammatory factors (27).

#### CSTRG showed no significant cytotoxicity in RAW264.7 cells

We performed the CCK8 assay firstly to determine the cell viability after CSTRG drug-containing serum treatment in RAW264.7 cells. The results showed that even the 10% CSTRG (5,760 mg/kg/day) drug-containing serum gave no cytotoxicity in RAW264.7 cells (Supplementary Figure S1). In addition, we further explored the release of LPS-induced TNF-α at different concentrations (0, 0.5, and 1 μg/ml) and at different times (8, 12, 24, and 48 h) to find out the suitable acting condition (Supplementary Figure S2). Finally, 0.5 μg/ml LPS treatment for 8 h was chosen for the following experiments (28).

#### CSTRG inhibited the differentiation of RAW264.7 cells

To investigate the anti-inflammatory activity of CSTRG, RAW264.7 cells were pre-treated with different doses of CSTRG drug-containing serum for 1 h before the LPS challenge. After incubating for 8 h, we first examined the morphological changes of RAW264.7 cells under optical microscopy. Compared with round-shaped control cells, LPS stimulated cells differentiated into spindle-shaped macrophages with elongated pseudopodia. While following CSTRG drug-containing serum treatment, the cell morphology changes were ameliorated (Data were not shown).

#### CSTRG inhibited secretion of pro-inflammatory cytokines in LPS-induced RAW264.7 cells

**Figure 6:**
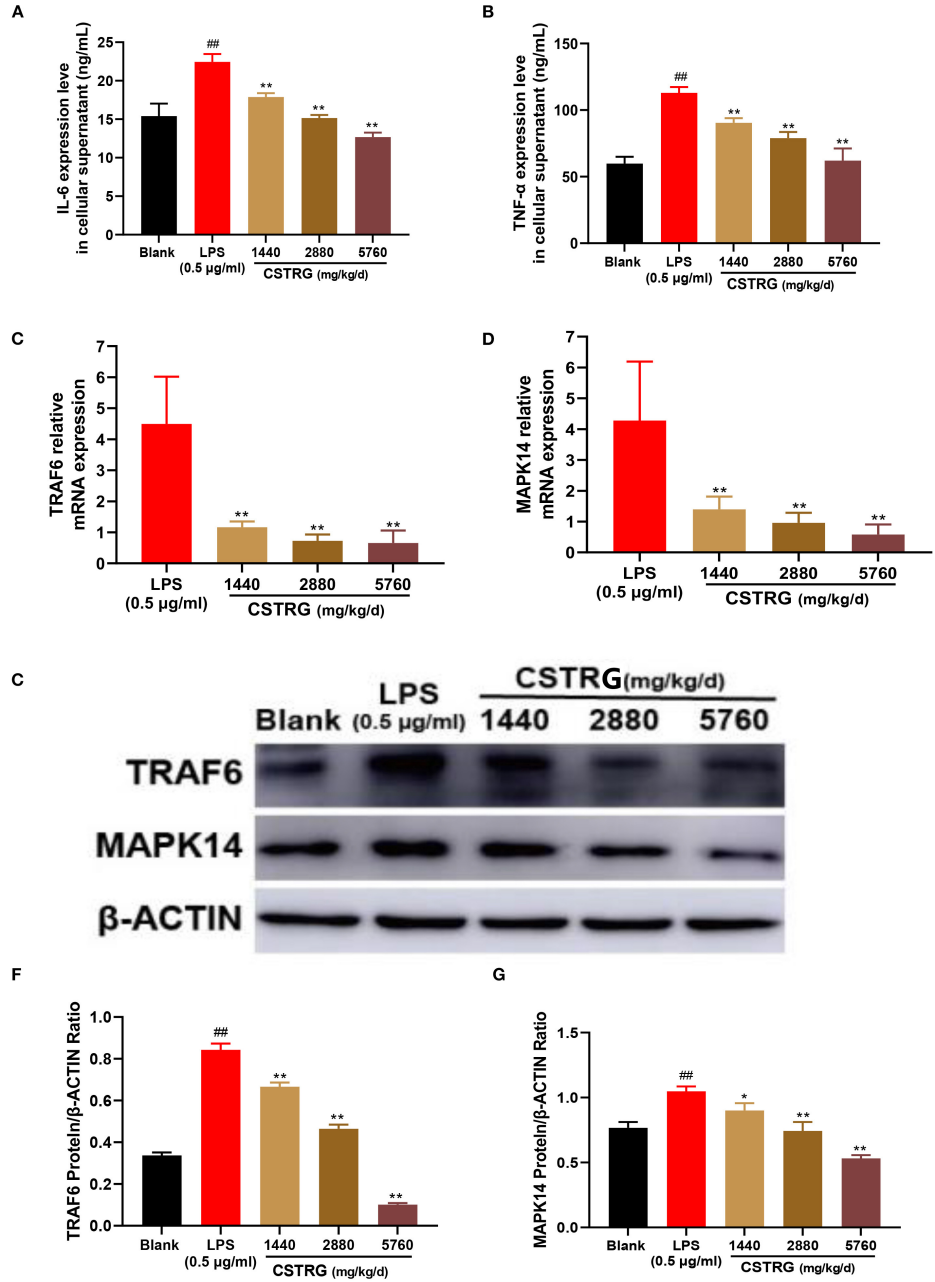
CSTRG exhibits anti-inflammatory activity effects in LPS-stimulated RAW264.7 cells by attenuating the TRAF6/MAPK14 axis. **(A,B)** The levels of TNF-α and IL-6 in the culture supernatants were detected by ELISA kits. **(C,D)** The mRNA levels of TRAF6 and MAPK14 were determined by qPCR analysis. **(E–G)** The protein levels of TRAF6 and MAPK14 were determined by western blot analysis. The data are presented as the means ± standard deviations of the results from three independent experiments. Compared with blank group: ^**#**^*p* < 0.05; ^**##**^*p* < 0.01; compared with model group; *p < 0.05; **p < 0.01.

We further detected the secretion levels of TNF-α and IL-6 in the supernatants and compared them between CSTRG drug-containing serum treated and mock-treated RAW264.7 cells. The results showed that the elevated expressions of TNF-α and IL-6 were significantly inhibited by CSTRG drug-containing serum treatment in a dose-dependent manner (Figures 6A,B). The anti-inflammatory effect of CSTRG on the secretion of pro-inflammatory cytokines IL-6 and TNF-α in LPS-induced RAW264.7 macrophages was demonstrated by the results.

#### CSTRG demonstrates anti-inflammatory activity against LPS-induced pro-inflammatory responses by mediating TRAF6/MAPK14 axis

To study whether the inhibition effect on LPS-induced pro-inflammatory cytokines production by CSTRG was related to the inhibitory of the mediating TRAF6/MAPK14 axis, we further detected the mRNA and protein levels of TRAF6 and MAPK14. The results showed that CSTRG drug-containing serum treatment significantly reduced the expression of TRAF6 and MAPK14 in a dose-dependent manner, both in mRNA and protein levels (Figures 6C–G). These results indicated that CSTRG exhibited potent anti-inflammatory activity against LPS-induced pro-inflammatory by attenuating the TRAF6/MAPK14 axis.

## Discussion

**Figure 7:**
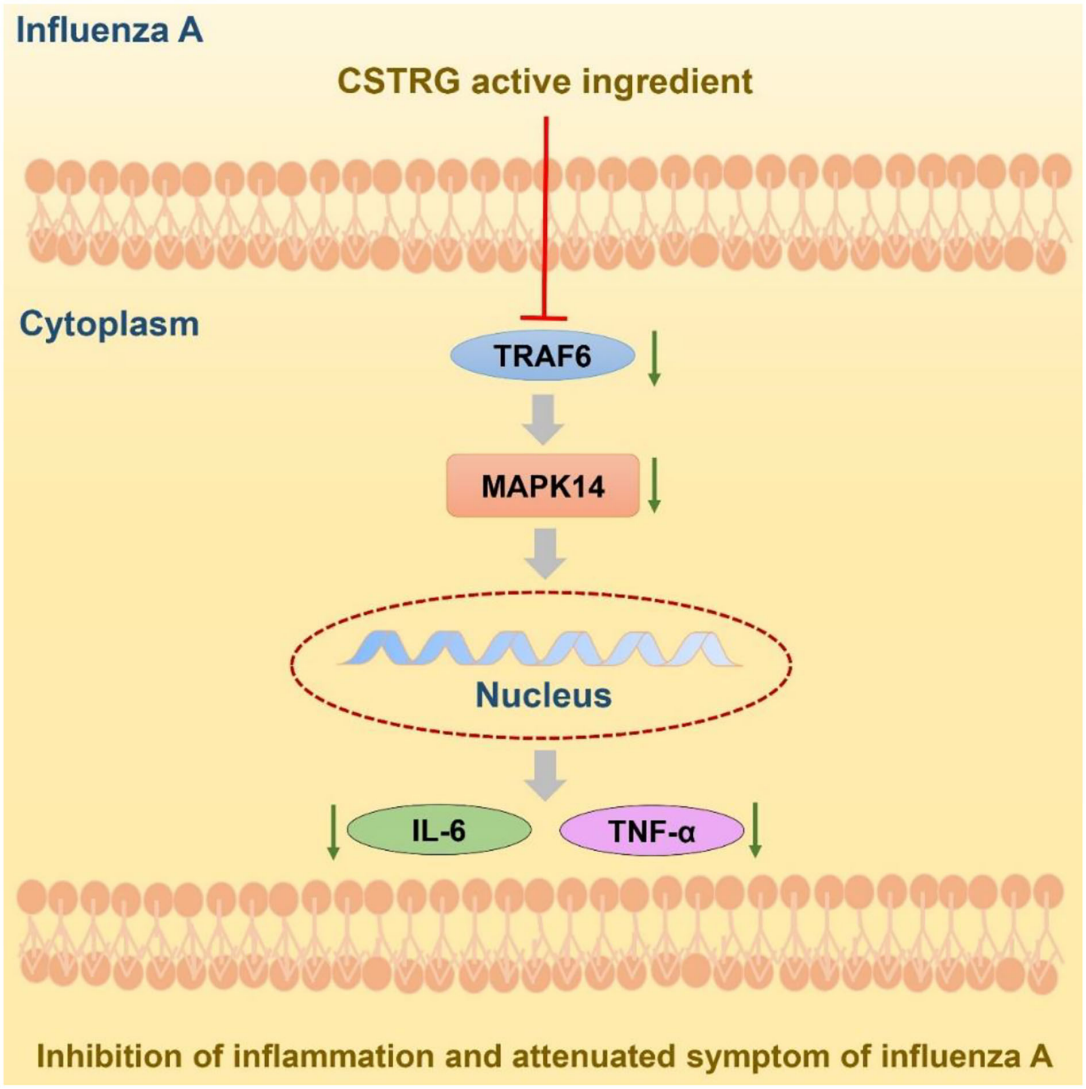
Potential model of CSTRG treating influenza virus infection through suppressing both virus replication and viral-induced cytokine storms via regulating the TRAF6/MAPK14 axis.

Given the serious threat caused by the influenza virus and the limitation of the available therapy, finding new drugs to treat influenza virus infection is still very essential (29). With the characterizations of multi-components, multi-targets, and multi-pathway in the treatment of complex diseases, Traditional Chinese Medicine has become a popular and acceptable therapy (30). Developed from a Chinese Herbal Formula, CSTRG has been widely used for treating influenza virus infection and showed obvious therapeutic effects. This study first showed that the CSTRG was able to play an anti-inflammatory effect, and it had a significant inhibitory effect on the inflammatory process. Improving the immune function of the host body, both virus replication and virus infection have inhibitory effects. The experimental studies have shown that CSTRG exerts its anti-influenza effects by reducing lung index and viral titers, improving lung damage, and reducing the secretion of proinflammatory cytokines in lung tissue, improve the immune function of the host (Figure 7).

In recent years, there has been a growing study of the efficacy of traditional Chinese medicine. Using network analysis to greatly increase the understanding of the various mechanisms of drugs (31). The wide application of bioinformatics has played an important role in promoting the development of analytical TCM. Traditional Chinese medical network patterns may lead to new poly compound discoveries (32). Molecular docking is a significant pathway of structural molecular biology and computer-aided drug design in medicines (33). A series of bioinformatics analyses such as network pharmacology and molecular docking were firstly carried out to predict the main active compounds of CSTRG and the potential anti-influenza targets and signaling pathways.

The results indicated that the core active compounds of CSTRG including quercetin, kaempferol, luteolin, beta-sitosterol, sitosterol, and stigmasterol may contribute to the anti-inflammatory activity via mediating TRAF6/MAPK14 axis. Among them, based on bioassay results, quercetin, kaempferol, luteolin, beta-sitosterol, and sitosterol are the important active components of CSTRG compounds. These compounds have been reported in many Chinese herbal anti-inflammatory and anti-influenza network pharmacology studies. Meanwhile, the same active ingredients are included in the multi-flavor monotherapy of CSTRG, in which quercetin is an important active ingredient in forsythia, artemisia annua, dandelion, honeysuckle, and other plants. Luteolin is an important active component of honeysuckle, forsythia, and artemisia annua. Sitosterol is an important active component of scutellaria baicalensis, honeysuckle, rhubarb, radix isatidis, and forsythia suspensa. Beta -sitosterol was found in the herbal medicines mainly from scutellaria baicalensis, radix isatidis, and artemisia annua. Kaempferol is an important active ingredient in honeysuckle, forsythia, bupleurum, artemisia annua, and anemone anemae. Isatis indigotica (34), whose dried roots are named ban lan gen, has a long history of clinical use in the treatment of viral infection, tumor, inflammation, and can inhibit the pro-inflammatory effect induced by IAV. In 2007, it was reported for the first time that the compound extract of scutellaria baicalensis could significantly inhibit the propagation of influenza virus and induce IFN-γ expression *in vivo* (35). Umbelliferae is an important traditional Chinese medicine in the treatment of hepatitis, nephritis, inflammation and bacterial and viral infections (36). Moreover, the combined application of radix Bupleurum and scutellaria baicalensis has double inhibitory effect on the synthesis and release of inflammatory factors, and also has systemic effect on inflammatory pain. Combined application of radix bupleuri and scutellaria baicalensis is the most common treatment for inflammatory diseases (37). Rhubarb is one of the important traditional Chinese medicines in the treatment of microorganisms, inflammation, fever and viral infection, and emodin is a well-known antiviral compound with broad spectrum of efficacy (38). Single drug treatment or multi-drug combination in CSTRG compound has anti-inflammatory effect and regulation of host immune response, prevention of influenza. Quercetin, one of the most taken flavonoids in diet, was reported to be effective in the treatment of influenza virus infection via improving the virus-induced pulmonary endothelial barrier dysfunction (39). Quercetin has been proved to be effective anti-inflammatory (40), Li et al. (41) conducted experiments in different animal models and found that quercetin inhibited the quercetin has anti-inflammatory activity and inhibits LPS-induced TNF-α and IL-8 production in macrophages and lung A549 cells. Luteolin is a common flavonoid of medicinal plants and has been proved to have strong anti-inflammatory activity *in vitro* and *in vivo*. Aziz et al. (42). the main pharmacological mechanism of luteolin is the regulation of transcription factors STAT3, NF-κB, and AP-1 to exert anti-inflammatory activity. Guo et al. (43). To investigate the role of luteolin in the prevention of TLR3-mediated corneal viral infection-related inflammatory responses and promoting the expression of inflammatory factors in human corneal fibroblasts (HCFs). Beta-sitosterol, sitosterol, and stigmasterol belong to plant sterols with good anti-bacterial and anti-inflammatory effects (44). What's more, these sterols have a similar structure to cholesterol, which may impact influenza virus entry (45). Kaempferol is a natural flavonoid compound that, due to its anti-inflammatory properties, is used to treat many diseases caused by acute and chronic inflammation and has been proven to be a safe and effective natural dietary anti-inflammatory agent both *in vivo* and *in vitro* (46). Natural products exist in different phytochemical compositions. These compounds have excellent anti-inflammatory properties both *in vitro* and *in vivo*.

Considering the TRAF6/MAPK14 axis play a significant role in inflammation, which helps the host to defend against influenza virus infection (47), we further confirmed the anti-inflammatory activity of CSTRG with LPS-induced macrophages RAW264.7 cells *in vitro*. Given the active compounds of CSTRG had a strong affinity for MAPK14, which can be activated by IL-17/Act1/TRAF6 modification and lead to the upregulation of IL-6 and TNF-α, we speculated that CSTRG might modulate the expression of these factors and then the hypothesis was confirmed. Therefore, CSTRG exhibited anti-inflammatory activity *in vitro* via suppressing the expression of TRAF6 and MAPK14 and then the release of TNF-α and IL-6.

Complications such as pneumonia and acute respiratory distress syndrome or ultimately death caused by severe influenza virus infection are often associated with cytokine storm characterized by hyper induction of proinflammatory cytokine production (2). CSTRG showed anti-inflammatory effects *in vitro* and *in vivo*. It not only inhibitory effect on LPS-stimulated pro-inflammatory *in vitro*, but also inhibited the expression of inflammatory factors *in vivo*, alleviated influenza-associated pneumonia, it was benefited from the immunomodulatory effects, and finally improved the survival state of infected mice. Further studies are needed to determine whether CSTRG inhibition of virus replication is directly caused by antiviral activity.

CSTRG has been widely used for influenza treatment in China with obvious therapeutic effects and an excellent safety profile. This study proved that CSTRG can alleviate influenza-induced lung lesions with both anti-inflammatory and immunomodulatory activity *in vivo*, which provides evidence for its clinical application. Although the anti-influenza effect of CSTRG cannot compare with that of Oseltamivir in individual administration, we speculated that CSTRG may have a potent anti-influenza effect when combined with Oseltamivir (48). In addition, the change of cytokine profiles indicated that CSTRG might have a potential effect on the inhibition of cytokine storm induced by epidemic encephalitis B virus (6).

## Conclusions

CSTRG not only showed anti-inflammatory activity *in vitro* by downregulating the TRAF6/MAPK14 axis but also has a significant protective effect against IAV *in vivo*. Further studies showed that the *in vivo* anti-influenza effect of CSTRG may be related to inhibiting the secretion of inflammatory cytokines of the host body, and had a good protective effect on IAV infection. It provides the clinical basis for CSTRG treatment of IAV infection. In the future, in-depth research is still needed to verify the mechanism of CSTRG in the treatment of influenza.

## Data availability statement

The original contributions presented in the study are included in the article/Supplementary material, further inquiries can be directed to the corresponding author/s.

## Ethics statement

The animal experiments were approved by the Ethics Committee of the Institute of Microbiology, Chinese Academy of Sciences (202010002).

## Author contributions

LW, JG, and YW conceived and designed this study. LW, JG, YW, and PZ performed the data extraction, analysis, interpretation, and wrote the initial draft. BL, YZ, YX, QC, and LLin were responsible for literature checking and data entry. CL, TD, JY, XH, YT, MC, and LLi assisted with data interpretation. LW and JG contributed equally to this work. All authors contributed to the final manuscript.

## Funding

This work was supported by the Innovation Team and Talents Cultivation Program of the National Administration of Traditional Chinese Medicine (ZYYCXTD-D-202005); the National Key R&D Program of China (2019YFC1712003); the Fundamental Research Funds for the Central public welfare research institutes (Z0653). National Key R&D Program of China (2020YFE0205100). An intramural Startup Funds from Institute of Microbiology, Chinese Academy of Sciences to T.D (Y954018S). The National Natural Science Foundation of China (31601151).

## Conflict of interest

The authors declare that the research was conducted in the absence of any commercial or financial relationships that could be construed as a potential conflict of interest.

## Publisher's note

All claims expressed in this article are solely those of the authors and do not necessarily represent those of their affiliated organizations, or those of the publisher, the editors and the reviewers. Any product that may be evaluated in this article, or claim that may be made by its manufacturer, is not guaranteed or endorsed by the publisher.
