# Supplementary material for: Anti-inflammatory effects of Chaishi Tuire Granules on influenza A treatment by mediating TRAF6/MAPK14 axis
Source: Front Med (Lausanne). 2022 Nov 14;9:943681. doi: 10.3389/fmed.2022.943681 (PMC9701735; doi:10.3389/fmed.2022.943681)

Chaishi-Tuire Particle (CSTRP) shows anti-viral activity and alleviates lung injury caused by influenza A virus infection in mice. Female BALB/c mice were infected with 6 MLD50 of A/PR/8/34 (H1N1) virus and then were orally administered with CSTRP (4200 mg/kg/d), Oseltamivir (20 mg/kg/d) for 7 successive days. Samples were collected at the 4th and 7th days post infection (3 mice/group/day).

DAY4: Pulmonary appearance and histopathologic examination of lung tissue

Blank

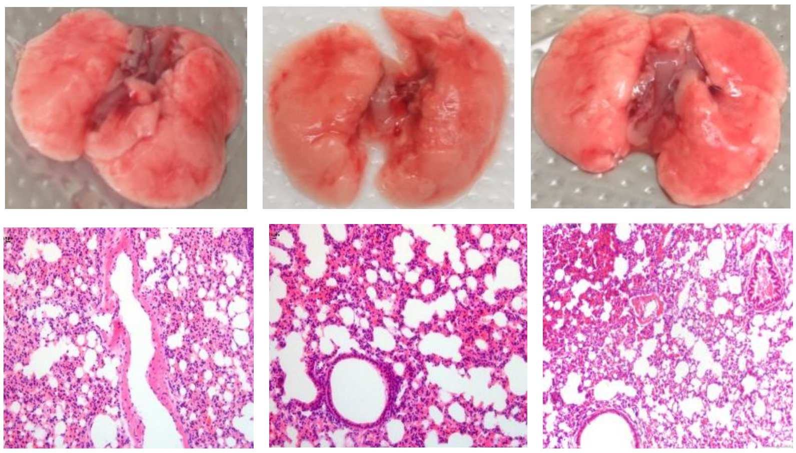

Model

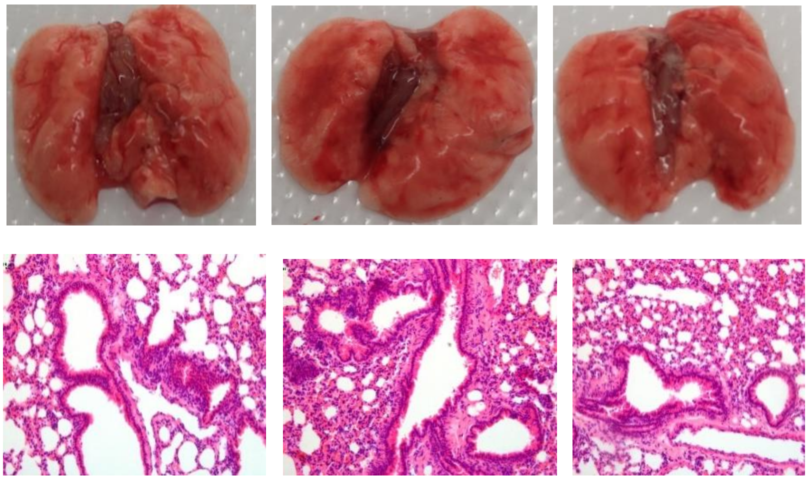

Oseltamivir (20 mg/kg/d)

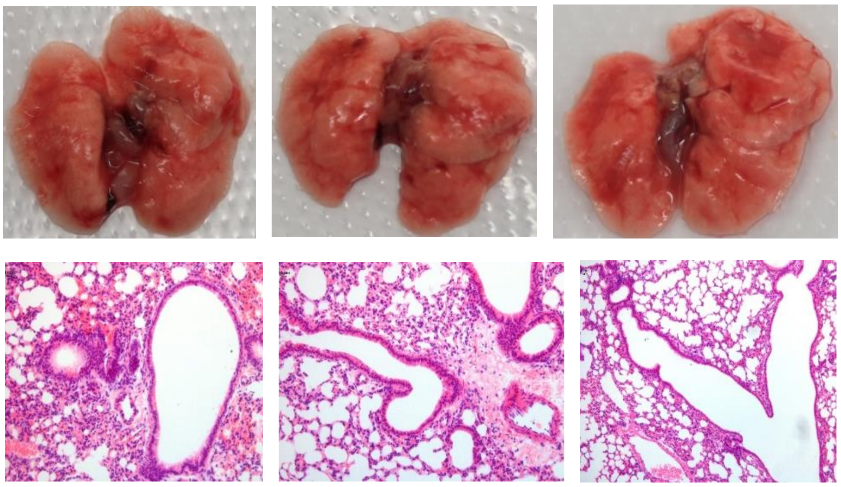

CSTRP (4200 mg/kg/d)

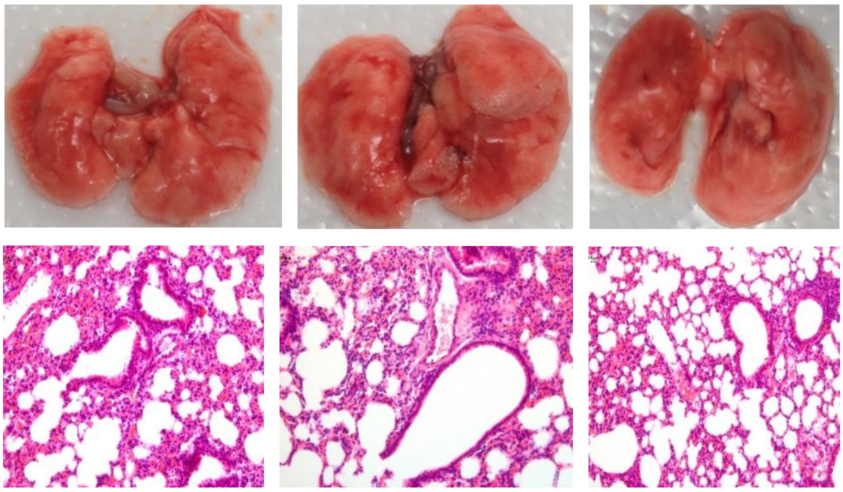

DAY7:（Pulmonary appearance and histopathologic examination of lung tissue ）

Blank

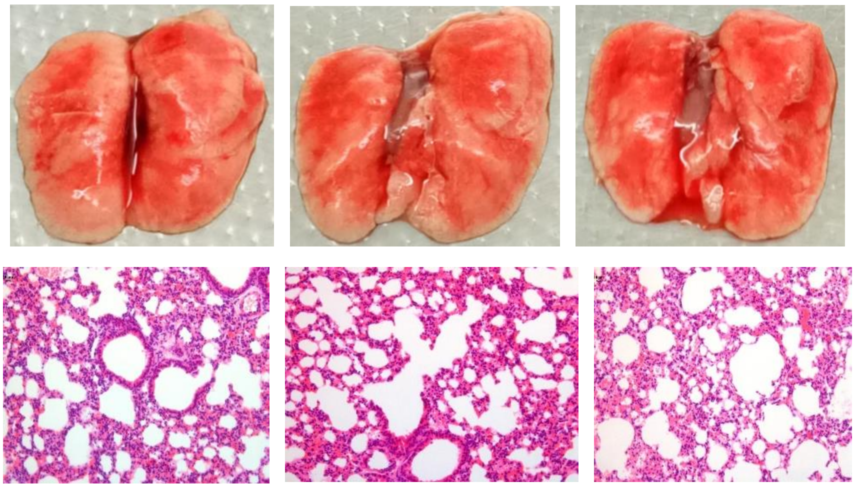

Model

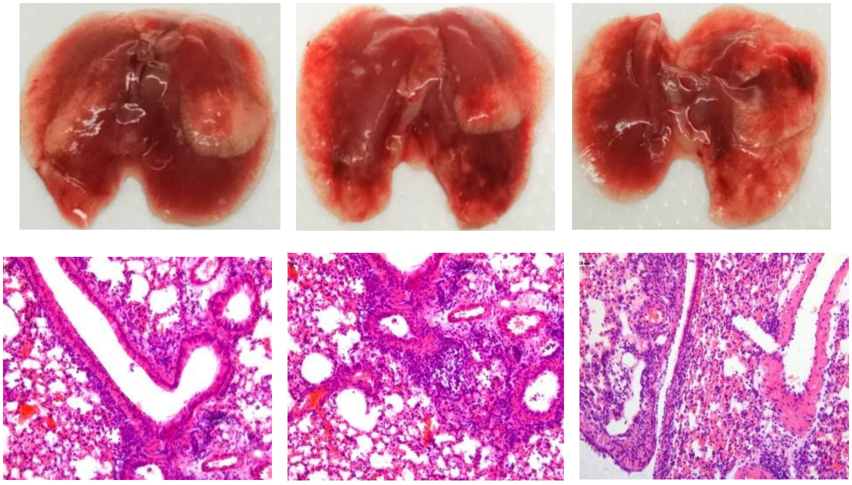

Oseltamivir (20 mg/kg/d)

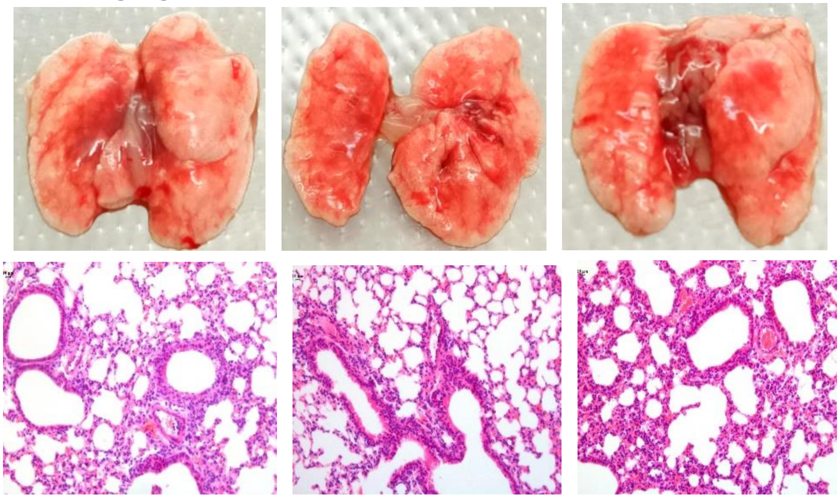

CSTRP (4200 mg/kg/d)

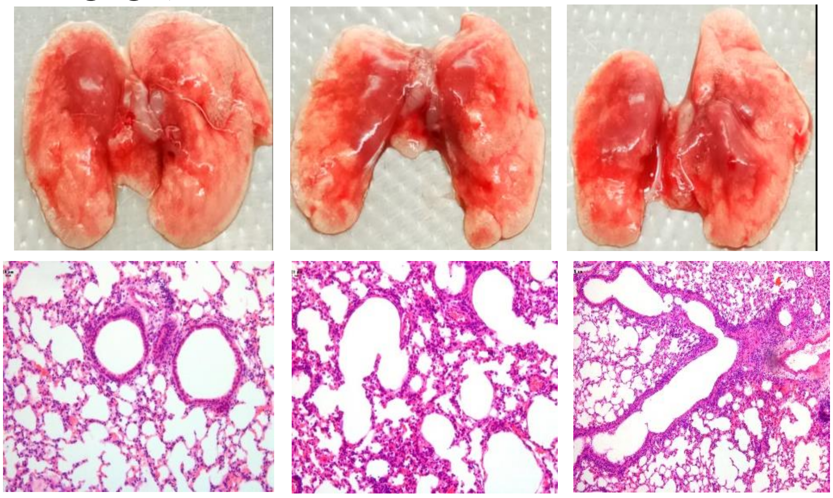

Supplement: Supplementary file 1 [file Data_Sheet_1.ZIP › Raw Data/Pulmonary appearance and histopathologic examination.pdf]
