# Supplementary material for: Anti-inflammatory effects of Chaishi Tuire Granules on influenza A treatment by mediating TRAF6/MAPK14 axis
Source: Front Med (Lausanne). 2022 Nov 14;9:943681. doi: 10.3389/fmed.2022.943681 (PMC9701735; doi:10.3389/fmed.2022.943681)

**Raw, uncropped Western blot image**

**Actin:**

**Blank**  **LPS CSTRP (mg/kg/d)**

**(0.5ug/ml) 1440 2880 5760**


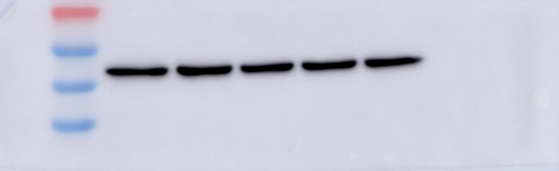


**TRAF6:**

**Blank**  **LPS CSTRP (mg/kg/d)**

**(0.5ug/ml) 1440 2880 5760**


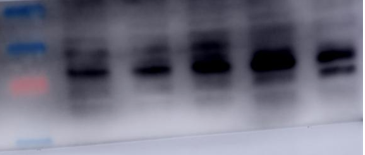


**MAPK14:**

**Blank**  **LPS CSTRP (mg/kg/d)**

**(0.5ug/ml) 1440 2880 5760**


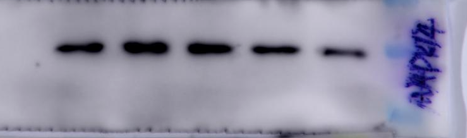

Supplement: Supplementary file 1 [file Data_Sheet_1.ZIP › Raw Data/Raw, uncropped Western blot image.docx]
