# Supplementary material for: Anti-inflammatory effects of Chaishi Tuire Granules on influenza A treatment by mediating TRAF6/MAPK14 axis
Source: Front Med (Lausanne). 2022 Nov 14;9:943681. doi: 10.3389/fmed.2022.943681 (PMC9701735; doi:10.3389/fmed.2022.943681)
Supplement: Supplementary file 1 [file Data_Sheet_1.ZIP › Raw Data/Supplementary Material/supplementary materials .docx]

**Supplementary Fig. S1：**

The CCK8 assay to determine the cell viability after CSTRP drug-containing serum treatment in RAW264.7 cells.


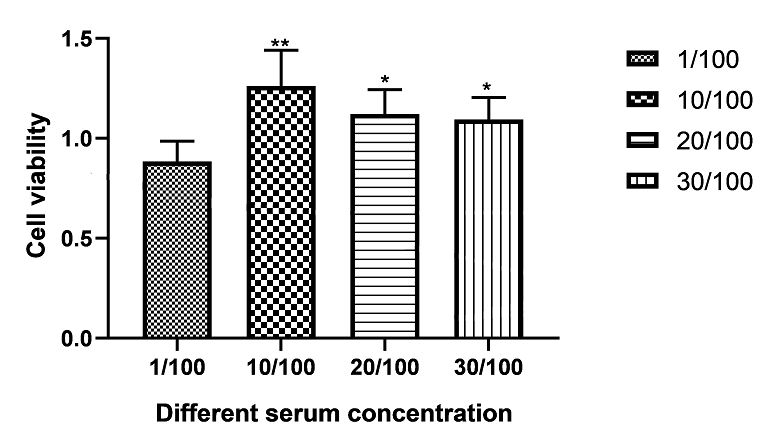


**Fig. S1** The results showed that even the 10% CSTRP (5760mg/kg/day) drug-containing serum gave no cytotoxicity in RAW264.7 cells

**Supplementary Fig. S2：**

To explored the release of LPS-induced TNF-α on different concentrations (0, 0.5, and 1 μg/mL) and different times (8, 12, 24, and 48 h) to find out the suitable acting condition.


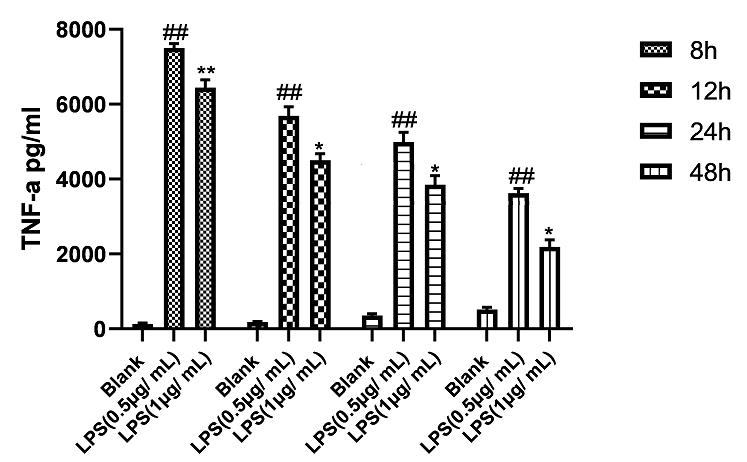


**Fig. S2** 0.5 μg/mL LPS treatment for 8 h was chosen for the following experiments.

**Supplementary Table S1：**

| **Primer/Probe** | **Sequence (5'-3')** |
| --- | --- |
| TRAF6-F | TGCTTTGCGTCCGTGCGATG |
| TRAF6-R | CCGTTACACTGCTGTGCTTCCA |
| MAPK14-F | CTGGCTCGGCACACTGATGATG |
| MAPK14-R | CCTCATGGCTTGGCATCCTGTT |
| GAPDH-F | ATGGTGAAGGTCGGTGTGAACG |
| GAPDH-R | CGCTCCTGGAAGATGGTGATGG |

**Supplementary Table S2：**

The compounds comprising the 9 kinds of Chinese medicine in CSTRP were obtained from the TCMSP database (not containing gypsum). A total of 168 candidate compounds after screening with ADME parameters (OB ≥ 30%, DL ≥ 0.14). The ultimate candidate compounds are shown in Supplementary Table S2.

| **TABLE S2 \| 168 active compounds in CSTRP decoction related to influenza.** | | | | | |
| --- | --- | --- | --- | --- | --- |
| **Mol ID** | **Molecule Name** | **MW** | **OB (%)** | **DL** | **Herb** |
| MOL001689 | acacetin | 284.28 | 34.97 | 0.24 | Scutellaria baicalensis |
| MOL000173 | wogonin | 284.28 | 30.68 | 0.23 | Scutellaria baicalensis |
| MOL000228 | (2R)-7-hydroxy-5-methoxy-2-phenylchroman-4-one | 270.30 | 55.23 | 0.20 | Scutellaria baicalensis |
| MOL002714 | baicalein | 270.25 | 33.52 | 0.21 | Scutellaria baicalensis |
| MOL002908 | 5,8,2'-Trihydroxy-7-methoxyflavone | 300.28 | 37.01 | 0.27 | Scutellaria baicalensis |
| MOL002909 | 5,7,2,5-tetrahydroxy-8,6-dimethoxyflavone | 376.34 | 33.82 | 0.45 | Scutellaria baicalensis |
| MOL002910 | Carthamidin | 288.27 | 41.15 | 0.24 | Scutellaria baicalensis |
| MOL002911 | 2,6,2',4'-tetrahydroxy-6'-methoxychaleone | 302.30 | 69.04 | 0.22 | Scutellaria baicalensis |
| MOL002913 | Dihydrobaicalin_qt | 272.27 | 40.04 | 0.21 | Scutellaria baicalensis |
| MOL002914 | Eriodyctiol (flavanone) | 288.27 | 41.35 | 0.24 | Scutellaria baicalensis |
| MOL002915 | Salvigenin | 328.34 | 49.07 | 0.33 | Scutellaria baicalensis |
| MOL002917 | 5,2',6'-Trihydroxy-7,8-dimethoxyflavone | 330.31 | 45.05 | 0.33 | Scutellaria baicalensis |
| MOL002925 | 5,7,2',6'-Tetrahydroxyflavone | 286.25 | 37.01 | 0.24 | Scutellaria baicalensis |
| MOL002926 | dihydrooroxylin A | 286.30 | 38.72 | 0.23 | Scutellaria baicalensis |
| MOL002927 | Skullcapflavone II | 374.37 | 69.51 | 0.44 | Scutellaria baicalensis |
| MOL002928 | oroxylin a | 284.28 | 41.37 | 0.23 | Scutellaria baicalensis |
| MOL002932 | Panicolin | 314.31 | 76.26 | 0.29 | Scutellaria baicalensis |
| MOL002933 | 5,7,4'-Trihydroxy-8-methoxyflavone | 300.28 | 36.56 | 0.27 | Scutellaria baicalensis |
| MOL002934 | NEOBAICALEIN | 374.37 | 104.34 | 0.44 | Scutellaria baicalensis |
| MOL002937 | DIHYDROOROXYLIN | 286.30 | 66.06 | 0.23 | Scutellaria baicalensis |
| MOL000358 | beta-sitosterol | 414.79 | 36.91 | 0.75 | Scutellaria baicalensis |
| MOL000359 | sitosterol | 414.79 | 36.91 | 0.75 | Scutellaria baicalensis |
| MOL000525 | Norwogonin | 270.25 | 39.40 | 0.21 | Scutellaria baicalensis |
| MOL000552 | 5,2'-Dihydroxy-6,7,8-trimethoxyflavone | 344.34 | 31.71 | 0.35 | Scutellaria baicalensis |
| MOL000073 | ent-Epicatechin | 290.29 | 48.96 | 0.24 | Scutellaria baicalensis |
| MOL000449 | Stigmasterol | 412.77 | 43.83 | 0.76 | Scutellaria baicalensis |
| MOL001458 | coptisine | 320.34 | 30.67 | 0.86 | Scutellaria baicalensis |
| MOL001490 | bis[(2S)-2-ethylhexyl] benzene-1,2-dicarboxylate | 390.62 | 43.59 | 0.35 | Scutellaria baicalensis |
| MOL001506 | Supraene | 410.80 | 33.55 | 0.42 | Scutellaria baicalensis |
| MOL002879 | Diop | 390.62 | 43.59 | 0.39 | Scutellaria baicalensis |
| MOL002897 | epiberberine | 336.39 | 43.09 | 0.78 | Scutellaria baicalensis |
| MOL008206 | Moslosooflavone | 298.31 | 44.09 | 0.25 | Scutellaria baicalensis |
| MOL010415 | 11,13-Eicosadienoic acid, methyl ester | 322.59 | 39.28 | 0.23 | Scutellaria baicalensis |
| MOL012245 | 5,7,4'-trihydroxy-6-methoxyflavanone | 302.30 | 36.63 | 0.27 | Scutellaria baicalensis |
| MOL012246 | 5,7,4'-trihydroxy-8-methoxyflavanone | 302.30 | 74.24 | 0.26 | Scutellaria baicalensis |
| MOL012266 | rivularin | 344.34 | 37.94 | 0.37 | Scutellaria baicalensis |
| MOL001494 | Mandenol | 308.56 | 42.00 | 74.00 | Lonicera japonica |
| MOL001495 | Ethyl linolenate | 306.54 | 46.10 | 75.00 | Lonicera japonica |
| MOL002707 | phytofluene | 543.02 | 43.18 | 76.00 | Lonicera japonica |
| MOL002914 | Eriodyctiol (flavanone) | 288.27 | 41.35 | 77.00 | Lonicera japonica |
| MOL003006 | (-)-(3R,8S,9R,9aS,10aS)-9-ethenyl-8-(beta-D-glucopyranosyloxy)-2,3,9,9a,10,10a-hexahydro-5-oxo-5H,8H-pyrano[4,3-d]oxazolo[3,2-a]pyridine-3-carboxylic acid_qt | 281.29 | 87.47 | 78.00 | Lonicera japonica |
| MOL003014 | secologanic dibutylacetal_qt | 384.57 | 53.65 | 0.29 | Lonicera japonica |
| MOL002773 | beta-carotene | 536.96 | 37.18 | 0.58 | Lonicera japonica |
| MOL003036 | ZINC03978781 | 412.77 | 43.83 | 0.76 | Lonicera japonica |
| MOL003044 | Chryseriol | 300.28 | 35.85 | 0.27 | Lonicera japonica |
| MOL003059 | kryptoxanthin | 552.96 | 47.25 | 0.57 | Lonicera japonica |
| MOL003062 | 4,5'-Retro-.beta.,.beta.-Carotene-3,3'-dione, 4',5'-didehydro- | 562.90 | 31.22 | 0.55 | Lonicera japonica |
| MOL003095 | 5-hydroxy-7-methoxy-2-(3,4,5-trimethoxyphenyl)chromone | 358.37 | 51.96 | 0.41 | Lonicera japonica |
| MOL003101 | 7-epi-Vogeloside | 432.47 | 46.13 | 0.58 | Lonicera japonica |
| MOL003108 | Caeruloside C | 550.57 | 55.64 | 0.73 | Lonicera japonica |
| MOL003111 | Centauroside_qt | 434.48 | 55.79 | 0.50 | Lonicera japonica |
| MOL003117 | Ioniceracetalides B_qt | 314.37 | 61.19 | 0.19 | Lonicera japonica |
| MOL003124 | XYLOSTOSIDINE | 415.51 | 43.17 | 0.64 | Lonicera japonica |
| MOL003128 | dinethylsecologanoside | 434.44 | 48.46 | 0.48 | Lonicera japonica |
| MOL000358 | beta-sitosterol | 414.79 | 36.91 | 0.75 | Lonicera japonica |
| MOL000422 | kaempferol | 286.25 | 41.88 | 0.24 | Lonicera japonica |
| MOL000449 | Stigmasterol | 412.77 | 43.83 | 0.76 | Lonicera japonica |
| MOL000006 | luteolin | 286.25 | 36.16 | 0.25 | Lonicera japonica |
| MOL000098 | quercetin | 302.25 | 46.43 | 0.28 | Lonicera japonica |
| MOL002235 | EUPATIN | 360.34 | 50.80 | 0.41 | rheum officinale |
| MOL002251 | Mutatochrome | 552.96 | 48.64 | 0.61 | rheum officinale |
| MOL002259 | Physciondiglucoside | 608.60 | 41.65 | 0.63 | rheum officinale |
| MOL002260 | Procyanidin B-5,3'-O-gallate | 730.67 | 31.99 | 0.32 | rheum officinale |
| MOL002268 | rhein | 284.23 | 47.07 | 0.28 | rheum officinale |
| MOL002276 | Sennoside E_qt | 524.50 | 50.69 | 0.61 | rheum officinale |
| MOL002280 | Torachrysone-8-O-beta-D-(6'-oxayl)-glucoside | 480.46 | 43.02 | 0.74 | rheum officinale |
| MOL002281 | Toralactone | 272.27 | 46.46 | 0.24 | rheum officinale |
| MOL002288 | Emodin-1-O-beta-D-glucopyranoside | 432.41 | 44.81 | 0.80 | rheum officinale |
| MOL002293 | Sennoside D_qt | 524.50 | 61.06 | 0.61 | rheum officinale |
| MOL002297 | Daucosterol_qt | 386.73 | 35.89 | 0.70 | rheum officinale |
| MOL002303 | palmidin A | 510.52 | 32.45 | 0.65 | rheum officinale |
| MOL000358 | beta-sitosterol | 414.79 | 36.91 | 0.75 | rheum officinale |
| MOL000471 | aloe-emodin | 270.25 | 83.38 | 0.24 | rheum officinale |
| MOL000554 | gallic acid-3-O-(6'-O-galloyl)-glucoside | 484.40 | 30.25 | 0.67 | rheum officinale |
| MOL000096 | (-)-catechin | 290.29 | 49.68 | 0.24 | rheum officinale |
| MOL001645 | Linoleyl acetate | 308.56 | 42.10 | 0.20 | Bupleuri Radix |
| MOL002776 | Baicalin | 446.39 | 40.12 | 0.75 | Bupleuri Radix |
| MOL000449 | Stigmasterol | 412.77 | 43.83 | 0.76 | Bupleuri Radix |
| MOL000354 | isorhamnetin | 316.28 | 49.60 | 0.31 | Bupleuri Radix |
| MOL000422 | kaempferol | 286.25 | 41.88 | 0.24 | Bupleuri Radix |
| MOL004598 | 3,5,6,7-tetramethoxy-2-(3,4,5-trimethoxyphenyl)chromone | 432.46 | 31.97 | 0.59 | Bupleuri Radix |
| MOL004609 | Areapillin | 360.34 | 48.96 | 0.41 | Bupleuri Radix |
| MOL013187 | Cubebin | 356.40 | 57.13 | 0.64 | Bupleuri Radix |
| MOL004624 | Longikaurin A | 348.48 | 47.72 | 0.53 | Bupleuri Radix |
| MOL004628 | Octalupine | 264.41 | 47.82 | 0.28 | Bupleuri Radix |
| MOL004644 | Sainfuran | 286.30 | 79.91 | 0.23 | Bupleuri Radix |
| MOL004648 | Troxerutin | 346.56 | 31.60 | 0.28 | Bupleuri Radix |
| MOL004653 | (+)-Anomalin | 426.50 | 46.06 | 0.66 | Bupleuri Radix |
| MOL004702 | saikosaponin c_qt | 472.78 | 30.50 | 0.63 | Bupleuri Radix |
| MOL004718 | α-spinasterol | 412.77 | 42.98 | 0.76 | Bupleuri Radix |
| MOL000490 | petunidin | 317.29 | 30.05 | 0.31 | Bupleuri Radix |
| MOL000098 | quercetin | 302.25 | 46.43 | 0.28 | Bupleuri Radix |
| MOL001689 | acacetin | 284.28 | 34.97 | 0.24 | radix isatidis |
| MOL002322 | isovitexin | 432.41 | 31.29 | 0.72 | radix isatidis |
| MOL001721 | Isaindigodione | 326.38 | 60.12 | 0.41 | radix isatidis |
| MOL001722 | 2-O-beta-D-glucopyranosyl-2H-1,4-benzoxazin-3(4H)-one | 327.32 | 43.62 | 0.31 | radix isatidis |
| MOL001726 | pinoresinol-4-O-beta-D-apiosyl-beta-D-glucopyranoside | 618.74 | 36.45 | 0.51 | radix isatidis |
| MOL001728 | 3-[ 2′ -( 5′ - hydroxymethyl) furyl ] -1 ( 2H) -isoquinolinone-7-O-BETA-D-glucoside_qt | 257.26 | 51.74 | 0.18 | radix isatidis |
| MOL001733 | EUPATORIN | 344.34 | 30.23 | 0.37 | radix isatidis |
| MOL001734 | 3-[[(2R,3R,5R,6S)-3,5-dihydroxy-6-(1H-indol-3-yloxy)-4-oxooxan-2-yl]methoxy]-3-oxopropanoic acid | 379.35 | 85.87 | 0.47 | radix isatidis |
| MOL001735 | Dinatin | 300.28 | 30.97 | 0.27 | radix isatidis |
| MOL001736 | (-)-taxifolin | 304.27 | 60.51 | 0.27 | radix isatidis |
| MOL001749 | ZINC03860434 | 390.62 | 43.59 | 0.35 | radix isatidis |
| MOL001750 | glucobrassicin | 448.52 | 66.02 | 0.48 | radix isatidis |
| MOL001755 | 24-Ethylcholest-4-en-3-one | 412.77 | 36.08 | 0.76 | radix isatidis |
| MOL001756 | quindoline | 218.27 | 33.17 | 0.22 | radix isatidis |
| MOL001767 | hydroxyindirubin | 278.28 | 63.37 | 0.30 | radix isatidis |
| MOL001769 | beta-sitosterol dodecantate | 597.13 | 34.57 | 0.57 | radix isatidis |
| MOL001771 | poriferast-5-en-3beta-ol | 414.79 | 36.91 | 0.75 | radix isatidis |
| MOL001774 | Ineketone | 318.50 | 37.14 | 0.30 | radix isatidis |
| MOL001779 | Sinoacutine | 327.41 | 49.11 | 0.46 | radix isatidis |
| MOL001781 | Indigo | 262.28 | 38.20 | 0.26 | radix isatidis |
| MOL001782 | (2Z)-2-(2-oxoindolin-3-ylidene)indolin-3-one | 262.28 | 48.40 | 0.26 | radix isatidis |
| MOL001783 | 2-(9-((3-methyl-2-oxopent-3-en-1-yl)oxy)-2-oxo-1,2,8,9-tetrahydrofuro[2,3-h]quinolin-8-yl)propan-2-yl acetate | 399.48 | 64.00 | 0.57 | radix isatidis |
| MOL001790 | Linarin | 592.60 | 39.84 | 0.71 | radix isatidis |
| MOL001792 | DFV | 256.27 | 32.76 | 0.18 | radix isatidis |
| MOL001793 | (E)-2-[(3-indole)cyanomethylene-]-3-indolinone | 300.36 | 54.59 | 0.32 | radix isatidis |
| MOL001798 | neohesperidin_qt | 302.30 | 71.17 | 0.27 | radix isatidis |
| MOL001800 | rosasterol | 414.79 | 35.87 | 0.75 | radix isatidis |
| MOL001803 | Sinensetin | 372.40 | 50.56 | 0.45 | radix isatidis |
| MOL001804 | Stigmasta-5,22-diene-3beta,7alpha-diol | 440.83 | 43.04 | 0.82 | radix isatidis |
| MOL001806 | Stigmasta-5,22-diene-3beta,7beta-diol | 454.86 | 42.56 | 0.83 | radix isatidis |
| MOL001810 | 6-(3-oxoindolin-2-ylidene)indolo[2,1-b]quinazolin-12-one | 363.39 | 45.28 | 0.89 | radix isatidis |
| MOL001814 | (E)-3-(3,5-dimethoxy-4-hydroxy-benzylidene)-2-indolinone | 297.33 | 57.18 | 0.25 | radix isatidis |
| MOL001820 | (E)-3-(3,5-dimethoxy-4-hydroxyb-enzylidene)-2-indolinone | 299.35 | 65.17 | 0.25 | radix isatidis |
| MOL001828 | 3-[(3,5-dimethoxy-4-oxo-1-cyclohexa-2,5-dienylidene)methyl]-2,4-dihydro-1H-pyrrolo[2,1-b]quinazolin-9-one | 350.40 | 51.84 | 0.56 | radix isatidis |
| MOL001833 | Glucobrassicin-1-Sulfonate_qt | 365.42 | 42.52 | 0.24 | radix isatidis |
| MOL000358 | beta-sitosterol | 414.79 | 36.91 | 0.75 | radix isatidis |
| MOL000359 | sitosterol | 414.79 | 36.91 | 0.75 | radix isatidis |
| MOL000449 | Stigmasterol | 412.77 | 43.83 | 0.76 | radix isatidis |
| MOL000953 | CLR | 386.73 | 37.87 | 0.68 | radix isatidis |
| MOL000173 | wogonin | 284.28 | 30.68 | 0.23 | Forsythia suspensa(Thunb, Vahl) |
| MOL003281 | 20(S)-dammar-24-ene-3β,20-diol-3-acetate | 486.86 | 40.23 | 0.82 | Forsythia suspensa(Thunb, Vahl) |
| MOL003283 | (2R,3R,4S)-4-(4-hydroxy-3-methoxy-phenyl)-7-methoxy-2,3-dimethylol-tetralin-6-ol | 360.44 | 66.51 | 0.39 | Forsythia suspensa(Thunb, Vahl) |
| MOL003290 | (3R,4R)-3,4-bis[(3,4-dimethoxyphenyl)methyl]oxolan-2-one | 386.48 | 52.30 | 0.48 | Forsythia suspensa(Thunb, Vahl) |
| MOL003295 | (+)-pinoresinol monomethyl ether | 372.45 | 53.08 | 0.57 | Forsythia suspensa(Thunb, Vahl) |
| MOL003305 | PHILLYRIN | 534.61 | 36.40 | 0.86 | Forsythia suspensa(Thunb, Vahl) |
| MOL003306 | ACon1_001697 | 372.45 | 85.12 | 0.57 | Forsythia suspensa(Thunb, Vahl) |
| MOL003308 | (+)-pinoresinol monomethyl ether-4-D-beta-glucoside_qt | 372.45 | 61.20 | 0.57 | Forsythia suspensa(Thunb, Vahl) |
| MOL003315 | 3beta-Acetyl-20,25-epoxydammarane-24alpha-ol | 502.86 | 33.07 | 0.79 | Forsythia suspensa(Thunb, Vahl) |
| MOL000211 | Mairin | 456.78 | 55.38 | 0.78 | Forsythia suspensa(Thunb, Vahl) |
| MOL003322 | FORSYTHINOL | 372.45 | 81.25 | 0.57 | Forsythia suspensa(Thunb, Vahl) |
| MOL003330 | (-)-Phillygenin | 372.45 | 95.04 | 0.57 | Forsythia suspensa(Thunb, Vahl) |
| MOL003344 | β-amyrin acetate | 468.84 | 42.06 | 0.74 | Forsythia suspensa(Thunb, Vahl) |
| MOL003347 | hyperforin | 536.87 | 44.03 | 0.60 | Forsythia suspensa(Thunb, Vahl) |
| MOL003348 | adhyperforin | 550.90 | 44.03 | 0.61 | Forsythia suspensa(Thunb, Vahl) |
| MOL003365 | Lactucasterol | 426.75 | 40.99 | 0.85 | Forsythia suspensa(Thunb, Vahl) |
| MOL003370 | Onjixanthone I | 302.30 | 79.16 | 0.30 | Forsythia suspensa(Thunb, Vahl) |
| MOL000358 | beta-sitosterol | 414.79 | 36.91 | 0.75 | Forsythia suspensa(Thunb, Vahl) |
| MOL000422 | kaempferol | 286.25 | 41.88 | 0.24 | Forsythia suspensa(Thunb, Vahl) |
| MOL000522 | arctiin | 534.61 | 34.45 | 0.84 | Forsythia suspensa(Thunb, Vahl) |
| MOL000006 | luteolin | 286.25 | 36.16 | 0.25 | Forsythia suspensa(Thunb, Vahl) |
| MOL000791 | bicuculline | 367.38 | 69.67 | 0.88 | Forsythia suspensa(Thunb, Vahl) |
| MOL000098 | quercetin | 302.25 | 46.43 | 0.28 | Forsythia suspensa(Thunb, Vahl) |
| MOL002235 | EUPATIN | 360.34 | 50.80 | 0.41 | Artemisia apiacea |
| MOL000354 | isorhamnetin | 316.28 | 49.60 | 0.31 | Artemisia apiacea |
| MOL000359 | sitosterol | 414.79 | 36.91 | 0.75 | Artemisia apiacea |
| MOL004083 | Tamarixetin | 316.28 | 32.86 | 0.31 | Artemisia apiacea |
| MOL004112 | Patuletin | 332.28 | 53.11 | 0.34 | Artemisia apiacea |
| MOL000422 | kaempferol | 286.25 | 41.88 | 0.24 | Artemisia apiacea |
| MOL000449 | Stigmasterol | 412.77 | 43.83 | 0.76 | Artemisia apiacea |
| MOL004609 | Areapillin | 360.34 | 48.96 | 0.41 | Artemisia apiacea |
| MOL005229 | Artemetin | 388.40 | 49.55 | 0.48 | Artemisia apiacea |
| MOL000006 | luteolin | 286.25 | 36.16 | 0.25 | Artemisia apiacea |
| MOL007274 | Skrofulein | 314.31 | 30.35 | 0.30 | Artemisia apiacea |
| MOL007389 | artemisitene | 280.35 | 54.36 | 0.31 | Artemisia apiacea |
| MOL007400 | vicenin-2_qt | 270.25 | 45.84 | 0.21 | Artemisia apiacea |
| MOL007401 | Cirsiliol | 330.31 | 43.46 | 0.34 | Artemisia apiacea |
| MOL007404 | vitexin_qt | 270.25 | 52.18 | 0.21 | Artemisia apiacea |
| MOL007412 | DMQT | 346.31 | 42.60 | 0.37 | Artemisia apiacea |
| MOL007415 | [(2S)-2-[[(2S)-2-(benzoylamino)-3-phenylpropanoyl]amino]-3-phenylpropyl] acetate | 444.57 | 58.02 | 0.52 | Artemisia apiacea |
| MOL007423 | 6,8-di-c-glucosylapigenin_qt | 270.25 | 59.85 | 0.21 | Artemisia apiacea |
| MOL007424 | artemisinin | 282.37 | 49.88 | 0.31 | Artemisia apiacea |
| MOL007425 | dihydroartemisinin | 284.39 | 50.75 | 0.30 | Artemisia apiacea |
| MOL007426 | deoxyartemisinin | 266.37 | 54.47 | 0.26 | Artemisia apiacea |
| MOL000098 | quercetin | 302.25 | 46.43 | 0.28 | Artemisia apiacea |
| MOL001677 | asperglaucide | 444.57 | 58.02 | 0.52 | rhizoma anemarrhenae |
| MOL003773 | Mangiferolic acid | 442.75 | 36.16 | 0.84 | rhizoma anemarrhenae |
| MOL000422 | kaempferol | 286.25 | 41.88 | 0.24 | rhizoma anemarrhenae |
| MOL004373 | Anhydroicaritin | 368.41 | 45.41 | 0.44 | rhizoma anemarrhenae |
| MOL004489 | Anemarsaponin F_qt | 432.71 | 60.06 | 0.79 | rhizoma anemarrhenae |
| MOL004492 | Chrysanthemaxanthin | 584.96 | 38.72 | 0.58 | rhizoma anemarrhenae |
| MOL004497 | Hippeastrine | 315.35 | 51.65 | 0.62 | rhizoma anemarrhenae |
| MOL004514 | Timosaponin B III_qt | 416.71 | 35.26 | 0.87 | rhizoma anemarrhenae |
| MOL000449 | Stigmasterol | 412.77 | 43.83 | 0.76 | rhizoma anemarrhenae |
| MOL004528 | Icariin I | 676.73 | 41.58 | 0.61 | rhizoma anemarrhenae |
| MOL004540 | Anemarsaponin C_qt | 416.71 | 35.50 | 0.87 | rhizoma anemarrhenae |
| MOL004542 | Anemarsaponin E_qt | 448.76 | 30.67 | 0.86 | rhizoma anemarrhenae |
| MOL000483 | (Z)-3-(4-hydroxy-3-methoxy-phenyl)-N-[2-(4-hydroxyphenyl)ethyl]acrylamide | 313.38 | 118.35 | 0.26 | rhizoma anemarrhenae |
| MOL000546 | diosgenin | 414.69 | 80.88 | 0.81 | rhizoma anemarrhenae |
| MOL000631 | coumaroyltyramine | 283.35 | 112.90 | 0.20 | rhizoma anemarrhenae |
| MOL006554 | Taraxerol | 426.80 | 38.40 | 0.77 | Dandelion |
| MOL000098 | quercetin | 302.25 | 46.43 | 0.28 | Dandelion |
| MOL002680 | Flavoxanthin | 584.96 | 60.41 | 0.56 | Dandelion |
| MOL004492 | Chrysanthemaxanthin | 584.96 | 38.72 | 0.58 | Dandelion |

**Supplementary Table S3：**

Based on the GENECARDS and OMIM databases, a total of 810 targets directly and indirectly associated with influenza were obtained supplementary Table S3.

| **Gene Symbol** | **GC Id** | **Source** |
| --- | --- | --- |
| NEU1 | GC06M031857 | GENECARDS |
| IRF7 | GC11M000612 | GENECARDS |
| DDX58 | GC09M032455 | GENECARDS |
| TNF | GC06P047305 | GENECARDS |
| IFNB1 | GC09M021077 | GENECARDS |
| IFNA1 | GC09P021494 | GENECARDS |
| MX1 | GC21P041420 | GENECARDS |
| GP2 | GC16M020309 | GENECARDS |
| CD4 | GC12P006786 | GENECARDS |
| BACE1 | GC11M117285 | GENECARDS |
| CA1 | GC08M085327 | GENECARDS |
| CD8A | GC02M086784 | GENECARDS |
| TRIM25 | GC17M056836 | GENECARDS |
| ST6GAL1 | GC03P186930 | GENECARDS |
| TGM3 | GC20P002276 | GENECARDS |
| POLI | GC18P054274 | GENECARDS |
| HAS1 | GC19M051714 | GENECARDS |
| RAB39B | GC0XM155259 | GENECARDS |
| IFI30 | GC19P018173 | GENECARDS |
| LGALS7 | GC19M038770 | GENECARDS |
| SCARA3 | GC08P027633 | GENECARDS |
| IFNA17 | GC09M021227 | GENECARDS |
| LGALS7B | GC19P038790 | GENECARDS |
| IFITM3 | GC11M000319 | GENECARDS |
| TMPRSS2 | GC21M041464 | GENECARDS |
| PML | GC15P073994 | GENECARDS |
| IL1B | GC02M112829 | GENECARDS |
| IL6 | GC07P022765 | GENECARDS |
| IL10 | GC01M206767 | GENECARDS |
| CCR5 | GC03P046383 | GENECARDS |
| IL1A | GC02M112773 | GENECARDS |
| CXCL10 | GC04M076021 | GENECARDS |
| HIF1A | GC14P061695 | GENECARDS |
| FCGR2A | GC01P161505 | GENECARDS |
| CALCA | GC11M014945 | GENECARDS |
| CCL2 | GC17P034255 | GENECARDS |
| TGFB1 | GC19M041301 | GENECARDS |
| HLA-G | GC06P047256 | GENECARDS |
| CD209 | GC19M007739 | GENECARDS |
| TLR3 | GC04P186059 | GENECARDS |
| SFTPD | GC10M079937 | GENECARDS |
| SFTPB | GC02M085657 | GENECARDS |
| IL21 | GC04M122612 | GENECARDS |
| PTPN11 | GC12P112418 | GENECARDS |
| PRPF8 | GC17M001650 | GENECARDS |
| HLA-B | GC06M031315 | GENECARDS |
| MBL2 | GC10M052760 | GENECARDS |
| HLA-DRB1 | GC06M032578 | GENECARDS |
| CLK1 | GC02M200853 | GENECARDS |
| SON | GC21P033542 | GENECARDS |
| FN1 | GC02M215360 | GENECARDS |
| CD55 | GC01P207321 | GENECARDS |
| MRC1 | GC10P017809 | GENECARDS |
| SOCS1 | GC16M011255 | GENECARDS |
| DLG2 | GC11M083455 | GENECARDS |
| F2 | GC11P046720 | GENECARDS |
| IL21R | GC16P027413 | GENECARDS |
| GATAD2A | GC19P023337 | GENECARDS |
| MIR150 | GC19M049500 | GENECARDS |
| PRKRA | GC02M178431 | GENECARDS |
| STAU1 | GC20M049113 | GENECARDS |
| NAMPT | GC07M106248 | GENECARDS |
| PLCG1 | GC20P041136 | GENECARDS |
| FST | GC05P053480 | GENECARDS |
| CD274 | GC09P005450 | GENECARDS |
| MIR939 | GC08M144394 | GENECARDS |
| PLD2 | GC17P004808 | GENECARDS |
| XPO1 | GC02M061445 | GENECARDS |
| NXF1 | GC11M063441 | GENECARDS |
| BCL2 | GC18M063123 | GENECARDS |
| CASP3 | GC04M184627 | GENECARDS |
| BAX | GC19P048954 | GENECARDS |
| NFE2L2 | GC02M177227 | GENECARDS |
| HLA-A | GC06P047265 | GENECARDS |
| ST3GAL1 | GC08M133454 | GENECARDS |
| HMGB1 | GC13M030456 | GENECARDS |
| TTR | GC18P031557 | GENECARDS |
| VEGFA | GC06P043770 | GENECARDS |
| CRP | GC01M159716 | GENECARDS |
| RSAD2 | GC02P006865 | GENECARDS |
| IVNS1ABP | GC01M185295 | GENECARDS |
| ABCB1 | GC07M087504 | GENECARDS |
| DNMT1 | GC19M010133 | GENECARDS |
| ACE2 | GC0XM015562 | GENECARDS |
| PLG | GC06P160702 | GENECARDS |
| PIK3CG | GC07P106865 | GENECARDS |
| HLA-DQB1 | GC06M032804 | GENECARDS |
| IL10RB | GC21P033266 | GENECARDS |
| P2RY11 | GC19P010149 | GENECARDS |
| MPP2 | GC17M043875 | GENECARDS |
| ZNF365 | GC10P062374 | GENECARDS |
| PPAN | GC19P010107 | GENECARDS |
| PPAN-P2RY11 | GC19P010108 | GENECARDS |
| KIR2DS5 | GC19Mr00079 | GENECARDS |
| KIR2DL5A | GC19MR00046 | GENECARDS |
| KIR3DS1 | GC19MR00058 | GENECARDS |
| KIR2DL5B | GC19MP00133 | GENECARDS |
| TRA | GC14P021621 | GENECARDS |
| SNORD105 | GC19P010150 | GENECARDS |
| TRB | GC07P145138 | GENECARDS |
| ELANE | GC19P000854 | GENECARDS |
| TXN | GC09M110243 | GENECARDS |
| KPNA6 | GC01P032108 | GENECARDS |
| MIR146A | GC05P160485 | GENECARDS |
| MMP9 | GC20P046008 | GENECARDS |
| MYC | GC08P127735 | GENECARDS |
| JUN | GC01M058780 | GENECARDS |
| B2M | GC15P044711 | GENECARDS |
| CYP17A1 | GC10M102830 | GENECARDS |
| CDKN1B | GC12P012716 | GENECARDS |
| HSPD1 | GC02M197486 | GENECARDS |
| ISG15 | GC01P001001 | GENECARDS |
| ATP6V1B2 | GC08P020197 | GENECARDS |
| PRPS1 | GC0XP107628 | GENECARDS |
| ATP6AP2 | GC0XP040582 | GENECARDS |
| ATP6V1A | GC03P113747 | GENECARDS |
| COPB2 | GC03M139355 | GENECARDS |
| PSENEN | GC19P038231 | GENECARDS |
| RPS14 | GC05M150443 | GENECARDS |
| PTPRN | GC02M219289 | GENECARDS |
| TNK2 | GC03M195863 | GENECARDS |
| SF3B1 | GC02M197389 | GENECARDS |
| GCLC | GC06M053497 | GENECARDS |
| AP2M1 | GC03P184174 | GENECARDS |
| ATP6V0D1 | GC16M067438 | GENECARDS |
| DBT | GC01M100186 | GENECARDS |
| EIF4A3 | GC17M080135 | GENECARDS |
| RETN | GC19P007669 | GENECARDS |
| KPNB1 | GC17P047649 | GENECARDS |
| TXNL4A | GC18M079970 | GENECARDS |
| TFE3 | GC0XM049028 | GENECARDS |
| NUP98 | GC11M003671 | GENECARDS |
| NAA10 | GC0XM153929 | GENECARDS |
| ATP6AP1 | GC0XP154428 | GENECARDS |
| ATP6V0C | GC16P002513 | GENECARDS |
| EIF3A | GC10M119034 | GENECARDS |
| COPA | GC01M160288 | GENECARDS |
| HSF4 | GC16P067164 | GENECARDS |
| RPS5 | GC19P058386 | GENECARDS |
| LHX3 | GC09M136196 | GENECARDS |
| WNT9A | GC01M227920 | GENECARDS |
| CD58 | GC01M116514 | GENECARDS |
| OPN1SW | GC07M128772 | GENECARDS |
| AKTIP | GC16M053492 | GENECARDS |
| CHST5 | GC16M075528 | GENECARDS |
| TBL3 | GC16P002426 | GENECARDS |
| ITLN1 | GC01M160876 | GENECARDS |
| KANSL1 | GC17M046031 | GENECARDS |
| ATCAY | GC19P003880 | GENECARDS |
| SNRNP70 | GC19P049085 | GENECARDS |
| SAFB | GC19P005623 | GENECARDS |
| LARP1 | GC05P154682 | GENECARDS |
| XAB2 | GC19M007619 | GENECARDS |
| MED6 | GC14M070581 | GENECARDS |
| NUP205 | GC07P135557 | GENECARDS |
| UBAC2 | GC13P099200 | GENECARDS |
| TRERF1 | GC06M042224 | GENECARDS |
| COPG1 | GC03P129249 | GENECARDS |
| TSSK6 | GC19M019514 | GENECARDS |
| WDR83 | GC19P012666 | GENECARDS |
| DTX3 | GC12P057604 | GENECARDS |
| PRSS27 | GC16M002713 | GENECARDS |
| SF3B6 | GC02M024067 | GENECARDS |
| TRIM60 | GC04P165032 | GENECARDS |
| BARHL2 | GC01M090644 | GENECARDS |
| TRMT61A | GC14P103529 | GENECARDS |
| PPP1R14D | GC15M040815 | GENECARDS |
| PLPPR4 | GC01P099262 | GENECARDS |
| H2BC15 | GC06P028802 | GENECARDS |
| TSPOAP1 | GC17M058302 | GENECARDS |
| EPB41L4A-DT | GC05P112421 | GENECARDS |
| TRIM21 | GC11M004384 | GENECARDS |
| PRPF19 | GC11M060890 | GENECARDS |
| MIRLET7C | GC21P016551 | GENECARDS |
| IFITM1 | GC11P000313 | GENECARDS |
| IFITM2 | GC11P000300 | GENECARDS |
| NCR1 | GC19P054906 | GENECARDS |
| SFTPA2 | GC10M079563 | GENECARDS |
| PLAU | GC10P073909 | GENECARDS |
| HPGD | GC04M174490 | GENECARDS |
| RUNX1 | GC21M034787 | GENECARDS |
| ATP1A2 | GC01P160115 | GENECARDS |
| CD81 | GC11P002377 | GENECARDS |
| PIN1 | GC19P009835 | GENECARDS |
| KIF11 | GC10P092593 | GENECARDS |
| CEL | GC09P133061 | GENECARDS |
| MYOD1 | GC11P017741 | GENECARDS |
| SIGMAR1 | GC09M034634 | GENECARDS |
| ATP2C1 | GC03P130850 | GENECARDS |
| NEK9 | GC14M075079 | GENECARDS |
| PIK3R5 | GC17M008878 | GENECARDS |
| TCF3 | GC19M001609 | GENECARDS |
| CYP2U1 | GC04P107931 | GENECARDS |
| EPHB6 | GC07P144968 | GENECARDS |
| GRIN2C | GC17M074842 | GENECARDS |
| CRYAA | GC21P043169 | GENECARDS |
| IL17RA | GC22P017086 | GENECARDS |
| PSMA1 | GC11M014505 | GENECARDS |
| RPL35 | GC09M124857 | GENECARDS |
| RPS10 | GC06M042209 | GENECARDS |
| NTHL1 | GC16M002192 | GENECARDS |
| SLC22A6 | GC11M063458 | GENECARDS |
| LINGO1 | GC15M077613 | GENECARDS |
| LONP1 | GC19M005691 | GENECARDS |
| AHCYL1 | GC01P109984 | GENECARDS |
| MATN3 | GC02M019992 | GENECARDS |
| MAN2B1 | GC19M012663 | GENECARDS |
| FERMT3 | GC11P064206 | GENECARDS |
| EEF1A1 | GC06M073515 | GENECARDS |
| SNW1 | GC14M077717 | GENECARDS |
| RPS27A | GC02P055231 | GENECARDS |
| PLK3 | GC01P044799 | GENECARDS |
| PSMD14 | GC02P161308 | GENECARDS |
| IRF2 | GC04M184387 | GENECARDS |
| KCNJ12 | GC17P026750 | GENECARDS |
| PSMD2 | GC03P184298 | GENECARDS |
| TNFRSF18 | GC01M001203 | GENECARDS |
| SELPLG | GC12M108621 | GENECARDS |
| RACGAP1 | GC12M049978 | GENECARDS |
| XPNPEP1 | GC10M109864 | GENECARDS |
| CLIC4 | GC01P024745 | GENECARDS |
| NEK8 | GC17P028725 | GENECARDS |
| SNX9 | GC06P157685 | GENECARDS |
| POLR2L | GC11M000829 | GENECARDS |
| APBB1IP | GC10P026462 | GENECARDS |
| ARTN | GC01P043933 | GENECARDS |
| DCLK2 | GC04P150078 | GENECARDS |
| CXCR6 | GC03P045982 | GENECARDS |
| RPS16 | GC19M039433 | GENECARDS |
| POLR2H | GC03P184361 | GENECARDS |
| RAB6B | GC03M133824 | GENECARDS |
| KATNB1 | GC16P057735 | GENECARDS |
| SULF2 | GC20M047656 | GENECARDS |
| VNN2 | GC06M132743 | GENECARDS |
| BAIAP3 | GC16P001333 | GENECARDS |
| FAU | GC11M065120 | GENECARDS |
| CD48 | GC01M160648 | GENECARDS |
| DHRS2 | GC14P025289 | GENECARDS |
| RFFL | GC17M035006 | GENECARDS |
| EIF3C | GC16P028709 | GENECARDS |
| SF3A1 | GC22M030331 | GENECARDS |
| PHF2 | GC09P093576 | GENECARDS |
| STAB1 | GC03P052495 | GENECARDS |
| ATG13 | GC11P046623 | GENECARDS |
| EIF3G | GC19M010115 | GENECARDS |
| DMAP1 | GC01P044214 | GENECARDS |
| COPB1 | GC11M014436 | GENECARDS |
| PCDH18 | GC04M137518 | GENECARDS |
| SNRPF | GC12P095858 | GENECARDS |
| SUPT6H | GC17P028662 | GENECARDS |
| CCNB3 | GC0XP050202 | GENECARDS |
| FCHO2 | GC05P072955 | GENECARDS |
| CNNM1 | GC10P099330 | GENECARDS |
| SNX6 | GC14M034561 | GENECARDS |
| SMU1 | GC09M033041 | GENECARDS |
| TRIM14 | GC09M098035 | GENECARDS |
| RNF150 | GC04M140859 | GENECARDS |
| GNRH2 | GC20P003080 | GENECARDS |
| CLSTN3 | GC12P008242 | GENECARDS |
| ENGASE | GC17P079071 | GENECARDS |
| ZBTB2 | GC06M151364 | GENECARDS |
| SRRM2 | GC16P004234 | GENECARDS |
| MAP3K7CL | GC21P029077 | GENECARDS |
| FBXW10 | GC17P018832 | GENECARDS |
| CLUH | GC17M002689 | GENECARDS |
| GPR146 | GC07P001044 | GENECARDS |
| TAMM41 | GC03M011721 | GENECARDS |
| RBM42 | GC19P038222 | GENECARDS |
| CRAMP1 | GC16P001644 | GENECARDS |
| STYXL2 | GC01P167095 | GENECARDS |
| PIK3R2 | GC19P018153 | GENECARDS |
| IRF3 | GC19M049659 | GENECARDS |
| NEU2 | GC02P233032 | GENECARDS |
| ICAM1 | GC19P010270 | GENECARDS |
| BRAF | GC07M140719 | GENECARDS |
| GATA3 | GC10P008045 | GENECARDS |
| AHR | GC07P016916 | GENECARDS |
| NR2F2 | GC15P096325 | GENECARDS |
| MITF | GC03P069788 | GENECARDS |
| PNLIP | GC10P116545 | GENECARDS |
| SLC18A2 | GC10P117241 | GENECARDS |
| GABRG2 | GC05P162000 | GENECARDS |
| DMD | GC0XM031047 | GENECARDS |
| SLC12A5 | GC20P046021 | GENECARDS |
| ADAM12 | GC10M126012 | GENECARDS |
| PTPRB | GC12M070516 | GENECARDS |
| TRPS1 | GC08M115408 | GENECARDS |
| PIP4K2A | GC10M022484 | GENECARDS |
| B4GALT1 | GC09M033100 | GENECARDS |
| NANS | GC09P098056 | GENECARDS |
| SLC1A4 | GC02P064988 | GENECARDS |
| PTGFR | GC01P078303 | GENECARDS |
| TRPC4 | GC13M037636 | GENECARDS |
| TFDP1 | GC13P113584 | GENECARDS |
| FGF12 | GC03M192139 | GENECARDS |
| CTNNA2 | GC02P079185 | GENECARDS |
| CLCN4 | GC0XP010085 | GENECARDS |
| POLR3B | GC12P106357 | GENECARDS |
| PLTP | GC20M045898 | GENECARDS |
| SLC30A2 | GC01M026048 | GENECARDS |
| NUMA1 | GC11M072002 | GENECARDS |
| FANCM | GC14P045135 | GENECARDS |
| MEIS1 | GC02P066433 | GENECARDS |
| GTF2IRD1 | GC07P074461 | GENECARDS |
| CCNG1 | GC05P163438 | GENECARDS |
| PDCD6IP | GC03P033798 | GENECARDS |
| NRG3 | GC10P083672 | GENECARDS |
| SPRED2 | GC02M065307 | GENECARDS |
| ADD2 | GC02M070626 | GENECARDS |
| APLP1 | GC19P038238 | GENECARDS |
| MDC1 | GC06M030896 | GENECARDS |
| FKBP1B | GC02P024033 | GENECARDS |
| ACSL3 | GC02P222860 | GENECARDS |
| GTF2H4 | GC06P047289 | GENECARDS |
| CEP290 | GC12M088049 | GENECARDS |
| MMP21 | GC10M125766 | GENECARDS |
| DDX18 | GC02P117878 | GENECARDS |
| PTPRR | GC12M070638 | GENECARDS |
| DHX16 | GC06M030653 | GENECARDS |
| MICA | GC06P031399 | GENECARDS |
| LRP1B | GC02M140231 | GENECARDS |
| MAN1C1 | GC01P025631 | GENECARDS |
| AGFG1 | GC02P227473 | GENECARDS |
| ABCF1 | GC06P030571 | GENECARDS |
| EXTL1 | GC01P026032 | GENECARDS |
| DPP10 | GC02P114442 | GENECARDS |
| CD207 | GC02M070830 | GENECARDS |
| DDX39B | GC06M031530 | GENECARDS |
| PCDH7 | GC04P030722 | GENECARDS |
| ZFP57 | GC06M029672 | GENECARDS |
| LZTS1 | GC08M020246 | GENECARDS |
| PPP1R10 | GC06M030600 | GENECARDS |
| ENPP6 | GC04M184088 | GENECARDS |
| KCNU1 | GC08P036784 | GENECARDS |
| CNKSR1 | GC01P026178 | GENECARDS |
| GHITM | GC10P084139 | GENECARDS |
| GIPC2 | GC01P077987 | GENECARDS |
| ADGRL2 | GC01P081306 | GENECARDS |
| HLA-DQA2 | GC06P032741 | GENECARDS |
| NFKBIL1 | GC06P047302 | GENECARDS |
| SLITRK5 | GC13P087671 | GENECARDS |
| TUBGCP2 | GC10M133278 | GENECARDS |
| TRIM39 | GC06P047275 | GENECARDS |
| NCAPG2 | GC07M158631 | GENECARDS |
| TRIM26 | GC06M030184 | GENECARDS |
| VAX2 | GC02P070900 | GENECARDS |
| GNL1 | GC06M030541 | GENECARDS |
| ARAP2 | GC04M035950 | GENECARDS |
| FIGLA | GC02M070741 | GENECARDS |
| ESYT2 | GC07M158730 | GENECARDS |
| BUD13 | GC11M116749 | GENECARDS |
| CORO2A | GC09M098120 | GENECARDS |
| DRAP1 | GC11P065926 | GENECARDS |
| MRPS18B | GC06P030617 | GENECARDS |
| SPAG6 | GC10P022345 | GENECARDS |
| IGSF11 | GC03M118900 | GENECARDS |
| SNX13 | GC07M017798 | GENECARDS |
| L3MBTL3 | GC06P130013 | GENECARDS |
| TCF19 | GC06P047294 | GENECARDS |
| TBC1D2 | GC09M098198 | GENECARDS |
| TEKT1 | GC17M006789 | GENECARDS |
| DHX32 | GC10M125836 | GENECARDS |
| KIF15 | GC03P045351 | GENECARDS |
| LINGO2 | GC09M027940 | GENECARDS |
| AFTPH | GC02P064524 | GENECARDS |
| ARPP21 | GC03P035680 | GENECARDS |
| CSMD3 | GC08M112223 | GENECARDS |
| NEUROD4 | GC12P055023 | GENECARDS |
| PFN4 | GC02M024115 | GENECARDS |
| PXDNL | GC08M051319 | GENECARDS |
| TMCO3 | GC13P113490 | GENECARDS |
| TTLL7 | GC01M083865 | GENECARDS |
| RPL10L | GC14M046651 | GENECARDS |
| ZNF703 | GC08P037695 | GENECARDS |
| ARRDC4 | GC15P097960 | GENECARDS |
| RIC8B | GC12P106774 | GENECARDS |
| PNLIPRP3 | GC10P116427 | GENECARDS |
| FANK1 | GC10P125896 | GENECARDS |
| CLEC4F | GC02M070808 | GENECARDS |
| SPATA8 | GC15P096783 | GENECARDS |
| SPINK4 | GC09P033230 | GENECARDS |
| SERHL2 | GC22P042553 | GENECARDS |
| SAMD3 | GC06M130144 | GENECARDS |
| SLITRK4 | GC0XM143622 | GENECARDS |
| UBXN2A | GC02P023927 | GENECARDS |
| WWC3 | GC0XP010015 | GENECARDS |
| ATAD2B | GC02M023711 | GENECARDS |
| CEP128 | GC14M080476 | GENECARDS |
| MUC21 | GC06P047291 | GENECARDS |
| NRM | GC06M030895 | GENECARDS |
| SELENON | GC01P025800 | GENECARDS |
| KLHL29 | GC02P023348 | GENECARDS |
| DYNC2I1 | GC07P158839 | GENECARDS |
| POLR1H | GC06P048997 | GENECARDS |
| PSORS1C2 | GC06M031137 | GENECARDS |
| TMEM200A | GC06P130365 | GENECARDS |
| C6orf136 | GC06P047284 | GENECARDS |
| SERTAD2 | GC02M064631 | GENECARDS |
| TSGA10IP | GC11P065947 | GENECARDS |
| TMEM263 | GC12P106955 | GENECARDS |
| OR9K2 | GC12P055129 | GENECARDS |
| MEDAG | GC13P030906 | GENECARDS |
| MTERF2 | GC12M106977 | GENECARDS |
| TMPRSS11F | GC04M068053 | GENECARDS |
| TRMO | GC09M097896 | GENECARDS |
| CFAP74 | GC01M001921 | GENECARDS |
| HLA-H | GC06P047258 | GENECARDS |
| MUC22 | GC06P031005 | GENECARDS |
| HLA-J | GC06P047267 | GENECARDS |
| MIR877 | GC06P030584 | GENECARDS |
| HCG18 | GC06M030874 | GENECARDS |
| SFTA1P | GC10M010784 | GENECARDS |
| TUSC7 | GC03P116709 | GENECARDS |
| HLA-F-AS1 | GC06M030844 | GENECARDS |
| HCG4B | GC06M030862 | GENECARDS |
| TEX26-AS1 | GC13M030881 | GENECARDS |
| MIR622 | GC13P090231 | GENECARDS |
| HLA-L | GC06P047274 | GENECARDS |
| RRP7BP | GC22M042555 | GENECARDS |
| MDFIC2 | GC03M070197 | GENECARDS |
| HLA-K | GC06P047263 | GENECARDS |
| LOC339166 | GC17P005773 | GENECARDS |
| ATP6V1B1-AS1 | GC02M070941 | GENECARDS |
| PRANCR | GC12M069901 | GENECARDS |
| LINC00709 | GC10P009275 | GENECARDS |
| MICE | GC06M030847 | GENECARDS |
| ETF1P1 | GC06P047268 | GENECARDS |
| HLA-W | GC06P047261 | GENECARDS |
| LINC02153 | GC08P020974 | GENECARDS |
| LINC02889 | GC07M017410 | GENECARDS |
| LINC02254 | GC15M103983 | GENECARDS |
| HLA-U | GC06P047264 | GENECARDS |
| PAIP1P1 | GC06M030186 | GENECARDS |
| ENSG00000204929 | GC02P065439 | GENECARDS |
| RPL3P2 | GC06P031280 | GENECARDS |
| ENSG00000233290 | GC01M082215 | GENECARDS |
| ENSG00000234255 | GC02M065442 | GENECARDS |
| SUMO2P1 | GC06M029639 | GENECARDS |
| ENSG00000254186 | GC05M162425 | GENECARDS |
| LINC01811 | GC03P033956 | GENECARDS |
| ENSG00000260329 | GC12M106954 | GENECARDS |
| RNA5SP22 | GC01M078094 | GENECARDS |
| RNA5SP328 | GC10M126145 | GENECARDS |
| HMGB3P8 | GC10M116442 | GENECARDS |
| ENSG00000239268 | GC03M117672 | GENECARDS |
| ENSG00000233478 | GC01M025644 | GENECARDS |
| ENSG00000228675 | GC07P017719 | GENECARDS |
| ENSG00000227678 | GC06M130133 | GENECARDS |
| ENSG00000232353 | GC03P192029 | GENECARDS |
| ENSG00000233258 | GC10M083881 | GENECARDS |
| ENSG00000254092 | GC08M020952 | GENECARDS |
| TRP-GGG1-1 | GC10P022563 | GENECARDS |
| ENSG00000254260 | GC08P020941 | GENECARDS |
| OR9K1P | GC12P055115 | GENECARDS |
| ENSG00000253344 | GC08M037626 | GENECARDS |
| ENSG00000272540 | GC06M030903 | GENECARDS |
| LINC02253 | GC15P097771 | GENECARDS |
| LINC02334 | GC13P037935 | GENECARDS |
| LINC02336 | GC13P089402 | GENECARDS |
| ENSG00000285471 | GC17P005775 | GENECARDS |
| RN7SKP181 | GC15P096842 | GENECARDS |
| DHFRP3 | GC02P082856 | GENECARDS |
| RPL30P12 | GC12P106910 | GENECARDS |
| HMGN2P21 | GC02P070803 | GENECARDS |
| SEC63P2 | GC04P035491 | GENECARDS |
| ENSG00000237498 | GC02M082479 | GENECARDS |
| ENSG00000223808 | GC10P008943 | GENECARDS |
| LOC105379752 | GC07M158777 | GENECARDS |
| ENSG00000258845 | GC14P045567 | GENECARDS |
| ENSG00000233870 | GC02P071178 | GENECARDS |
| RF00017-7760 | GC09P097907 | GENECARDS |
| ENSG00000287981 | GC03P034152 | GENECARDS |
| lnc-C10orf90-5 | GC10M126242 | GENECARDS |
| lnc-FRMD4B-4 | GC03M069763 | GENECARDS |
| lnc-WDR60-13 | GC07P158829 | GENECARDS |
| MN298639 | GC09M098376 | GENECARDS |
| piR-50443-383 | GC09P098243 | GENECARDS |
| lnc-EBLN1-6 | GC10M022536 | GENECARDS |
| lnc-DHX32-4 | GC10M126119 | GENECARDS |
| lnc-ESYT2-1 | GC07M158793 | GENECARDS |
| RNU6-129P | GC10P083764 | GENECARDS |
| ENSG00000237653 | GC03M191952 | GENECARDS |
| ENSG00000237669 | GC06M030910 | GENECARDS |
| ENSG00000238694 | GC04M034967 | GENECARDS |
| ENSG00000201311 | GC02M082307 | GENECARDS |
| piR-45783 | GC07P158811 | GENECARDS |
| lnc-NANS-4 | GC09P098255 | GENECARDS |
| ENSG00000230521 | GC06M030856 | GENECARDS |
| JA662191 | GC20P046009 | GENECARDS |
| lnc-PGPEP1L-16 | GC15M104009 | GENECARDS |
| lnc-PGPEP1L-18 | GC15M104011 | GENECARDS |
| piR-47086-092 | GC12P070765 | GENECARDS |
| CARS1P2 | GC08M114794 | GENECARDS |
| ABCF1-DT | GC06M030908 | GENECARDS |
| lnc-AUNIP-6 | GC01M025692 | GENECARDS |
| MN309174-118 | GC12M070867 | GENECARDS |
| LOC105374780 | GC02M065439 | GENECARDS |
| piR-38005-040 | GC03M069920 | GENECARDS |
| RF00001-007 | GC01M078095 | GENECARDS |
| RF00001-052 | GC10M126146 | GENECARDS |
| piR-40476-044 | GC03M034097 | GENECARDS |
| piR-32662 | GC12M055052 | GENECARDS |
| ENSG00000286370 | GC02P117536 | GENECARDS |
| piR-32023-108 | GC02M070799 | GENECARDS |
| MF281430-014 | GC12M106912 | GENECARDS |
| lnc-RIC8B-1 | GC12P106911 | GENECARDS |
| piR-59769-333 | GC02P023906 | GENECARDS |
| HSALNG0049163 | GC06M030892 | GENECARDS |
| HSALNG0067880 | GC08P115073 | GENECARDS |
| LOC105378000 | GC06M130120 | GENECARDS |
| ENSG00000227291 | GC02M117180 | GENECARDS |
| NONHSAG043414.2 | GC06P047947 | GENECARDS |
| LOC105371005 | GC15M097106 | GENECARDS |
| piR-36588-229 | GC02M070801 | GENECARDS |
| LOC107984997 | GC01M078125 | GENECARDS |
| ENSG00000258746 | GC14M045502 | GENECARDS |
| piR-37026-125 | GC12M055098 | GENECARDS |
| ENSG00000275250 | GC04P034714 | GENECARDS |
| lnc-POLDIP3-3 | GC22M045584 | GENECARDS |
| HSALNG0022753 | GC02M227556 | GENECARDS |
| MAPK14 | GC06P047451 | GENECARDS |
| S1PR1 | GC01P101236 | GENECARDS |
| FCN3 | GC01M027379 | GENECARDS |
| IL5 | GC05M132541 | GENECARDS |
| CXCL8 | GC04P073740 | GENECARDS |
| TP53 | GC17M007661 | GENECARDS |
| KLK5 | GC19M050943 | GENECARDS |
| CXCR3 | GC0XM071615 | GENECARDS |
| CCL5 | GC17M035871 | GENECARDS |
| DHX58 | GC17M042101 | GENECARDS |
| PTPA | GC09P129111 | GENECARDS |
| MIR584 | GC05M149062 | GENECARDS |
| MIR1249 | GC22M045200 | GENECARDS |
| CRKL | GC22P020917 | GENECARDS |
| TLR7 | GC0XP012867 | GENECARDS |
| F2RL1 | GC05P076818 | GENECARDS |
| IL15 | GC04P141636 | GENECARDS |
| GLDC | GC09M006522 | GENECARDS |
| NCL | GC02M231453 | GENECARDS |
| IFNL3 | GC19M039243 | GENECARDS |
| MIR22 | GC17M001713 | GENECARDS |
| IFNG | GC12M068064 | GENECARDS |
| ITGA5 | GC12M054396 | GENECARDS |
| ITGB6 | GC02M160099 | GENECARDS |
| CCR1 | GC03M046218 | GENECARDS |
| TUFM | GC16M028858 | GENECARDS |
| ERAP1 | GC05M096760 | GENECARDS |
| CLEC4M | GC19P007763 | GENECARDS |
| NLRX1 | GC11P119166 | GENECARDS |
| FCN1 | GC09M134975 | GENECARDS |
| TMPRSS11D | GC04M067820 | GENECARDS |
| MIR26A1 | GC03P037969 | GENECARDS |
| MIR335 | GC07P130496 | GENECARDS |
| MIR628 | GC15M055372 | GENECARDS |
| MIR576 | GC04P109488 | GENECARDS |
| MIR664A | GC01M220200 | GENECARDS |
| MIR1260A | GC14P077266 | GENECARDS |
| MAVS | GC20P003827 | GENECARDS |
| MMP2 | GC16P055390 | GENECARDS |
| APP | GC21M025880 | GENECARDS |
| CAMK2B | GC07M044217 | GENECARDS |
| RELA | GC11M065653 | GENECARDS |
| ATP1A1 | GC01P116372 | GENECARDS |
| UBE2I | GC16P001415 | GENECARDS |
| PDCD1 | GC02M241849 | GENECARDS |
| AQP3 | GC09M033431 | GENECARDS |
| FGF2 | GC04P122826 | GENECARDS |
| SCNN1A | GC12M006346 | GENECARDS |
| PTK2 | GC08M140657 | GENECARDS |
| RHOA | GC03M049359 | GENECARDS |
| SUMO1 | GC02M202206 | GENECARDS |
| FPR2 | GC19P051752 | GENECARDS |
| CCR7 | GC17M040556 | GENECARDS |
| AQP1 | GC07P030911 | GENECARDS |
| AQP5 | GC12P049961 | GENECARDS |
| CD27 | GC12P008144 | GENECARDS |
| IKBKE | GC01P206470 | GENECARDS |
| HAMP | GC19P038216 | GENECARDS |
| CEACAM1 | GC19M042507 | GENECARDS |
| DUOX2 | GC15M045092 | GENECARDS |
| IL9 | GC05M135891 | GENECARDS |
| IL17A | GC06P052186 | GENECARDS |
| CAMP | GC03P048266 | GENECARDS |
| DUOX1 | GC15P045129 | GENECARDS |
| IFNA2 | GC09M021384 | GENECARDS |
| CCL4 | GC17P036103 | GENECARDS |
| CXCL11 | GC04M076033 | GENECARDS |
| TLR10 | GC04M038773 | GENECARDS |
| IL32 | GC16P004242 | GENECARDS |
| CPSF1 | GC08M144393 | GENECARDS |
| BTLA | GC03M112463 | GENECARDS |
| HERC5 | GC04P088457 | GENECARDS |
| CUEDC2 | GC10M102424 | GENECARDS |
| CPSF3 | GC02P009423 | GENECARDS |
| FYB1 | GC05M039105 | GENECARDS |
| IGLL5 | GC22P024570 | GENECARDS |
| MIR155 | GC21P025573 | GENECARDS |
| MIR324 | GC17M007223 | GENECARDS |
| SPHK1 | GC17P076376 | GENECARDS |
| TRAF6 | GC11M036467 | GENECARDS |
| TLR8 | GC0XP012924 | GENECARDS |
| CDC25B | GC20P003787 | GENECARDS |
| IL12A | GC03P159988 | GENECARDS |
| ANP32A | GC15M068778 | GENECARDS |
| BST2 | GC19M017403 | GENECARDS |
| EBI3 | GC19P004232 | GENECARDS |
| IFNL1 | GC19P039296 | GENECARDS |
| RTRAF | GC14P051992 | GENECARDS |
| PRKCD | GC03P053156 | GENECARDS |
| RPS6KA3 | GC0XM020149 | GENECARDS |
| NFKB1 | GC04P102501 | GENECARDS |
| HDAC6 | GC0XP048801 | GENECARDS |
| CDC42 | GC01P022057 | GENECARDS |
| PCNA | GC20M005114 | GENECARDS |
| SRC | GC20P037344 | GENECARDS |
| PIK3R1 | GC05P068215 | GENECARDS |
| TLR2 | GC04P153684 | GENECARDS |
| IL2RA | GC10M006010 | GENECARDS |
| IFNGR1 | GC06M137197 | GENECARDS |
| GLUL | GC01M182350 | GENECARDS |
| MIF | GC22P023894 | GENECARDS |
| AIFM1 | GC0XM130129 | GENECARDS |
| RIPK1 | GC06P003064 | GENECARDS |
| CSNK1A1 | GC05M149492 | GENECARDS |
| NOS2 | GC17M027756 | GENECARDS |
| PTGS2 | GC01M186640 | GENECARDS |
| TUBA1A | GC12M049184 | GENECARDS |
| IRF5 | GC07P128937 | GENECARDS |
| FADD | GC11P070203 | GENECARDS |
| PIK3CB | GC03M138652 | GENECARDS |
| TLR1 | GC04M038797 | GENECARDS |
| TLR5 | GC01M223109 | GENECARDS |
| IRF1 | GC05M132481 | GENECARDS |
| BAD | GC11M064273 | GENECARDS |
| DDX5 | GC17M064498 | GENECARDS |
| TNFSF10 | GC03M172505 | GENECARDS |
| RAB11A | GC15P072880 | GENECARDS |
| EZR | GC06M158765 | GENECARDS |
| AICDA | GC12M008602 | GENECARDS |
| ERN1 | GC17M064039 | GENECARDS |
| C1QA | GC01P022636 | GENECARDS |
| CRK | GC17M001420 | GENECARDS |
| HNRNPA2B1 | GC07M026174 | GENECARDS |
| IFNAR1 | GC21P033324 | GENECARDS |
| NUP62 | GC19M049906 | GENECARDS |
| TNFSF13B | GC13P108251 | GENECARDS |
| TBX21 | GC17P047733 | GENECARDS |
| TLR9 | GC03M052222 | GENECARDS |
| IL2 | GC04M122451 | GENECARDS |
| MYO6 | GC06P075749 | GENECARDS |
| XBP1 | GC22M028794 | GENECARDS |
| ADAR | GC01M154582 | GENECARDS |
| CSF2 | GC05P132073 | GENECARDS |
| SGPL1 | GC10P070815 | GENECARDS |
| POLR2A | GC17P008025 | GENECARDS |
| KLF6 | GC10M003779 | GENECARDS |
| TLR6 | GC04M038828 | GENECARDS |
| IL3 | GC05P132060 | GENECARDS |
| GHRL | GC03M010285 | GENECARDS |
| RIPK3 | GC14M024336 | GENECARDS |
| CD244 | GC01M160830 | GENECARDS |
| CD70 | GC19M006583 | GENECARDS |
| IL18R1 | GC02P102311 | GENECARDS |
| PRSS3 | GC09P033750 | GENECARDS |
| HSPA1A | GC06P047326 | GENECARDS |
| VAPA | GC18P009904 | GENECARDS |
| GNB2 | GC07P100673 | GENECARDS |
| EEF1D | GC08M143579 | GENECARDS |
| KPNA1 | GC03M122421 | GENECARDS |
| APOBEC3G | GC22P039078 | GENECARDS |
| MLKL | GC16M074672 | GENECARDS |
| SFTPA1 | GC10P084171 | GENECARDS |
| PCBP1 | GC02P070087 | GENECARDS |
| NFKBIB | GC19P038899 | GENECARDS |
| IRAK2 | GC03P010210 | GENECARDS |
| KHSRP | GC19M006413 | GENECARDS |
| PTX3 | GC03P157436 | GENECARDS |
| TMPRSS15 | GC21M018269 | GENECARDS |
| CPSF4 | GC07P099438 | GENECARDS |
| HNRNPF | GC10M043385 | GENECARDS |
| DHX9 | GC01P182839 | GENECARDS |
| DEFA1 | GC08M006977 | GENECARDS |
| TOMM40 | GC19P044890 | GENECARDS |
| TAF6 | GC07M100107 | GENECARDS |
| TMPRSS4 | GC11P118077 | GENECARDS |
| METTL3 | GC14M021498 | GENECARDS |
| CXCL9 | GC04M076001 | GENECARDS |
| COX6C | GC08M099899 | GENECARDS |
| DR1 | GC01P093345 | GENECARDS |
| ARHGAP21 | GC10M024534 | GENECARDS |
| CMAS | GC12P022046 | GENECARDS |
| DEFA5 | GC08M007057 | GENECARDS |
| RNF128 | GC0XP106693 | GENECARDS |
| IL27 | GC16M028511 | GENECARDS |
| ILF3 | GC19P010625 | GENECARDS |
| YTHDF2 | GC01P028751 | GENECARDS |
| APOBEC3F | GC22P039024 | GENECARDS |
| DEFA6 | GC08M006924 | GENECARDS |
| NXT1 | GC20P023350 | GENECARDS |
| TRAPPC6A | GC19M045162 | GENECARDS |
| RACK1 | GC05M181310 | GENECARDS |
| MORC3 | GC21P036320 | GENECARDS |
| DEFA4 | GC08M006935 | GENECARDS |
| MOAP1 | GC14M093182 | GENECARDS |
| NUP54 | GC04M076114 | GENECARDS |
| KLK12 | GC19M051029 | GENECARDS |
| IFNL2 | GC19P039268 | GENECARDS |
| LSM14A | GC19P034172 | GENECARDS |
| DNAJB13 | GC11P073950 | GENECARDS |
| RRP1B | GC21P043659 | GENECARDS |
| TRIM41 | GC05P181222 | GENECARDS |
| CLEC5A | GC07M141927 | GENECARDS |
| DPH7 | GC09M137554 | GENECARDS |
| TMEM181 | GC06P158536 | GENECARDS |
| MIR29A | GC07M130876 | GENECARDS |
| MIR302A | GC04M112679 | GENECARDS |
| MIR203A | GC14P104655 | GENECARDS |
| MIR4776-1 | GC02P212926 | GENECARDS |
| MIR4776-2 | GC02M212926 | GENECARDS |
| MIR4276 | GC04P174423 | GENECARDS |
| TLR4 | GC09P117704 | GENECARDS |
| IL22RA1 | GC01M024119 | GENECARDS |
| SOD1 | GC21P031659 | GENECARDS |
| ALB | GC04P073397 | GENECARDS |
| HCRTR2 | GC06P055106 | GENECARDS |
| VNN1 | GC06M132680 | GENECARDS |
| HOXD13 | GC02P176092 | GENECARDS |
| MIR141 | GC12P008231 | GENECARDS |
| EGFR | GC07P055019 | GENECARDS |
| SOD2 | GC06M159669 | GENECARDS |
| TBK1 | GC12P064451 | GENECARDS |
| FYN | GC06M111660 | GENECARDS |
| INS | GC11M002159 | GENECARDS |
| ACTN4 | GC19P038647 | GENECARDS |
| FURIN | GC15P090868 | GENECARDS |
| APPL1 | GC03P057227 | GENECARDS |
| PRSS1 | GC07P144938 | GENECARDS |
| ST14 | GC11P130159 | GENECARDS |
| AGER | GC06M032180 | GENECARDS |
| IL18 | GC11M112143 | GENECARDS |
| KNG1 | GC03P186717 | GENECARDS |
| HAVCR2 | GC05M157063 | GENECARDS |
| TRIM28 | GC19P058544 | GENECARDS |
| POLR1C | GC06P047535 | GENECARDS |
| PF4 | GC04M073980 | GENECARDS |
| SOCS5 | GC02P046698 | GENECARDS |
| ISG20 | GC15P088635 | GENECARDS |
| KLF2 | GC19P023268 | GENECARDS |
| SPN | GC16P029662 | GENECARDS |
| BANP | GC16P087949 | GENECARDS |
| ADPRH | GC03P119579 | GENECARDS |
| CLEC4A | GC12P008267 | GENECARDS |
| TMPRSS13 | GC11M117900 | GENECARDS |
| IFITM5 | GC11M000298 | GENECARDS |
| TMPRSS11E | GC04P068447 | GENECARDS |
| GYPE | GC04M143870 | GENECARDS |
| LINC01191 | GC02P115314 | GENECARDS |
| PSMB8-AS1 | GC06P032844 | GENECARDS |
| SCGB1C1 | ENSG00000188076,ENST00000342878.3 | OMIM |
| ODF3 | ENSG00000177947,ENST00000525282.1 | OMIM |
| BET1L | ENSG00000177951,ENST00000382762.8 | OMIM |
| RIC8A | ENSG00000177963,ENST00000526104.6 | OMIM |
| SIRT3 | ENSG00000142082,ENST00000524564.5 | OMIM |
| PSMD13 | ENSG00000185627,ENST00000532097.6 | OMIM |
| NLRP6 | ENSG00000174885,ENST00000534750.6 | OMIM |
| PGGHG | ENSG00000142102,ENST00000409548.7 | OMIM |
| B4GALNT4 | ENSG00000182272,ENST00000329962.11 | OMIM |
| PKP3 | ENSG00000184363,ENST00000331563.7 | OMIM |
| SIGIRR | ENSG00000185187,ENST00000397632.7 | OMIM |
| PTDSS2 | ENSG00000174915,ENST00000308020.6 | OMIM |
| RNH1 | ENSG00000023191,ENST00000397615.6 | OMIM |
| LRRC56 | ENSG00000161328,ENST00000270115.8 | OMIM |
| HRAS | ENSG00000174775,ENST00000451590.5 | OMIM |
| RASSF7 | ENSG00000099849,ENST00000397582.7 | OMIM |
| MIR210 | ENSG00000199038,ENST00000362168.1 | OMIM |
| PHRF1 | ENSG00000070047,ENST00000264555.10 | OMIM |
| CDHR5 | ENSG00000099834,ENST00000358353.8 | OMIM |
| SCT | ENSG00000070031,ENST00000176195.4 | OMIM |
| DRD4 | ENSG00000069696,ENST00000176183.6 | OMIM |
| DEAF1 | ENSG00000177030,ENST00000683307.1 | OMIM |
| EPS8L2 | ENSG00000177106,ENST00000318562.13 | OMIM |
| TALDO1 | ENSG00000177156,ENST00000319006.8 | OMIM |
| CEND1 | ENSG00000184524,ENST00000330106.5 | OMIM |
| SLC25A22 | ENSG00000177542,ENST00000531214.5 | OMIM |
| PIDD1 | ENSG00000177595,ENST00000347755.10 | OMIM |
| RPLP2 | ENSG00000177600,ENST00000321153.9 | OMIM |
| PNPLA2 | ENSG00000177666,ENST00000336615.9 | OMIM |
| CRACR2B | ENSG00000177685,ENST00000525077.2 | OMIM |
| CD151 | ENSG00000177697,ENST00000397420.9 | OMIM |
| TSPAN4 | ENSG00000214063,ENST00000397406.5 | OMIM |
| CHID1 | ENSG00000177830,ENST00000436108.6 | OMIM |
| AP2A2 | ENSG00000183020,ENST00000332231.9 | OMIM |
| MUC6 | ENSG00000184956,ENST00000421673.7 | OMIM |
| MUC2 | ENSG00000198788,ENST00000361558.7 | OMIM |
| MUC5AC | ENSG00000215182,ENST00000621226.2 | OMIM |
| MUC5B | ENSG00000117983,ENST00000529681.5 | OMIM |
| TOLLIP | ENSG00000078902,ENST00000530541.1 | OMIM |
| BRSK2 | ENSG00000174672,ENST00000528841.6 | OMIM |
| MOB2 | ENSG00000182208,ENST00000329957.7 | OMIM |
| DUSP8 | ENSG00000184545,ENST00000397374.8 | OMIM |
| KRTAP5-1 | ENSG00000205869,ENST00000382171.2 | OMIM |
| IFITM10 | ENSG00000244242,ENST00000340134.5 | OMIM |
| CTSD | ENSG00000117984,ENST00000236671.7 | OMIM |
| SYT8 | ENSG00000149043,ENST00000381978.7 | OMIM |
| TNNI2 | ENSG00000130598,ENST00000381906.5 | OMIM |
| LSP1 | ENSG00000130592,ENST00000405957.6 | OMIM |
| TNNT3 | ENSG00000130595,ENST00000381579.7 | OMIM |
| MRPL23 | ENSG00000214026,ENST00000397298.8 | OMIM |
| H19 | ENSG00000130600,ENST00000414790.7 | OMIM |
| MIR675 | ENSG00000284010,ENST00000390168.6 | OMIM |

**Supplementary Table S4：**

By employing the available TCMSP databases and wide-scale searches of the literature, including PubMed and China National Knowledge Infrastructure (CNKI) databases, we obtained 245 CSTRP related targets supplementary Table S4

| **Entry** | **Entry name** | **Protein names** | **Gene name** |
| --- | --- | --- | --- |
| P43115 | PE2R3_HUMAN | Prostaglandin E2 receptor EP3 subtype | PTGER3 |
| P11511 | CP19A_HUMAN | Aromatase | CYP19A1 |
| P11802 | CDK4_HUMAN | Cyclin-dependent kinase 4 | CDK4 |
| P45844 | ABCG1_HUMAN | ATP-binding cassette sub-family G member 1 | ABCG1 |
| P11021 | BIP_HUMAN | Endoplasmic reticulum chaperone BiP | HSPA5 |
| Q01469 | FABP5_HUMAN | Fatty acid-binding protein 5 | FABP5 |
| P01857 | IGHG1_HUMAN | Immunoglobulin heavy constant gamma 1 | IGHG1 |
| P29474 | NOS3_HUMAN | Nitric oxide synthase, endothelial | NOS3 |
| O43451 | MGA_HUMAN | Maltase-glucoamylase, intestinal [Includes: Maltase | MGAM |
| P80108 | PHLD_HUMAN | Phosphatidylinositol-glycan-specific phospholipase D | GPLD1 |
| P01344 | IGF2_HUMAN | Insulin-like growth factor II | IGF2 |
| P10415 | BCL2_HUMAN | Apoptosis regulator Bcl-2 | BCL2 |
| O95433 | AHSA1_HUMAN | Activator of 90 kDa heat shock protein ATPase homolog 1 | AHSA1 |
| P29965 | CD40L_HUMAN | CD40 ligand | CD40LG |
| P28340 | DPOD1_HUMAN | DNA polymerase delta catalytic subunit | POLD1 |
| Q92819 | HYAS2_HUMAN | Hyaluronan synthase 2 | HAS2 |
| P28482 | MK01_HUMAN | Mitogen-activated protein kinase 1 | MAPK1 |
| P09848 | LPH_HUMAN | Lactase-phlorizin hydrolase | LCT |
| O14939 | PLD2_HUMAN | Phospholipase D2 | PLD2 |
| Q04206 | TF65_HUMAN | Transcription factor p65 | RELA |
| P05771 | KPCB_HUMAN | Protein kinase C beta type | PRKCB |
| P00441 | SODC_HUMAN | Superoxide dismutase [Cu-Zn] | SOD1 |
| Q13393 | PLD1_HUMAN | Phospholipase D1 | PLD1 |
| P60484 | PTEN_HUMAN | Phosphatidylinositol 3,4,5-trisphosphate 3-phosphatase and dual-specificity protein phosphatase PTEN | PTEN |
| Q8IV08 | PLD3_HUMAN | 5'-3' exonuclease PLD3 | PLD3 |
| P01106 | MYC_HUMAN | Myc proto-oncogene protein | MYC |
| P15309 | PPAP_HUMAN | Prostatic acid phosphatase | ACP3 |
| P08254 | MMP3_HUMAN | Stromelysin-1 | MMP3 |
| P06400 | RB_HUMAN | Retinoblastoma-associated protein | RB1 |
| P49585 | PCY1A_HUMAN | Choline-phosphate cytidylyltransferase A | PCYT1A |
| P08294 | SODE_HUMAN | Extracellular superoxide dismutase [Cu-Zn] | SOD3 |
| Q8IWA5 | CTL2_HUMAN | Choline transporter-like protein 2 | SLC44A2 |
| P42574 | CASP3_HUMAN | Caspase-3 | CASP3 |
| P04798 | CP1A1_HUMAN | Cytochrome P450 1A1 | CYP1A1 |
| P11836 | CD20_HUMAN | B-lymphocyte antigen CD20 | MS4A1 |
| P55211 | CASP9_HUMAN | Caspase-9 | CASP9 |
| P24385 | CCND1_HUMAN | G1/S-specific cyclin-D1 | CCND1 |
| Q01094 | E2F1_HUMAN | Transcription factor E2F1 | E2F1 |
| P01584 | IL1B_HUMAN | Interleukin-1 beta | IL1B |
| P60709 | ACTB_HUMAN | Actin, cytoplasmic 1 | ACTB |
| P04040 | CATA_HUMAN | Catalase | CAT |
| P09917 | LOX5_HUMAN | Polyunsaturated fatty acid 5-lipoxygenase | ALOX5 |
| Q9UII4 | HERC5_HUMAN | E3 ISG15--protein ligase HERC5 | HERC5 |
| P27361 | MK03_HUMAN | Mitogen-activated protein kinase 3 | MAPK3 |
| P49895 | IOD1_HUMAN | Type I iodothyronine deiodinase | DIO1 |
| Q8IVI9 | NOSTN_HUMAN | Nostrin | NOSTRIN |
| P05231 | IL6_HUMAN | Interleukin-6 | IL6 |
| P05412 | JUN_HUMAN | Transcription factor AP-1 | JUN |
| Q9BYJ1 | LOXE3_HUMAN | Hydroperoxide isomerase ALOXE3 | ALOXE3 |
| P01375 | TNFA_HUMAN | Tumor necrosis factor | TNF |
| P15692 | VEGFA_HUMAN | Vascular endothelial growth factor A | VEGFA |
| O43242 | PSMD3_HUMAN | 26S proteasome non-ATPase regulatory subunit 3 | PSMD3 |
| P04049 | RAF1_HUMAN | RAF proto-oncogene serine/threonine-protein kinase | RAF1 |
| P19793 | RXRA_HUMAN | Retinoic acid receptor RXR-alpha | RXRA |
| Q9NS23 | RASF1_HUMAN | Ras association domain-containing protein 1 | RASSF1 |
| P23975 | SC6A2_HUMAN | Sodium-dependent noradrenaline transporter | SLC6A2 |
| Q13950 | RUNX2_HUMAN | Runt-related transcription factor 2 | RUNX2 |
| Q14524 | SCN5A_HUMAN | Sodium channel protein type 5 subunit alpha | SCN5A |
| P27169 | PON1_HUMAN | Serum paraoxonase/arylesterase 1 | PON1 |
| P11137 | MTAP2_HUMAN | Microtubule-associated protein 2 | MAP2 |
| P03973 | SLPI_HUMAN | Antileukoproteinase | SLPI |
| P78380 | OLR1_HUMAN | Oxidized low-density lipoprotein receptor 1 | OLR1 |
| P05164 | PERM_HUMAN | Myeloperoxidase | MPO |
| Q01959 | SC6A3_HUMAN | Sodium-dependent dopamine transporter | SLC6A3 |
| P04179 | SODM_HUMAN | Superoxide dismutase [Mn], mitochondrial | SOD2 |
| P28161 | GSTM2_HUMAN | Glutathione S-transferase Mu 2 | GSTM2 |
| Q07869 | PPARA_HUMAN | Peroxisome proliferator-activated receptor alpha | PPARA |
| P47712 | PA24A_HUMAN | Cytosolic phospholipase A2 | PLA2G4A |
| P14780 | MMP9_HUMAN | Matrix metalloproteinase-9 | MMP9 |
| Q99801 | NKX31_HUMAN | Homeobox protein Nkx-3.1 | NKX3-1 |
| P41143 | OPRD_HUMAN | Delta-type opioid receptor | OPRD1 |
| P49841 | GSK3B_HUMAN | Glycogen synthase kinase-3 beta | GSK3B |
| Q14432 | PDE3A_HUMAN | cGMP-inhibited 3',5'-cyclic phosphodiesterase A | PDE3A |
| Q14994 | NR1I3_HUMAN | Nuclear receptor subfamily 1 group I member 3 | NR1I3 |
| P15941 | MUC1_HUMAN | Mucin-1 | MUC1 |
| P35228 | NOS2_HUMAN | Nitric oxide synthase, inducible | NOS2 |
| Q06455 | MTG8_HUMAN | Protein CBFA2T1 | RUNX1T1 |
| P05121 | PAI1_HUMAN | Plasminogen activator inhibitor 1 | SERPINE1 |
| P55786 | PSA_HUMAN | Puromycin-sensitive aminopeptidase | NPEPPS |
| P20936 | RASA1_HUMAN | Ras GTPase-activating protein 1 | RASA1 |
| Q96PH1 | NOX5_HUMAN | NADPH oxidase 5 | NOX5 |
| P23219 | PGH1_HUMAN | Prostaglandin G/H synthase 1 | PTGS1 |
| Q05655 | KPCD_HUMAN | Protein kinase C delta type | PRKCD |
| P11387 | TOP1_HUMAN | DNA topoisomerase 1 | TOP1 |
| P55072 | TERA_HUMAN | Transitional endoplasmic reticulum ATPase | VCP |
| P19320 | VCAM1_HUMAN | Vascular cell adhesion protein 1 | VCAM1 |
| O14684 | PTGES_HUMAN | Prostaglandin E synthase | PTGES |
| P18031 | PTN1_HUMAN | Tyrosine-protein phosphatase non-receptor type 1 | PTPN1 |
| P06401 | PRGR_HUMAN | Progesterone receptor | PGR |
| Q00613 | HSF1_HUMAN | Heat shock factor protein 1 | HSF1 |
| P12004 | PCNA_HUMAN | Proliferating cell nuclear antigen | PCNA |
| O75469 | NR1I2_HUMAN | Nuclear receptor subfamily 1 group I member 2 | NR1I2 |
| Q15788 | NCOA1_HUMAN | Nuclear receptor coactivator 1 | NCOA1 |
| P17612 | KAPCA_HUMAN | cAMP-dependent protein kinase catalytic subunit alpha | PRKACA |
| Q13162 | PRDX4_HUMAN | Peroxiredoxin-4 | PRDX4 |
| P13726 | TF_HUMAN | Tissue factor | F3 |
| Q99973 | TEP1_HUMAN | Telomerase protein component 1 | TEP1 |
| P11388 | TOP2A_HUMAN | DNA topoisomerase 2-alpha | TOP2A |
| Q9Y233 | PDE10_HUMAN | cAMP and cAMP-inhibited cGMP 3',5'-cyclic phosphodiesterase 10A | PDE10A |
| P09874 | PARP1_HUMAN | Poly [ADP-ribose] polymerase 1 | PARP1 |
| P04637 | P53_HUMAN | Cellular tumor antigen p53 | TP53 |
| P00750 | TPA_HUMAN | Tissue-type plasminogen activator | PLAT |
| P31645 | SC6A4_HUMAN | Sodium-dependent serotonin transporter | SLC6A4 |
| P42224 | STAT1_HUMAN | Signal transducer and activator of transcription 1-alpha/beta | STAT1 |
| P11217 | PYGM_HUMAN | Glycogen phosphorylase, muscle form | PYGM |
| P08912 | ACM5_HUMAN | Muscarinic acetylcholine receptor M5 | CHRM5 |
| P10275 | ANDR_HUMAN | Androgen receptor | AR |
| O14625 | CXL11_HUMAN | C-X-C motif chemokine 11 | CXCL11 |
| Q13255 | GRM1_HUMAN | Metabotropic glutamate receptor 1 | GRM1 |
| P07339 | CATD_HUMAN | Cathepsin D | CTSD |
| P09488 | GSTM1_HUMAN | Glutathione S-transferase Mu 1 | GSTM1 |
| P08684 | CP3A4_HUMAN | Cytochrome P450 3A4 | CYP3A4 |
| P00742 | FA10_HUMAN | Coagulation factor X | F10 |
| P14635 | CCNB1_HUMAN | G2/mitotic-specific cyclin-B1 | CCNB1 |
| P20813 | CP2B6_HUMAN | Cytochrome P450 2B6 | CYP2B6 |
| P99999 | CYC_HUMAN | Cytochrome c | CYCS |
| P02778 | CXL10_HUMAN | C-X-C motif chemokine 10 | CXCL10 |
| P35368 | ADA1B_HUMAN | Alpha-1B adrenergic receptor | ADRA1B |
| P55210 | CASP7_HUMAN | Caspase-7 | CASP7 |
| P52789 | HXK2_HUMAN | Hexokinase-2 | HK2 |
| P10145 | IL8_HUMAN | Interleukin-8 | CXCL8 |
| P25963 | IKBA_HUMAN | NF-kappa-B inhibitor alpha | NFKBIA |
| O15111 | IKKA_HUMAN | Inhibitor of nuclear factor kappa-B kinase subunit alpha | CHUK |
| O14920 | IKKB_HUMAN | Inhibitor of nuclear factor kappa-B kinase subunit beta | IKBKB |
| P08235 | MCR_HUMAN | Mineralocorticoid receptor | NR3C2 |
| P16581 | LYAM2_HUMAN | E-selectin | SELE |
| P08034 | CXB1_HUMAN | Gap junction beta-1 protein | GJB1 |
| P49327 | FAS_HUMAN | Fatty acid synthase | FASN |
| P01148 | GON1_HUMAN | Progonadoliberin-1 | GNRH1 |
| Q13873 | BMPR2_HUMAN | Bone morphogenetic protein receptor type-2 | BMPR2 |
| P08173 | ACM4_HUMAN | Muscarinic acetylcholine receptor M4 | CHRM4 |
| P27487 | DPP4_HUMAN | Dipeptidyl peptidase 4 | DPP4 |
| Q9NRD8 | DUOX2_HUMAN | Dual oxidase 2 | DUOX2 |
| P20309 | ACM3_HUMAN | Muscarinic acetylcholine receptor M3 | CHRM3 |
| Q15822 | ACHA2_HUMAN | Neuronal acetylcholine receptor subunit alpha-2 | CHRNA2 |
| P11229 | ACM1_HUMAN | Muscarinic acetylcholine receptor M1 | CHRM1 |
| P25100 | ADA1D_HUMAN | Alpha-1D adrenergic receptor | ADRA1D |
| Q03135 | CAV1_HUMAN | Caveolin-1 | CAV1 |
| P35222 | CTNB1_HUMAN | Catenin beta-1 | CTNNB1 |
| P27338 | AOFB_HUMAN | Amine oxidase [flavin-containing] B | MAOB |
| P08588 | ADRB1_HUMAN | Beta-1 adrenergic receptor | ADRB1 |
| O15392 | BIRC5_HUMAN | Baculoviral IAP repeat-containing protein 5 | BIRC5 |
| O14757 | CHK1_HUMAN | Serine/threonine-protein kinase Chk1 | CHEK1 |
| P38936 | CDN1A_HUMAN | Cyclin-dependent kinase inhibitor 1 | CDKN1A |
| P35348 | ADA1A_HUMAN | Alpha-1A adrenergic receptor | ADRA1A |
| Q16678 | CP1B1_HUMAN | Cytochrome P450 1B1 | CYP1B1 |
| P08913 | ADA2A_HUMAN | Alpha-2A adrenergic receptor | ADRA2A |
| P56817 | BACE1_HUMAN | Beta-secretase 1 | BACE1 |
| P06850 | CRF_HUMAN | Corticoliberin | CRH |
| P17302 | CXA1_HUMAN | Gap junction alpha-1 protein | GJA1 |
| P11926 | DCOR_HUMAN | Ornithine decarboxylase | ODC1 |
| P31749 | AKT1_HUMAN | RAC-alpha serine/threonine-protein kinase | AKT1 |
| P54289 | CA2D1_HUMAN | Voltage-dependent calcium channel subunit alpha-2/delta-1 | CACNA2D1 |
| P36544 | ACHA7_HUMAN | Neuronal acetylcholine receptor subunit alpha-7 | CHRNA7 |
| P22303 | ACES_HUMAN | Acetylcholinesterase | ACHE |
| P11712 | CP2C9_HUMAN | Cytochrome P450 2C9 | CYP2C9 |
| Q96JK2 | DCAF5_HUMAN | DDB1- and CUL4-associated factor 5 | DCAF5 |
| P21397 | AOFA_HUMAN | Amine oxidase [flavin-containing] A | MAOA |
| P00918 | CAH2_HUMAN | Carbonic anhydrase 2 | CA2 |
| Q13085 | ACACA_HUMAN | Acetyl-CoA carboxylase 1 | ACACA |
| P08172 | ACM2_HUMAN | Muscarinic acetylcholine receptor M2 | CHRM2 |
| O14493 | CLD4_HUMAN | Claudin-4 | CLDN4 |
| Q07812 | BAX_HUMAN | Apoptosis regulator BAX | BAX |
| O96017 | CHK2_HUMAN | Serine/threonine-protein kinase Chk2 | CHEK2 |
| P41594 | GRM5_HUMAN | Metabotropic glutamate receptor 5 | GRM5 |
| P06213 | INSR_HUMAN | Insulin receptor | INSR |
| O75840 | KLF7_HUMAN | Krueppel-like factor 7 | KLF7 |
| P04626 | ERBB2_HUMAN | Receptor tyrosine-protein kinase erbB-2 | ERBB2 |
| P08709 | FA7_HUMAN | Coagulation factor VII | F7 |
| P15407 | FOSL1_HUMAN | Fos-related antigen 1 | FOSL1 |
| P60568 | IL2_HUMAN | Interleukin-2 | IL2 |
| P61925 | IPKA_HUMAN | cAMP-dependent protein kinase inhibitor alpha | PKIA |
| P03372 | ESR1_HUMAN | Estrogen receptor | ESR1 |
| P56537 | IF6_HUMAN | Eukaryotic translation initiation factor 6 | EIF6 |
| P19419 | ELK1_HUMAN | ETS domain-containing protein Elk-1 | ELK1 |
| Q08462 | ADCY2_HUMAN | Adenylate cyclase type 2 | ADCY2 |
| P20248 | CCNA2_HUMAN | Cyclin-A2 | CCNA2 |
| P01100 | FOS_HUMAN | Proto-oncogene c-Fos | FOS |
| P21860 | ERBB3_HUMAN | Receptor tyrosine-protein kinase erbB-3 | ERBB3 |
| P42330 | AK1C3_HUMAN | Aldo-keto reductase family 1 member C3 | AKR1C3 |
| P35869 | AHR_HUMAN | Aryl hydrocarbon receptor | AHR |
| P05090 | APOD_HUMAN | Apolipoprotein D | APOD |
| Q14790 | CASP8_HUMAN | Caspase-8 | CASP8 |
| P00533 | EGFR_HUMAN | Epidermal growth factor receptor | EGFR |
| Q92731 | ESR2_HUMAN | Estrogen receptor beta | ESR2 |
| P01583 | IL1A_HUMAN | Interleukin-1 alpha | IL1A |
| P03956 | MMP1_HUMAN | Interstitial collagenase | MMP1 |
| P17538 | CTRB1_HUMAN | Chymotrypsinogen B | CTRB1 |
| Q16539 | MK14_HUMAN | Mitogen-activated protein kinase 14 | MAPK14 |
| P42262 | GRIA2_HUMAN | Glutamate receptor 2 | GRIA2 |
| P45983 | MK08_HUMAN | Mitogen-activated protein kinase 8 | MAPK8 |
| P17252 | KPCA_HUMAN | Protein kinase C alpha type | PRKCA |
| Q9GZT9 | EGLN1_HUMAN | Egl nine homolog 1 | EGLN1 |
| P02751 | FINC_HUMAN | Fibronectin | FN1 |
| P30968 | GNRHR_HUMAN | Gonadotropin-releasing hormone receptor | GNRHR |
| Q02156 | KPCE_HUMAN | Protein kinase C epsilon type | PRKCE |
| Q12809 | KCNH2_HUMAN | Potassium voltage-gated channel subfamily H member 2 | KCNH2 |
| P01579 | IFNG_HUMAN | Interferon gamma | IFNG |
| P17936 | IBP3_HUMAN | Insulin-like growth factor-binding protein 3 | IGFBP3 |
| P10914 | IRF1_HUMAN | Interferon regulatory factor 1 | IRF1 |
| P09601 | HMOX1_HUMAN | Heme oxygenase 1 | HMOX1 |
| P05112 | IL4_HUMAN | Interleukin-4 | IL4 |
| P05362 | ICAM1_HUMAN | Intercellular adhesion molecule 1 | ICAM1 |
| P22301 | IL10_HUMAN | Interleukin-10 | IL10 |
| P08253 | MMP2_HUMAN | 72 kDa type IV collagenase | MMP2 |
| P14598 | NCF1_HUMAN | Neutrophil cytosol factor 1 | NCF1 |
| Q08209 | PP2BA_HUMAN | Serine/threonine-protein phosphatase 2B catalytic subunit alpha isoform | PPP3CA |
| P04792 | HSPB1_HUMAN | Heat shock protein beta-1 | HSPB1 |
| P07900 | HS90A_HUMAN | Heat shock protein HSP 90-alpha | HSP90AA1 |
| Q15113 | PCOC1_HUMAN | Procollagen C-endopeptidase enhancer 1 | PCOLCE |
| P15408 | FOSL2_HUMAN | Fos-related antigen 2 | FOSL2 |
| Q16236 | NF2L2_HUMAN | Nuclear factor erythroid 2-related factor 2 | NFE2L2 |
| O95644 | NFAC1_HUMAN | Nuclear factor of activated T-cells, cytoplasmic 1 | NFATC1 |
| Q9UBS5 | GABR1_HUMAN | Gamma-aminobutyric acid type B receptor subunit 1 | GABBR1 |
| P08100 | OPSD_HUMAN | Rhodopsin | RHO |
| P35372 | OPRM_HUMAN | Mu-type opioid receptor | OPRM1 |
| Q07820 | MCL1_HUMAN | Induced myeloid leukemia cell differentiation protein Mcl-1 | MCL1 |
| Q00987 | MDM2_HUMAN | E3 ubiquitin-protein ligase Mdm2 | MDM2 |
| P08581 | MET_HUMAN | Hepatocyte growth factor receptor | MET |
| Q15596 | NCOA2_HUMAN | Nuclear receptor coactivator 2 | NCOA2 |
| P42345 | MTOR_HUMAN | Serine/threonine-protein kinase mTOR | MTOR |
| Q9BZD4 | NUF2_HUMAN | Kinetochore protein Nuf2 | NUF2 |
| P09960 | LKHA4_HUMAN | Leukotriene A-4 hydrolase | LTA4H |
| Q8NHU6 | TDRD7_HUMAN | Tudor domain-containing protein 7 | TDRD7 |
| P49767 | VEGFC_HUMAN | Vascular endothelial growth factor C | VEGFC |
| P19875 | CXCL2_HUMAN | C-X-C motif chemokine 2 | CXCL2 |
| P14672 | GLUT4_HUMAN | Solute carrier family 2, facilitated glucose transporter member 4 | SLC2A4 |
| Q16665 | HIF1A_HUMAN | Hypoxia-inducible factor 1-alpha | HIF1A |
| P48023 | TNFL6_HUMAN | Tumor necrosis factor ligand superfamily member 6 | FASLG |
| Q9NUW8 | TYDP1_HUMAN | Tyrosyl-DNA phosphodiesterase 1 | TDP1 |
| P00734 | THRB_HUMAN | Prothrombin | F2 |
| P14679 | TYRO_HUMAN | Tyrosinase | TYR |
| P07477 | TRY1_HUMAN | Trypsin-1 | PRSS1 |
| P35354 | PGH2_HUMAN | Prostaglandin G/H synthase 2 | PTGS2 |
| P10451 | OSTP_HUMAN | Osteopontin | SPP1 |
| P37231 | PPARG_HUMAN | Peroxisome proliferator-activated receptor gamma | PPARG |
| Q03181 | PPARD_HUMAN | Peroxisome proliferator-activated receptor delta | PPARD |
| P07204 | TRBM_HUMAN | Thrombomodulin | THBD |
| P00749 | UROK_HUMAN | Urokinase-type plasminogen activator | PLAU |
| P35968 | VGFR2_HUMAN | Vascular endothelial growth factor receptor 2 | KDR |
| P07550 | ADRB2_HUMAN | Beta-2 adrenergic receptor | ADRB2 |
| Q07817 | B2CL1_HUMAN | Bcl-2-like protein 1 | BCL2L1 |
| Q14209 | E2F2_HUMAN | Transcription factor E2F2 | E2F2 |
| P04150 | GCR_HUMAN | Glucocorticoid receptor | NR3C1 |
