# Supplementary material for: Anti-inflammatory effects of Chaishi Tuire Granules on influenza A treatment by mediating TRAF6/MAPK14 axis
Source: Front Med (Lausanne). 2022 Nov 14;9:943681. doi: 10.3389/fmed.2022.943681 (PMC9701735; doi:10.3389/fmed.2022.943681)
Supplement: Supplementary file 1 [file Data_Sheet_1.ZIP › Raw Data/Supplementary Material/supplementary materials .pdf]

Supplementary Fig. S1:

The CCK8 assay to determine the cell viability after CSTRP drug-containing serum treatment in RAW264.7 cells.

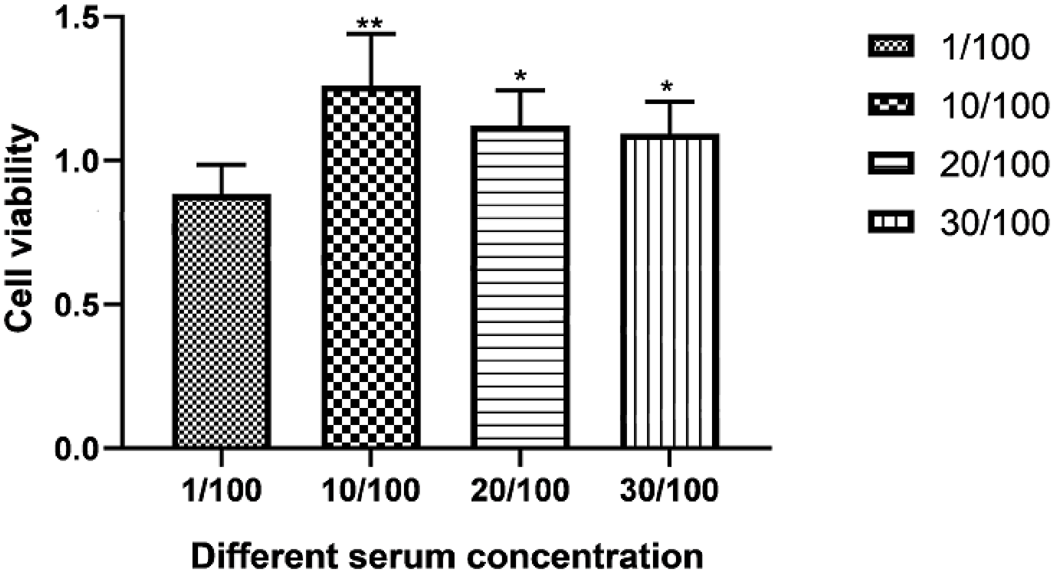

Fig. S1 The results showed that even the 10% CSTRP (5760mg/kg/day) drug-containing serum gave no cytotoxicity in RAW264.7 cells

Supplementary Fig. S2:

To explored the release of LPS-induced TNF-α on different concentrations (0, 0.5, and 1 μg/mL) and different times (8, 12, 24, and 48 h) to find out the suitable acting condition.

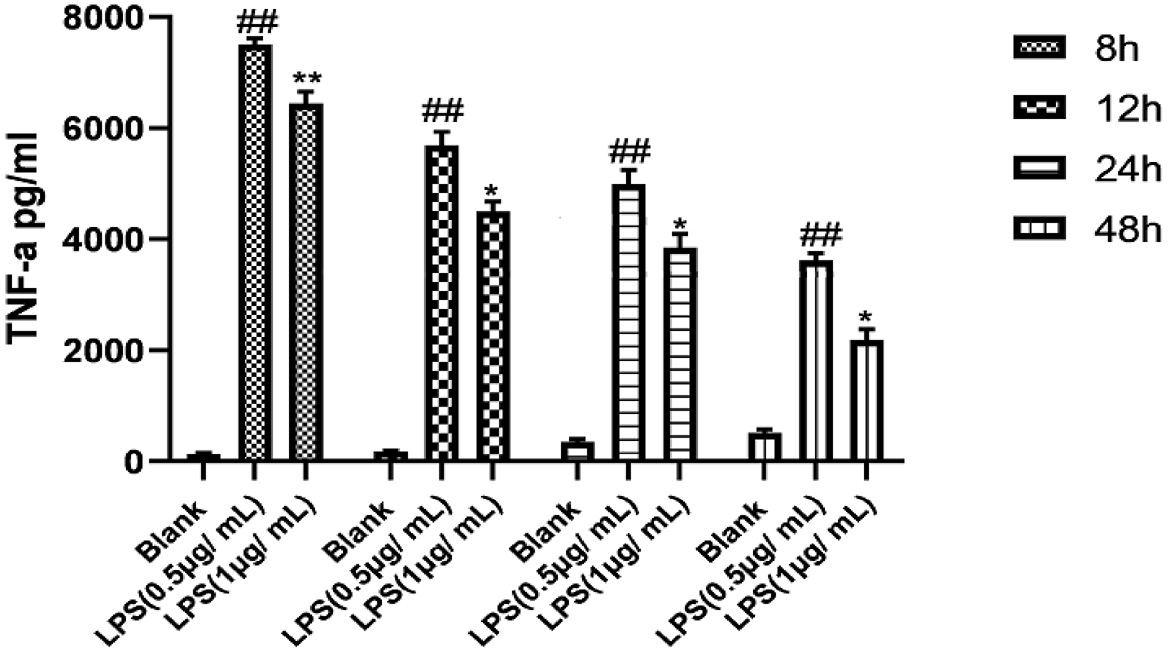

Fig. S2 0.5 μg/mL LPS treatment for 8 h was chosen for the following experiments.

Supplementary Table S1:

Primer/Probe	Sequence (5'-3')
TRAF6-F	TGCTTTGCGTCCGTGCGATG
TRAF6-R	CCGTTACACTGCTGTGCTTCCA
MAPK14-F	CTGGCTCGGCACACTGATGATG
MAPK14-R	CCTCATGGCTTGGCATCCTGTT
GAPDH-F	ATGGTGAAGGTCGGTGTGAACG
GAPDH-R	CGCTCCTGGAAGATGGTGATGG

Supplementary Table S2:

The compounds comprising the 9 kinds of Chinese medicine in CSTRP were obtained from the TCMSP database (not containing gypsum). A total of 168 candidate compounds after screening with ADME parameters (OB ≥ 30%, DL ≥ 0.14). The ultimate candidate compounds are shown in Supplementary Table S2.

TABLE S2 | 168 active compounds in CSTRP decoction related to influenza.

Mol ID	Molecule Name	MW	OB (%)	DL	Herb
MOL001689	acacetin	284.28	34.97	0.24	Scutellaria baicalensis
MOL000173	wogonin	284.28	30.68	0.23	Scutellaria baicalensis
MOL000228	(2R)-7-hydroxy-5-methoxy-2-phenylchroman-4-one	270.30	55.23	0.20	Scutellaria baicalensis
MOL002714	baicalein	270.25	33.52	0.21	Scutellaria baicalensis
MOL002908	5,8,2'-Trihydroxy-7-methoxyflavone	300.28	37.01	0.27	Scutellaria baicalensis
MOL002909	5,7,2,5-tetrahydroxy-8,6-dimethoxyflavone	376.34	33.82	0.45	Scutellaria baicalensis
MOL002910	Carthamidin	288.27	41.15	0.24	Scutellaria baicalensis
MOL002911	2,6,2',4'-tetrahydroxy-6'-methoxychaleone	302.30	69.04	0.22	Scutellaria baicalensis
MOL002913	Dihydrobaicalin_qt	272.27	40.04	0.21	Scutellaria baicalensis

MOL002914	Eriodyctiol (flavanone)	288.27	41.35	0.24	Scutellaria baicalensis
MOL002915	Salvigenin	328.34	49.07	0.33	Scutellaria baicalensis
MOL002917	5,2',6'-Trihydroxy-7,8-dimethoxyflavone	330.31	45.05	0.33	Scutellaria baicalensis
MOL002925	5,7,2',6'-Tetrahydroxyflavone	286.25	37.01	0.24	Scutellaria baicalensis
MOL002926	dihydrooroxylin A	286.30	38.72	0.23	Scutellaria baicalensis
MOL002927	Skullcapflavone II	374.37	69.51	0.44	Scutellaria baicalensis
MOL002928	oroxylin a	284.28	41.37	0.23	Scutellaria baicalensis
MOL002932	Panicolin	314.31	76.26	0.29	Scutellaria baicalensis
MOL002933	5,7,4'-Trihydroxy-8-methoxyflavone	300.28	36.56	0.27	Scutellaria baicalensis
MOL002934	NEOBAICALEIN	374.37	104.34	0.44	Scutellaria baicalensis
MOL002937	DIHYDROOROXYLIN	286.30	66.06	0.23	Scutellaria baicalensis
MOL000358	beta-sitosterol	414.79	36.91	0.75	Scutellaria baicalensis
MOL000359	sitosterol	414.79	36.91	0.75	Scutellaria baicalensis
MOL000525	Norwogonin	270.25	39.40	0.21	Scutellaria baicalensis
MOL000552	5,2'-Dihydroxy-6,7,8-trimethoxyflavone	344.34	31.71	0.35	Scutellaria baicalensis
MOL000073	ent-Epicatechin	290.29	48.96	0.24	Scutellaria baicalensis
MOL000449	Stigmasterol	412.77	43.83	0.76	Scutellaria baicalensis
MOL001458	coptisine	320.34	30.67	0.86	Scutellaria baicalensis
MOL001490	bis[(2S)-2-ethylhexyl] benzene-1,2-dicarboxylate	390.62	43.59	0.35	Scutellaria baicalensis
MOL001506	Supraene	410.80	33.55	0.42	Scutellaria baicalensis
MOL002879	Diop	390.62	43.59	0.39	Scutellaria baicalensis
MOL002897	epiberberine	336.39	43.09	0.78	Scutellaria baicalensis

MOL008206	Moslosooflavone	298.31	44.09	0.25	Scutellaria baicalensis
MOL010415	11,13-Eicosadienoic acid, methyl ester	322.59	39.28	0.23	Scutellaria baicalensis
MOL012245	5,7,4'-trihydroxy-6-methoxyflavanone	302.30	36.63	0.27	Scutellaria baicalensis
MOL012246	5,7,4'-trihydroxy-8-methoxyflavanone	302.30	74.24	0.26	Scutellaria baicalensis
MOL012266	rivularin	344.34	37.94	0.37	Scutellaria baicalensis
MOL001494	Mandenol	308.56	42.00	74.00	Lonicera japonica
MOL001495	Ethyl linolenate	306.54	46.10	75.00	Lonicera japonica
MOL002707	phytofluene	543.02	43.18	76.00	Lonicera japonica
MOL002914	Eriodyctiol (flavanone)	288.27	41.35	77.00	Lonicera japonica
MOL003006	(-)-(3R,8S,9R,9aS,10aS)-9-ethenyl-8-(beta-D-glucopyranosyloxy)-2,3,9,9a,10,10a-hexahydro-5-oxo-5H,8H-pyrano[4,3-d]oxazolo[3,2-a]pyridine-3-carboxylic acid_qt	281.29	87.47	78.00	Lonicera japonica
MOL003014	secologanic dibutylacetal_qt	384.57	53.65	0.29	Lonicera japonica
MOL002773	beta-carotene	536.96	37.18	0.58	Lonicera japonica
MOL003036	ZINC03978781	412.77	43.83	0.76	Lonicera japonica
MOL003044	Chryseriol	300.28	35.85	0.27	Lonicera japonica
MOL003059	kryptoxanthin	552.96	47.25	0.57	Lonicera japonica
MOL003062	4,5'-Retro-.beta.,.beta.-Carotene-3,3'-dione, 4',5'-didehydro-	562.90	31.22	0.55	Lonicera japonica
MOL003095	5-hydroxy-7-methoxy-2-(3,4,5-trimethoxyphenyl)chromone	358.37	51.96	0.41	Lonicera japonica
MOL003101	7-epi-Vogeloside	432.47	46.13	0.58	Lonicera japonica

MOL003108	Caeruloside C	550.57	55.64	0.73	Lonicera japonica
MOL003111	Centauroside_qt	434.48	55.79	0.50	Lonicera japonica
MOL003117	Ioniceracetalides B_qt	314.37	61.19	0.19	Lonicera japonica
MOL003124	XYLOSTOSIDINE	415.51	43.17	0.64	Lonicera japonica
MOL003128	dinethylsecologanoside	434.44	48.46	0.48	Lonicera japonica
MOL000358	beta-sitosterol	414.79	36.91	0.75	Lonicera japonica
MOL000422	kaempferol	286.25	41.88	0.24	Lonicera japonica
MOL000449	Stigmasterol	412.77	43.83	0.76	Lonicera japonica
MOL000006	luteolin	286.25	36.16	0.25	Lonicera japonica
MOL000098	quercetin	302.25	46.43	0.28	Lonicera japonica
MOL002235	EUPATIN	360.34	50.80	0.41	rheum officinale
MOL002251	Mutatochrome	552.96	48.64	0.61	rheum officinale
MOL002259	Physciondiglucoside	608.60	41.65	0.63	rheum officinale
MOL002260	Procyanidin B-5,3'-O-gallate	730.67	31.99	0.32	rheum officinale
MOL002268	rhein	284.23	47.07	0.28	rheum officinale
MOL002276	Sennoside E_qt	524.50	50.69	0.61	rheum officinale
MOL002280	Torachrysone-8-O-beta-D-(6'-oxayl)-glucoside	480.46	43.02	0.74	rheum officinale
MOL002281	Toralactone	272.27	46.46	0.24	rheum officinale
MOL002288	Emodin-1-O-beta-D-glucopyranoside	432.41	44.81	0.80	rheum officinale
MOL002293	Sennoside D_qt	524.50	61.06	0.61	rheum officinale
MOL002297	Daucosterol_qt	386.73	35.89	0.70	rheum officinale
MOL002303	palmidin A	510.52	32.45	0.65	rheum officinale

MOL000358	beta-sitosterol	414.79	36.91	0.75	rheum officinale
MOL000471	aloe-emodin	270.25	83.38	0.24	rheum officinale
MOL000554	gallic acid-3-O-(6'-O-galloyl)-glucoside	484.40	30.25	0.67	rheum officinale
MOL000096	(-)-catechin	290.29	49.68	0.24	rheum officinale
MOL001645	Linoleyl acetate	308.56	42.10	0.20	Bupleuri Radix
MOL002776	Baicalin	446.39	40.12	0.75	Bupleuri Radix
MOL000449	Stigmasterol	412.77	43.83	0.76	Bupleuri Radix
MOL000354	isorhamnetin	316.28	49.60	0.31	Bupleuri Radix
MOL000422	kaempferol	286.25	41.88	0.24	Bupleuri Radix
MOL004598	3,5,6,7-tetramethoxy-2-(3,4,5-trimethoxyphenyl)chromone	432.46	31.97	0.59	Bupleuri Radix
MOL004609	Areapillin	360.34	48.96	0.41	Bupleuri Radix
MOL013187	Cubebin	356.40	57.13	0.64	Bupleuri Radix
MOL004624	Longikaurin A	348.48	47.72	0.53	Bupleuri Radix
MOL004628	Octalupine	264.41	47.82	0.28	Bupleuri Radix
MOL004644	Sainfuran	286.30	79.91	0.23	Bupleuri Radix
MOL004648	Troxerutin	346.56	31.60	0.28	Bupleuri Radix
MOL004653	(+)-Anomalin	426.50	46.06	0.66	Bupleuri Radix
MOL004702	saikosaponin c_qt	472.78	30.50	0.63	Bupleuri Radix
MOL004718	α-spinasterol	412.77	42.98	0.76	Bupleuri Radix
MOL000490	petunidin	317.29	30.05	0.31	Bupleuri Radix
MOL000098	quercetin	302.25	46.43	0.28	Bupleuri Radix

MOL001689	acacetin	284.28	34.97	0.24	radix isatidis
MOL002322	isovitexin	432.41	31.29	0.72	radix isatidis
MOL001721	Isaindigodione	326.38	60.12	0.41	radix isatidis
MOL001722	2-O-beta-D-glucopyranosyl-2H-1,4-benzoxazin-3(4H)-one	327.32	43.62	0.31	radix isatidis
MOL001726	pinoresinol-4-O-beta-D-apiosyl-beta-D-glucopyranoside	618.74	36.45	0.51	radix isatidis
MOL001728	3-[2′ -(5′ - hydroxymethyl) furyl] -1 (2H) - isoquinolinone-7-O-BETA-D-glucoside_qt	257.26	51.74	0.18	radix isatidis
MOL001733	EUPATORIN	344.34	30.23	0.37	radix isatidis
MOL001734	3-[[(2R,3R,5R,6S)-3,5-dihydroxy-6-(1H-indol-3-yloxy)-4-oxooxan-2-yl]methoxy]-3-oxopropanoic acid	379.35	85.87	0.47	radix isatidis
MOL001735	Dinatin	300.28	30.97	0.27	radix isatidis
MOL001736	(-)-taxifolin	304.27	60.51	0.27	radix isatidis
MOL001749	ZINC03860434	390.62	43.59	0.35	radix isatidis
MOL001750	glucobrassicin	448.52	66.02	0.48	radix isatidis
MOL001755	24-Ethylcholest-4-en-3-one	412.77	36.08	0.76	radix isatidis
MOL001756	quindoline	218.27	33.17	0.22	radix isatidis
MOL001767	hydroxyindirubin	278.28	63.37	0.30	radix isatidis
MOL001769	beta-sitosterol dodecantate	597.13	34.57	0.57	radix isatidis
MOL001771	poriferast-5-en-3beta-ol	414.79	36.91	0.75	radix isatidis
MOL001774	Ineketone	318.50	37.14	0.30	radix isatidis

MOL001779	Sinoacutine	327.41	49.11	0.46	radix isatidis
MOL001781	Indigo	262.28	38.20	0.26	radix isatidis
MOL001782	(2Z)-2-(2-oxoindolin-3-ylidene)indolin-3-one	262.28	48.40	0.26	radix isatidis
MOL001783	2-(9-((3-methyl-2-oxopent-3-en-1-yl)oxy)-2-oxo-1,2,8,9-tetrahydrofuro[2,3-h]quinolin-8-yl)propan-2-yl acetate	399.48	64.00	0.57	radix isatidis
MOL001790	Linarin	592.60	39.84	0.71	radix isatidis
MOL001792	DFV	256.27	32.76	0.18	radix isatidis
MOL001793	(E)-2-[(3-indole)cyanomethylene-]-3-indolinone	300.36	54.59	0.32	radix isatidis
MOL001798	neohesperidin_qt	302.30	71.17	0.27	radix isatidis
MOL001800	rosasterol	414.79	35.87	0.75	radix isatidis
MOL001803	Sinensetin	372.40	50.56	0.45	radix isatidis
MOL001804	Stigmasta-5,22-diene-3beta,7alpha-diol	440.83	43.04	0.82	radix isatidis
MOL001806	Stigmasta-5,22-diene-3beta,7beta-diol	454.86	42.56	0.83	radix isatidis
MOL001810	6-(3-oxoindolin-2-ylidene)indolo[2,1-b]quinazolin-12-one	363.39	45.28	0.89	radix isatidis
MOL001814	(E)-3-(3,5-dimethoxy-4-hydroxy-benzylidene)-2-indolinone	297.33	57.18	0.25	radix isatidis
MOL001820	(E)-3-(3,5-dimethoxy-4-hydroxyb-enzylidene)-2-indolinone	299.35	65.17	0.25	radix isatidis
MOL001828	3-[(3,5-dimethoxy-4-oxo-1-cyclohexa-2,5-dienylidene)methyl]-2,4-dihydro-1H-pyrrolo[2,1-b]quinazolin-9-one	350.40	51.84	0.56	radix isatidis
MOL001833	Glucobrassicin-1-Sulfonate_qt	365.42	42.52	0.24	radix isatidis

MOL000358	beta-sitosterol	414.79	36.91	0.75	radix isatidis
MOL000359	sitosterol	414.79	36.91	0.75	radix isatidis
MOL000449	Stigmasterol	412.77	43.83	0.76	radix isatidis
MOL000953	CLR	386.73	37.87	0.68	radix isatidis
MOL000173	wogonin	284.28	30.68	0.23	Forsythia suspensa(Thunb, Vahl)
MOL003281	20(S)-dammar-24-ene-3β,20-diol-3-acetate	486.86	40.23	0.82	Forsythia suspensa(Thunb, Vahl)
MOL003283	(2R,3R,4S)-4-(4-hydroxy-3-methoxy-phenyl)-7-methoxy-2,3-dimethylol-tetralin-6-ol	360.44	66.51	0.39	Forsythia suspensa(Thunb, Vahl)
MOL003290	(3R,4R)-3,4-bis[(3,4-dimethoxyphenyl)methyl]oxolan-2-one	386.48	52.30	0.48	Forsythia suspensa(Thunb, Vahl)
MOL003295	(+)-pinoresinol monomethyl ether	372.45	53.08	0.57	Forsythia suspensa(Thunb, Vahl)
MOL003305	PHILLYRIN	534.61	36.40	0.86	Forsythia suspensa(Thunb, Vahl)
MOL003306	ACon1_001697	372.45	85.12	0.57	Forsythia suspensa(Thunb, Vahl)
MOL003308	(+)-pinoresinol monomethyl ether-4-D-beta-glucoside_qt	372.45	61.20	0.57	Forsythia suspensa(Thunb, Vahl)
MOL003315	3beta-Acetyl-20,25-epoxydammarane-24alpha-ol	502.86	33.07	0.79	Forsythia suspensa(Thunb, Vahl)
MOL000211	Mairin	456.78	55.38	0.78	Forsythia suspensa(Thunb, Vahl)
MOL003322	FORSYTHINOL	372.45	81.25	0.57	Forsythia suspensa(Thunb, Vahl)

MOL003330	(-)-Phillygenin	372.45	95.04	0.57	Forsythia suspensa(Thunb, Vahl)
MOL003344	β-amyrin acetate	468.84	42.06	0.74	Forsythia suspensa(Thunb, Vahl)
MOL003347	hyperforin	536.87	44.03	0.60	Forsythia suspensa(Thunb, Vahl)
MOL003348	adhyperforin	550.90	44.03	0.61	Forsythia suspensa(Thunb, Vahl)
MOL003365	Lactucasterol	426.75	40.99	0.85	Forsythia suspensa(Thunb, Vahl)
MOL003370	Onjixanthone I	302.30	79.16	0.30	Forsythia suspensa(Thunb, Vahl)
MOL000358	beta-sitosterol	414.79	36.91	0.75	Forsythia suspensa(Thunb, Vahl)
MOL000422	kaempferol	286.25	41.88	0.24	Forsythia suspensa(Thunb, Vahl)
MOL000522	arctiin	534.61	34.45	0.84	Forsythia suspensa(Thunb, Vahl)
MOL000006	luteolin	286.25	36.16	0.25	Forsythia suspensa(Thunb, Vahl)
MOL000791	bicuculline	367.38	69.67	0.88	Forsythia suspensa(Thunb, Vahl)
MOL000098	quercetin	302.25	46.43	0.28	Forsythia suspensa(Thunb, Vahl)
MOL002235	EUPATIN	360.34	50.80	0.41	Artemisia apiacea
MOL000354	isorhamnetin	316.28	49.60	0.31	Artemisia apiacea
MOL000359	sitosterol	414.79	36.91	0.75	Artemisia apiacea
MOL004083	Tamarixetin	316.28	32.86	0.31	Artemisia apiacea

MOL004112	Patuletin	332.28	53.11	0.34	Artemisia apiacea
MOL000422	kaempferol	286.25	41.88	0.24	Artemisia apiacea
MOL000449	Stigmasterol	412.77	43.83	0.76	Artemisia apiacea
MOL004609	Areapillin	360.34	48.96	0.41	Artemisia apiacea
MOL005229	Artemetin	388.40	49.55	0.48	Artemisia apiacea
MOL000006	luteolin	286.25	36.16	0.25	Artemisia apiacea
MOL007274	Skrofulein	314.31	30.35	0.30	Artemisia apiacea
MOL007389	artemisitene	280.35	54.36	0.31	Artemisia apiacea
MOL007400	vicenin-2_qt	270.25	45.84	0.21	Artemisia apiacea
MOL007401	Cirsiliol	330.31	43.46	0.34	Artemisia apiacea
MOL007404	vitexin_qt	270.25	52.18	0.21	Artemisia apiacea
MOL007412	DMQT	346.31	42.60	0.37	Artemisia apiacea
MOL007415	[(2S)-2-[[(2S)-2-(benzoylamino)-3-phenylpropanoyl]amino]-3-phenylpropyl] acetate	444.57	58.02	0.52	Artemisia apiacea
MOL007423	6,8-di-c-glucosylapigenin_qt	270.25	59.85	0.21	Artemisia apiacea
MOL007424	artemisinin	282.37	49.88	0.31	Artemisia apiacea
MOL007425	dihydroartemisinin	284.39	50.75	0.30	Artemisia apiacea
MOL007426	deoxyartemisinin	266.37	54.47	0.26	Artemisia apiacea
MOL000098	quercetin	302.25	46.43	0.28	Artemisia apiacea
MOL001677	asperglaucide	444.57	58.02	0.52	rhizoma anemarrhenae
MOL003773	Mangiferolic acid	442.75	36.16	0.84	rhizoma anemarrhenae
MOL000422	kaempferol	286.25	41.88	0.24	rhizoma anemarrhenae

MOL004373	Anhydroicaritin	368.41	45.41	0.44	rhizoma anemarrhenae
MOL004489	Anemarsaponin F_qt	432.71	60.06	0.79	rhizoma anemarrhenae
MOL004492	Chrysanthemaxanthin	584.96	38.72	0.58	rhizoma anemarrhenae
MOL004497	Hippeastrine	315.35	51.65	0.62	rhizoma anemarrhenae
MOL004514	Timosaponin B III_qt	416.71	35.26	0.87	rhizoma anemarrhenae
MOL000449	Stigmasterol	412.77	43.83	0.76	rhizoma anemarrhenae
MOL004528	Icariin I	676.73	41.58	0.61	rhizoma anemarrhenae
MOL004540	Anemarsaponin C_qt	416.71	35.50	0.87	rhizoma anemarrhenae
MOL004542	Anemarsaponin E_qt	448.76	30.67	0.86	rhizoma anemarrhenae
MOL000483	(Z)-3-(4-hydroxy-3-methoxy-phenyl)-N-[2-(4-hydroxyphenyl)ethyl]acrylamide	313.38	118.35	0.26	rhizoma anemarrhenae
MOL000546	diosgenin	414.69	80.88	0.81	rhizoma anemarrhenae
MOL000631	coumaroyltyramine	283.35	112.90	0.20	rhizoma anemarrhenae
MOL006554	Taraxerol	426.80	38.40	0.77	Dandelion
MOL000098	quercetin	302.25	46.43	0.28	Dandelion
MOL002680	Flavoxanthin	584.96	60.41	0.56	Dandelion
MOL004492	Chrysanthemaxanthin	584.96	38.72	0.58	Dandelion

Supplementary Table S3：

Based on the GENECARDS and OMIM databases, a total of 810 targets directly and indirectly associated with influenza were obtained supplementary Table S3.

Gene Symbol	GC Id	Source
NEU1	GC06M031857	GENECARDS
IRF7	GC11M000612	GENECARDS
DDX58	GC09M032455	GENECARDS
TNF	GC06P047305	GENECARDS

IFNB1	GC09M021077	GENECARDS
IFNA1	GC09P021494	GENECARDS
MX1	GC21P041420	GENECARDS
GP2	GC16M020309	GENECARDS
CD4	GC12P006786	GENECARDS
BACE1	GC11M117285	GENECARDS
CA1	GC08M085327	GENECARDS
CD8A	GC02M086784	GENECARDS
TRIM25	GC17M056836	GENECARDS
ST6GAL1	GC03P186930	GENECARDS
TGM3	GC20P002276	GENECARDS
POLI	GC18P054274	GENECARDS
HAS1	GC19M051714	GENECARDS
RAB39B	GC0XM155259	GENECARDS
IFI30	GC19P018173	GENECARDS
LGALS7	GC19M038770	GENECARDS
SCARA3	GC08P027633	GENECARDS
IFNA17	GC09M021227	GENECARDS
LGALS7B	GC19P038790	GENECARDS
IFITM3	GC11M000319	GENECARDS
TMPRSS2	GC21M041464	GENECARDS
PML	GC15P073994	GENECARDS
IL1B	GC02M112829	GENECARDS
IL6	GC07P022765	GENECARDS
IL10	GC01M206767	GENECARDS
CCR5	GC03P046383	GENECARDS
IL1A	GC02M112773	GENECARDS
CXCL10	GC04M076021	GENECARDS
HIF1A	GC14P061695	GENECARDS
FCGR2A	GC01P161505	GENECARDS
CALCA	GC11M014945	GENECARDS
CCL2	GC17P034255	GENECARDS
TGFB1	GC19M041301	GENECARDS
HLA-G	GC06P047256	GENECARDS
CD209	GC19M007739	GENECARDS
TLR3	GC04P186059	GENECARDS
SFTPD	GC10M079937	GENECARDS
SFTPB	GC02M085657	GENECARDS
IL21	GC04M122612	GENECARDS
PTPN11	GC12P112418	GENECARDS
PRPF8	GC17M001650	GENECARDS
HLA-B	GC06M031315	GENECARDS
MBL2	GC10M052760	GENECARDS

HLA-DRB1	GC06M032578	GENECARDS
CLK1	GC02M200853	GENECARDS
SON	GC21P033542	GENECARDS
FN1	GC02M215360	GENECARDS
CD55	GC01P207321	GENECARDS
MRC1	GC10P017809	GENECARDS
SOCS1	GC16M011255	GENECARDS
DLG2	GC11M083455	GENECARDS
F2	GC11P046720	GENECARDS
IL21R	GC16P027413	GENECARDS
GATAD2A	GC19P023337	GENECARDS
MIR150	GC19M049500	GENECARDS
PRKRA	GC02M178431	GENECARDS
STAU1	GC20M049113	GENECARDS
NAMPT	GC07M106248	GENECARDS
PLCG1	GC20P041136	GENECARDS
FST	GC05P053480	GENECARDS
CD274	GC09P005450	GENECARDS
MIR939	GC08M144394	GENECARDS
PLD2	GC17P004808	GENECARDS
XPO1	GC02M061445	GENECARDS
NXF1	GC11M063441	GENECARDS
BCL2	GC18M063123	GENECARDS
CASP3	GC04M184627	GENECARDS
BAX	GC19P048954	GENECARDS
NFE2L2	GC02M177227	GENECARDS
HLA-A	GC06P047265	GENECARDS
ST3GAL1	GC08M133454	GENECARDS
HMGB1	GC13M030456	GENECARDS
TTR	GC18P031557	GENECARDS
VEGFA	GC06P043770	GENECARDS
CRP	GC01M159716	GENECARDS
RSAD2	GC02P006865	GENECARDS
IVNS1ABP	GC01M185295	GENECARDS
ABCB1	GC07M087504	GENECARDS
DNMT1	GC19M010133	GENECARDS
ACE2	GC0XM015562	GENECARDS
PLG	GC06P160702	GENECARDS
PIK3CG	GC07P106865	GENECARDS
HLA-DQB1	GC06M032804	GENECARDS
IL10RB	GC21P033266	GENECARDS
P2RY11	GC19P010149	GENECARDS
MPP2	GC17M043875	GENECARDS

ZNF365	GC10P062374	GENECARDS
PPAN	GC19P010107	GENECARDS
PPAN-P2RY11	GC19P010108	GENECARDS
KIR2DS5	GC19Mr00079	GENECARDS
KIR2DL5A	GC19MR00046	GENECARDS
KIR3DS1	GC19MR00058	GENECARDS
KIR2DL5B	GC19MP00133	GENECARDS
TRA	GC14P021621	GENECARDS
SNORD105	GC19P010150	GENECARDS
TRB	GC07P145138	GENECARDS
ELANE	GC19P000854	GENECARDS
TXN	GC09M110243	GENECARDS
KPNA6	GC01P032108	GENECARDS
MIR146A	GC05P160485	GENECARDS
MMP9	GC20P046008	GENECARDS
MYC	GC08P127735	GENECARDS
JUN	GC01M058780	GENECARDS
B2M	GC15P044711	GENECARDS
CYP17A1	GC10M102830	GENECARDS
CDKN1B	GC12P012716	GENECARDS
HSPD1	GC02M197486	GENECARDS
ISG15	GC01P001001	GENECARDS
ATP6V1B2	GC08P020197	GENECARDS
PRPS1	GC0XP107628	GENECARDS
ATP6AP2	GC0XP040582	GENECARDS
ATP6V1A	GC03P113747	GENECARDS
COPB2	GC03M139355	GENECARDS
PSENEN	GC19P038231	GENECARDS
RPS14	GC05M150443	GENECARDS
PTPRN	GC02M219289	GENECARDS
TNK2	GC03M195863	GENECARDS
SF3B1	GC02M197389	GENECARDS
GCLC	GC06M053497	GENECARDS
AP2M1	GC03P184174	GENECARDS
ATP6V0D1	GC16M067438	GENECARDS
DBT	GC01M100186	GENECARDS
EIF4A3	GC17M080135	GENECARDS
RETN	GC19P007669	GENECARDS
KPNB1	GC17P047649	GENECARDS
TXNL4A	GC18M079970	GENECARDS
TFE3	GC0XM049028	GENECARDS
NUP98	GC11M003671	GENECARDS
NAA10	GC0XM153929	GENECARDS

ATP6AP1	GC0XP154428	GENECARDS
ATP6V0C	GC16P002513	GENECARDS
EIF3A	GC10M119034	GENECARDS
COPA	GC01M160288	GENECARDS
HSF4	GC16P067164	GENECARDS
RPS5	GC19P058386	GENECARDS
LHX3	GC09M136196	GENECARDS
WNT9A	GC01M227920	GENECARDS
CD58	GC01M116514	GENECARDS
OPN1SW	GC07M128772	GENECARDS
AKTIP	GC16M053492	GENECARDS
CHST5	GC16M075528	GENECARDS
TBL3	GC16P002426	GENECARDS
ITLN1	GC01M160876	GENECARDS
KANSL1	GC17M046031	GENECARDS
ATCAY	GC19P003880	GENECARDS
SNRNP70	GC19P049085	GENECARDS
SAFB	GC19P005623	GENECARDS
LARP1	GC05P154682	GENECARDS
XAB2	GC19M007619	GENECARDS
MED6	GC14M070581	GENECARDS
NUP205	GC07P135557	GENECARDS
UBAC2	GC13P099200	GENECARDS
TRERF1	GC06M042224	GENECARDS
COPG1	GC03P129249	GENECARDS
TSSK6	GC19M019514	GENECARDS
WDR83	GC19P012666	GENECARDS
DTX3	GC12P057604	GENECARDS
PRSS27	GC16M002713	GENECARDS
SF3B6	GC02M024067	GENECARDS
TRIM60	GC04P165032	GENECARDS
BARHL2	GC01M090644	GENECARDS
TRMT61A	GC14P103529	GENECARDS
PPP1R14D	GC15M040815	GENECARDS
PLPPR4	GC01P099262	GENECARDS
H2BC15	GC06P028802	GENECARDS
TSPOAP1	GC17M058302	GENECARDS
EPB41L4A-DT	GC05P112421	GENECARDS
TRIM21	GC11M004384	GENECARDS
PRPF19	GC11M060890	GENECARDS
MIRLET7C	GC21P016551	GENECARDS
IFITM1	GC11P000313	GENECARDS
IFITM2	GC11P000300	GENECARDS

NCR1	GC19P054906	GENECARDS
SFTPA2	GC10M079563	GENECARDS
PLAU	GC10P073909	GENECARDS
HPGD	GC04M174490	GENECARDS
RUNX1	GC21M034787	GENECARDS
ATP1A2	GC01P160115	GENECARDS
CD81	GC11P002377	GENECARDS
PIN1	GC19P009835	GENECARDS
KIF11	GC10P092593	GENECARDS
CEL	GC09P133061	GENECARDS
MYOD1	GC11P017741	GENECARDS
SIGMAR1	GC09M034634	GENECARDS
ATP2C1	GC03P130850	GENECARDS
NEK9	GC14M075079	GENECARDS
PIK3R5	GC17M008878	GENECARDS
TCF3	GC19M001609	GENECARDS
CYP2U1	GC04P107931	GENECARDS
EPHB6	GC07P144968	GENECARDS
GRIN2C	GC17M074842	GENECARDS
CRYAA	GC21P043169	GENECARDS
IL17RA	GC22P017086	GENECARDS
PSMA1	GC11M014505	GENECARDS
RPL35	GC09M124857	GENECARDS
RPS10	GC06M042209	GENECARDS
NTHL1	GC16M002192	GENECARDS
SLC22A6	GC11M063458	GENECARDS
LINGO1	GC15M077613	GENECARDS
LONP1	GC19M005691	GENECARDS
AHCYL1	GC01P109984	GENECARDS
MATN3	GC02M019992	GENECARDS
MAN2B1	GC19M012663	GENECARDS
FERMT3	GC11P064206	GENECARDS
EEF1A1	GC06M073515	GENECARDS
SNW1	GC14M077717	GENECARDS
RPS27A	GC02P055231	GENECARDS
PLK3	GC01P044799	GENECARDS
PSMD14	GC02P161308	GENECARDS
IRF2	GC04M184387	GENECARDS
KCNJ12	GC17P026750	GENECARDS
PSMD2	GC03P184298	GENECARDS
TNFRSF18	GC01M001203	GENECARDS
SELPLG	GC12M108621	GENECARDS
RACGAP1	GC12M049978	GENECARDS

XPNPEP1	GC10M109864	GENECARDS
CLIC4	GC01P024745	GENECARDS
NEK8	GC17P028725	GENECARDS
SNX9	GC06P157685	GENECARDS
POLR2L	GC11M000829	GENECARDS
APBB1IP	GC10P026462	GENECARDS
ARTN	GC01P043933	GENECARDS
DCLK2	GC04P150078	GENECARDS
CXCR6	GC03P045982	GENECARDS
RPS16	GC19M039433	GENECARDS
POLR2H	GC03P184361	GENECARDS
RAB6B	GC03M133824	GENECARDS
KATNB1	GC16P057735	GENECARDS
SULF2	GC20M047656	GENECARDS
VNN2	GC06M132743	GENECARDS
BAIAP3	GC16P001333	GENECARDS
FAU	GC11M065120	GENECARDS
CD48	GC01M160648	GENECARDS
DHRS2	GC14P025289	GENECARDS
RFFL	GC17M035006	GENECARDS
EIF3C	GC16P028709	GENECARDS
SF3A1	GC22M030331	GENECARDS
PHF2	GC09P093576	GENECARDS
STAB1	GC03P052495	GENECARDS
ATG13	GC11P046623	GENECARDS
EIF3G	GC19M010115	GENECARDS
DMAP1	GC01P044214	GENECARDS
COPB1	GC11M014436	GENECARDS
PCDH18	GC04M137518	GENECARDS
SNRPF	GC12P095858	GENECARDS
SUPT6H	GC17P028662	GENECARDS
CCNB3	GC0XP050202	GENECARDS
FCHO2	GC05P072955	GENECARDS
CNNM1	GC10P099330	GENECARDS
SNX6	GC14M034561	GENECARDS
SMU1	GC09M033041	GENECARDS
TRIM14	GC09M098035	GENECARDS
RNF150	GC04M140859	GENECARDS
GNRH2	GC20P003080	GENECARDS
CLSTN3	GC12P008242	GENECARDS
ENGASE	GC17P079071	GENECARDS
ZBTB2	GC06M151364	GENECARDS
SRRM2	GC16P004234	GENECARDS

MAP3K7CL	GC21P029077	GENECARDS
FBXW10	GC17P018832	GENECARDS
CLUH	GC17M002689	GENECARDS
GPR146	GC07P001044	GENECARDS
TAMM41	GC03M011721	GENECARDS
RBM42	GC19P038222	GENECARDS
CRAMP1	GC16P001644	GENECARDS
STYXL2	GC01P167095	GENECARDS
PIK3R2	GC19P018153	GENECARDS
IRF3	GC19M049659	GENECARDS
NEU2	GC02P233032	GENECARDS
ICAM1	GC19P010270	GENECARDS
BRAF	GC07M140719	GENECARDS
GATA3	GC10P008045	GENECARDS
AHR	GC07P016916	GENECARDS
NR2F2	GC15P096325	GENECARDS
MITF	GC03P069788	GENECARDS
PNLIP	GC10P116545	GENECARDS
SLC18A2	GC10P117241	GENECARDS
GABRG2	GC05P162000	GENECARDS
DMD	GC0XM031047	GENECARDS
SLC12A5	GC20P046021	GENECARDS
ADAM12	GC10M126012	GENECARDS
PTPRB	GC12M070516	GENECARDS
TRPS1	GC08M115408	GENECARDS
PIP4K2A	GC10M022484	GENECARDS
B4GALT1	GC09M033100	GENECARDS
NANS	GC09P098056	GENECARDS
SLC1A4	GC02P064988	GENECARDS
PTGFR	GC01P078303	GENECARDS
TRPC4	GC13M037636	GENECARDS
TFDP1	GC13P113584	GENECARDS
FGF12	GC03M192139	GENECARDS
CTNNA2	GC02P079185	GENECARDS
CLCN4	GC0XP010085	GENECARDS
POLR3B	GC12P106357	GENECARDS
PLTP	GC20M045898	GENECARDS
SLC30A2	GC01M026048	GENECARDS
NUMA1	GC11M072002	GENECARDS
FANCM	GC14P045135	GENECARDS
MEIS1	GC02P066433	GENECARDS
GTF2IRD1	GC07P074461	GENECARDS
CCNG1	GC05P163438	GENECARDS

PDCD6IP	GC03P033798	GENECARDS
NRG3	GC10P083672	GENECARDS
SPRED2	GC02M065307	GENECARDS
ADD2	GC02M070626	GENECARDS
APLP1	GC19P038238	GENECARDS
MDC1	GC06M030896	GENECARDS
FKBP1B	GC02P024033	GENECARDS
ACSL3	GC02P222860	GENECARDS
GTF2H4	GC06P047289	GENECARDS
CEP290	GC12M088049	GENECARDS
MMP21	GC10M125766	GENECARDS
DDX18	GC02P117878	GENECARDS
PTPRR	GC12M070638	GENECARDS
DHX16	GC06M030653	GENECARDS
MICA	GC06P031399	GENECARDS
LRP1B	GC02M140231	GENECARDS
MAN1C1	GC01P025631	GENECARDS
AGFG1	GC02P227473	GENECARDS
ABCF1	GC06P030571	GENECARDS
EXTL1	GC01P026032	GENECARDS
DPP10	GC02P114442	GENECARDS
CD207	GC02M070830	GENECARDS
DDX39B	GC06M031530	GENECARDS
PCDH7	GC04P030722	GENECARDS
ZFP57	GC06M029672	GENECARDS
LZTS1	GC08M020246	GENECARDS
PPP1R10	GC06M030600	GENECARDS
ENPP6	GC04M184088	GENECARDS
KCNU1	GC08P036784	GENECARDS
CNKSR1	GC01P026178	GENECARDS
GHITM	GC10P084139	GENECARDS
GIPC2	GC01P077987	GENECARDS
ADGRL2	GC01P081306	GENECARDS
HLA-DQA2	GC06P032741	GENECARDS
NFKBIL1	GC06P047302	GENECARDS
SLITRK5	GC13P087671	GENECARDS
TUBGCP2	GC10M133278	GENECARDS
TRIM39	GC06P047275	GENECARDS
NCAPG2	GC07M158631	GENECARDS
TRIM26	GC06M030184	GENECARDS
VAX2	GC02P070900	GENECARDS
GNL1	GC06M030541	GENECARDS
ARAP2	GC04M035950	GENECARDS

FIGLA	GC02M070741	GENECARDS
ESYT2	GC07M158730	GENECARDS
BUD13	GC11M116749	GENECARDS
CORO2A	GC09M098120	GENECARDS
DRAP1	GC11P065926	GENECARDS
MRPS18B	GC06P030617	GENECARDS
SPAG6	GC10P022345	GENECARDS
IGSF11	GC03M118900	GENECARDS
SNX13	GC07M017798	GENECARDS
L3MBTL3	GC06P130013	GENECARDS
TCF19	GC06P047294	GENECARDS
TBC1D2	GC09M098198	GENECARDS
TEKT1	GC17M006789	GENECARDS
DHX32	GC10M125836	GENECARDS
KIF15	GC03P045351	GENECARDS
LINGO2	GC09M027940	GENECARDS
AFTPH	GC02P064524	GENECARDS
ARPP21	GC03P035680	GENECARDS
CSMD3	GC08M112223	GENECARDS
NEUROD4	GC12P055023	GENECARDS
PFN4	GC02M024115	GENECARDS
PXDNL	GC08M051319	GENECARDS
TMCO3	GC13P113490	GENECARDS
TTLL7	GC01M083865	GENECARDS
RPL10L	GC14M046651	GENECARDS
ZNF703	GC08P037695	GENECARDS
ARRDC4	GC15P097960	GENECARDS
RIC8B	GC12P106774	GENECARDS
PNLIPRP3	GC10P116427	GENECARDS
FANK1	GC10P125896	GENECARDS
CLEC4F	GC02M070808	GENECARDS
SPATA8	GC15P096783	GENECARDS
SPINK4	GC09P033230	GENECARDS
SERHL2	GC22P042553	GENECARDS
SAMD3	GC06M130144	GENECARDS
SLITRK4	GC0XM143622	GENECARDS
UBXN2A	GC02P023927	GENECARDS
WWC3	GC0XP010015	GENECARDS
ATAD2B	GC02M023711	GENECARDS
CEP128	GC14M080476	GENECARDS
MUC21	GC06P047291	GENECARDS
NRM	GC06M030895	GENECARDS
SELENON	GC01P025800	GENECARDS

KLHL29	GC02P023348	GENECARDS
DYNC2I1	GC07P158839	GENECARDS
POLR1H	GC06P048997	GENECARDS
PSORS1C2	GC06M031137	GENECARDS
TMEM200A	GC06P130365	GENECARDS
C6orf136	GC06P047284	GENECARDS
SERTAD2	GC02M064631	GENECARDS
TSGA10IP	GC11P065947	GENECARDS
TMEM263	GC12P106955	GENECARDS
OR9K2	GC12P055129	GENECARDS
MEDAG	GC13P030906	GENECARDS
MTERF2	GC12M106977	GENECARDS
TMPRSS11F	GC04M068053	GENECARDS
TRMO	GC09M097896	GENECARDS
CFAP74	GC01M001921	GENECARDS
HLA-H	GC06P047258	GENECARDS
MUC22	GC06P031005	GENECARDS
HLA-J	GC06P047267	GENECARDS
MIR877	GC06P030584	GENECARDS
HCG18	GC06M030874	GENECARDS
SFTA1P	GC10M010784	GENECARDS
TUSC7	GC03P116709	GENECARDS
HLA-F-AS1	GC06M030844	GENECARDS
HCG4B	GC06M030862	GENECARDS
TEX26-AS1	GC13M030881	GENECARDS
MIR622	GC13P090231	GENECARDS
HLA-L	GC06P047274	GENECARDS
RRP7BP	GC22M042555	GENECARDS
MDFIC2	GC03M070197	GENECARDS
HLA-K	GC06P047263	GENECARDS
LOC339166	GC17P005773	GENECARDS
ATP6V1B1-AS1	GC02M070941	GENECARDS
PRANCR	GC12M069901	GENECARDS
LINC00709	GC10P009275	GENECARDS
MICE	GC06M030847	GENECARDS
ETF1P1	GC06P047268	GENECARDS
HLA-W	GC06P047261	GENECARDS
LINC02153	GC08P020974	GENECARDS
LINC02889	GC07M017410	GENECARDS
LINC02254	GC15M103983	GENECARDS
HLA-U	GC06P047264	GENECARDS
PAIP1P1	GC06M030186	GENECARDS

ENSG00000204929	GC02P065439	GENECARDS
RPL3P2	GC06P031280	GENECARDS
ENSG00000233290	GC01M082215	GENECARDS
ENSG00000234255	GC02M065442	GENECARDS
SUMO2P1	GC06M029639	GENECARDS
ENSG00000254186	GC05M162425	GENECARDS
LINC01811	GC03P033956	GENECARDS
ENSG00000260329	GC12M106954	GENECARDS
RNA5SP22	GC01M078094	GENECARDS
RNA5SP328	GC10M126145	GENECARDS
HMGB3P8	GC10M116442	GENECARDS
ENSG00000239268	GC03M117672	GENECARDS
ENSG00000233478	GC01M025644	GENECARDS
ENSG00000228675	GC07P017719	GENECARDS
ENSG00000227678	GC06M130133	GENECARDS
ENSG00000232353	GC03P192029	GENECARDS
ENSG00000233258	GC10M083881	GENECARDS
ENSG00000254092	GC08M020952	GENECARDS
TRP-GGG1-1	GC10P022563	GENECARDS
ENSG00000254260	GC08P020941	GENECARDS
OR9K1P	GC12P055115	GENECARDS
ENSG00000253344	GC08M037626	GENECARDS
ENSG00000272540	GC06M030903	GENECARDS
LINC02253	GC15P097771	GENECARDS
LINC02334	GC13P037935	GENECARDS
LINC02336	GC13P089402	GENECARDS
ENSG00000285471	GC17P005775	GENECARDS

RN7SKP181	GC15P096842	GENECARDS
DHFRP3	GC02P082856	GENECARDS
RPL30P12	GC12P106910	GENECARDS
HMGN2P21	GC02P070803	GENECARDS
SEC63P2	GC04P035491	GENECARDS
ENSG00000237498	GC02M082479	GENECARDS
ENSG00000223808	GC10P008943	GENECARDS
LOC105379752	GC07M158777	GENECARDS
ENSG00000258845	GC14P045567	GENECARDS
ENSG00000233870	GC02P071178	GENECARDS
RF00017-7760	GC09P097907	GENECARDS
ENSG00000287981	GC03P034152	GENECARDS
lnc-C10orf90-5	GC10M126242	GENECARDS
lnc-FRMD4B-4	GC03M069763	GENECARDS
lnc-WDR60-13	GC07P158829	GENECARDS
MN298639	GC09M098376	GENECARDS
piR-50443-383	GC09P098243	GENECARDS
lnc-EBLN1-6	GC10M022536	GENECARDS
lnc-DHX32-4	GC10M126119	GENECARDS
lnc-ESYT2-1	GC07M158793	GENECARDS
RNU6-129P	GC10P083764	GENECARDS
ENSG00000237653	GC03M191952	GENECARDS
ENSG00000237669	GC06M030910	GENECARDS
ENSG00000238694	GC04M034967	GENECARDS
ENSG00000201311	GC02M082307	GENECARDS
piR-45783	GC07P158811	GENECARDS
lnc-NANS-4	GC09P098255	GENECARDS
ENSG00000230521	GC06M030856	GENECARDS
JA662191	GC20P046009	GENECARDS
lnc-PGPEP1L-16	GC15M104009	GENECARDS
lnc-PGPEP1L-18	GC15M104011	GENECARDS
piR-47086-092	GC12P070765	GENECARDS
CARS1P2	GC08M114794	GENECARDS

ABCF1-DT	GC06M030908	GENECARDS
lnc-AUNIP-6	GC01M025692	GENECARDS
MN309174-118	GC12M070867	GENECARDS
LOC105374780	GC02M065439	GENECARDS
piR-38005-040	GC03M069920	GENECARDS
RF00001-007	GC01M078095	GENECARDS
RF00001-052	GC10M126146	GENECARDS
piR-40476-044	GC03M034097	GENECARDS
piR-32662	GC12M055052	GENECARDS
ENSG00000286370	GC02P117536	GENECARDS
piR-32023-108	GC02M070799	GENECARDS
MF281430-014	GC12M106912	GENECARDS
lnc-RIC8B-1	GC12P106911	GENECARDS
piR-59769-333	GC02P023906	GENECARDS
HSALNG0049163	GC06M030892	GENECARDS
HSALNG0067880	GC08P115073	GENECARDS
LOC105378000	GC06M130120	GENECARDS
ENSG00000227291	GC02M117180	GENECARDS
NONHSAG043414.2	GC06P047947	GENECARDS
LOC105371005	GC15M097106	GENECARDS
piR-36588-229	GC02M070801	GENECARDS
LOC107984997	GC01M078125	GENECARDS
ENSG00000258746	GC14M045502	GENECARDS
piR-37026-125	GC12M055098	GENECARDS
ENSG00000275250	GC04P034714	GENECARDS
lnc-POLDIP3-3	GC22M045584	GENECARDS
HSALNG0022753	GC02M227556	GENECARDS
MAPK14	GC06P047451	GENECARDS
S1PR1	GC01P101236	GENECARDS
FCN3	GC01M027379	GENECARDS
IL5	GC05M132541	GENECARDS
CXCL8	GC04P073740	GENECARDS
TP53	GC17M007661	GENECARDS
KLK5	GC19M050943	GENECARDS
CXCR3	GC0XM071615	GENECARDS
CCL5	GC17M035871	GENECARDS
DHX58	GC17M042101	GENECARDS
PTPA	GC09P129111	GENECARDS

MIR584	GC05M149062	GENECARDS
MIR1249	GC22M045200	GENECARDS
CRKL	GC22P020917	GENECARDS
TLR7	GC0XP012867	GENECARDS
F2RL1	GC05P076818	GENECARDS
IL15	GC04P141636	GENECARDS
GLDC	GC09M006522	GENECARDS
NCL	GC02M231453	GENECARDS
IFNL3	GC19M039243	GENECARDS
MIR22	GC17M001713	GENECARDS
IFNG	GC12M068064	GENECARDS
ITGA5	GC12M054396	GENECARDS
ITGB6	GC02M160099	GENECARDS
CCR1	GC03M046218	GENECARDS
TUFM	GC16M028858	GENECARDS
ERAP1	GC05M096760	GENECARDS
CLEC4M	GC19P007763	GENECARDS
NLRX1	GC11P119166	GENECARDS
FCN1	GC09M134975	GENECARDS
TMPRSS11D	GC04M067820	GENECARDS
MIR26A1	GC03P037969	GENECARDS
MIR335	GC07P130496	GENECARDS
MIR628	GC15M055372	GENECARDS
MIR576	GC04P109488	GENECARDS
MIR664A	GC01M220200	GENECARDS
MIR1260A	GC14P077266	GENECARDS
MAVS	GC20P003827	GENECARDS
MMP2	GC16P055390	GENECARDS
APP	GC21M025880	GENECARDS
CAMK2B	GC07M044217	GENECARDS
RELA	GC11M065653	GENECARDS
ATP1A1	GC01P116372	GENECARDS
UBE2I	GC16P001415	GENECARDS
PDCD1	GC02M241849	GENECARDS
AQP3	GC09M033431	GENECARDS
FGF2	GC04P122826	GENECARDS
SCNN1A	GC12M006346	GENECARDS
PTK2	GC08M140657	GENECARDS
RHOA	GC03M049359	GENECARDS
SUMO1	GC02M202206	GENECARDS
FPR2	GC19P051752	GENECARDS
CCR7	GC17M040556	GENECARDS
AQP1	GC07P030911	GENECARDS

AQP5	GC12P049961	GENECARDS
CD27	GC12P008144	GENECARDS
IKBKE	GC01P206470	GENECARDS
HAMP	GC19P038216	GENECARDS
CEACAM1	GC19M042507	GENECARDS
DUOX2	GC15M045092	GENECARDS
IL9	GC05M135891	GENECARDS
IL17A	GC06P052186	GENECARDS
CAMP	GC03P048266	GENECARDS
DUOX1	GC15P045129	GENECARDS
IFNA2	GC09M021384	GENECARDS
CCL4	GC17P036103	GENECARDS
CXCL11	GC04M076033	GENECARDS
TLR10	GC04M038773	GENECARDS
IL32	GC16P004242	GENECARDS
CPSF1	GC08M144393	GENECARDS
BTLA	GC03M112463	GENECARDS
HERC5	GC04P088457	GENECARDS
CUEDC2	GC10M102424	GENECARDS
CPSF3	GC02P009423	GENECARDS
FYB1	GC05M039105	GENECARDS
IGLL5	GC22P024570	GENECARDS
MIR155	GC21P025573	GENECARDS
MIR324	GC17M007223	GENECARDS
SPHK1	GC17P076376	GENECARDS
TRAF6	GC11M036467	GENECARDS
TLR8	GC0XP012924	GENECARDS
CDC25B	GC20P003787	GENECARDS
IL12A	GC03P159988	GENECARDS
ANP32A	GC15M068778	GENECARDS
BST2	GC19M017403	GENECARDS
EBI3	GC19P004232	GENECARDS
IFNL1	GC19P039296	GENECARDS
RTRAF	GC14P051992	GENECARDS
PRKCD	GC03P053156	GENECARDS
RPS6KA3	GC0XM020149	GENECARDS
NFKB1	GC04P102501	GENECARDS
HDAC6	GC0XP048801	GENECARDS
CDC42	GC01P022057	GENECARDS
PCNA	GC20M005114	GENECARDS
SRC	GC20P037344	GENECARDS
PIK3R1	GC05P068215	GENECARDS
TLR2	GC04P153684	GENECARDS

IL2RA	GC10M006010	GENECARDS
IFNGR1	GC06M137197	GENECARDS
GLUL	GC01M182350	GENECARDS
MIF	GC22P023894	GENECARDS
AIFM1	GC0XM130129	GENECARDS
RIPK1	GC06P003064	GENECARDS
CSNK1A1	GC05M149492	GENECARDS
NOS2	GC17M027756	GENECARDS
PTGS2	GC01M186640	GENECARDS
TUBA1A	GC12M049184	GENECARDS
IRF5	GC07P128937	GENECARDS
FADD	GC11P070203	GENECARDS
PIK3CB	GC03M138652	GENECARDS
TLR1	GC04M038797	GENECARDS
TLR5	GC01M223109	GENECARDS
IRF1	GC05M132481	GENECARDS
BAD	GC11M064273	GENECARDS
DDX5	GC17M064498	GENECARDS
TNFSF10	GC03M172505	GENECARDS
RAB11A	GC15P072880	GENECARDS
EZR	GC06M158765	GENECARDS
AICDA	GC12M008602	GENECARDS
ERN1	GC17M064039	GENECARDS
C1QA	GC01P022636	GENECARDS
CRK	GC17M001420	GENECARDS
HNRNPA2B1	GC07M026174	GENECARDS
IFNAR1	GC21P033324	GENECARDS
NUP62	GC19M049906	GENECARDS
TNFSF13B	GC13P108251	GENECARDS
TBX21	GC17P047733	GENECARDS
TLR9	GC03M052222	GENECARDS
IL2	GC04M122451	GENECARDS
MYO6	GC06P075749	GENECARDS
XBP1	GC22M028794	GENECARDS
ADAR	GC01M154582	GENECARDS
CSF2	GC05P132073	GENECARDS
SGPL1	GC10P070815	GENECARDS
POLR2A	GC17P008025	GENECARDS
KLF6	GC10M003779	GENECARDS
TLR6	GC04M038828	GENECARDS
IL3	GC05P132060	GENECARDS
GHRL	GC03M010285	GENECARDS
RIPK3	GC14M024336	GENECARDS

CD244	GC01M160830	GENECARDS
CD70	GC19M006583	GENECARDS
IL18R1	GC02P102311	GENECARDS
PRSS3	GC09P033750	GENECARDS
HSPA1A	GC06P047326	GENECARDS
VAPA	GC18P009904	GENECARDS
GNB2	GC07P100673	GENECARDS
EEF1D	GC08M143579	GENECARDS
KPNA1	GC03M122421	GENECARDS
APOBEC3G	GC22P039078	GENECARDS
MLKL	GC16M074672	GENECARDS
SFTPA1	GC10P084171	GENECARDS
PCBP1	GC02P070087	GENECARDS
NFKBIB	GC19P038899	GENECARDS
IRAK2	GC03P010210	GENECARDS
KHSRP	GC19M006413	GENECARDS
PTX3	GC03P157436	GENECARDS
TMPRSS15	GC21M018269	GENECARDS
CPSF4	GC07P099438	GENECARDS
HNRNPF	GC10M043385	GENECARDS
DHX9	GC01P182839	GENECARDS
DEFA1	GC08M006977	GENECARDS
TOMM40	GC19P044890	GENECARDS
TAF6	GC07M100107	GENECARDS
TMPRSS4	GC11P118077	GENECARDS
METTL3	GC14M021498	GENECARDS
CXCL9	GC04M076001	GENECARDS
COX6C	GC08M099899	GENECARDS
DR1	GC01P093345	GENECARDS
ARHGAP21	GC10M024534	GENECARDS
CMAS	GC12P022046	GENECARDS
DEFA5	GC08M007057	GENECARDS
RNF128	GC0XP106693	GENECARDS
IL27	GC16M028511	GENECARDS
ILF3	GC19P010625	GENECARDS
YTHDF2	GC01P028751	GENECARDS
APOBEC3F	GC22P039024	GENECARDS
DEFA6	GC08M006924	GENECARDS
NXT1	GC20P023350	GENECARDS
TRAPPC6A	GC19M045162	GENECARDS
RACK1	GC05M181310	GENECARDS
MORC3	GC21P036320	GENECARDS
DEFA4	GC08M006935	GENECARDS

MOAP1	GC14M093182	GENECARDS
NUP54	GC04M076114	GENECARDS
KLK12	GC19M051029	GENECARDS
IFNL2	GC19P039268	GENECARDS
LSM14A	GC19P034172	GENECARDS
DNAJB13	GC11P073950	GENECARDS
RRP1B	GC21P043659	GENECARDS
TRIM41	GC05P181222	GENECARDS
CLEC5A	GC07M141927	GENECARDS
DPH7	GC09M137554	GENECARDS
TMEM181	GC06P158536	GENECARDS
MIR29A	GC07M130876	GENECARDS
MIR302A	GC04M112679	GENECARDS
MIR203A	GC14P104655	GENECARDS
MIR4776-1	GC02P212926	GENECARDS
MIR4776-2	GC02M212926	GENECARDS
MIR4276	GC04P174423	GENECARDS
TLR4	GC09P117704	GENECARDS
IL22RA1	GC01M024119	GENECARDS
SOD1	GC21P031659	GENECARDS
ALB	GC04P073397	GENECARDS
HCRTR2	GC06P055106	GENECARDS
VNN1	GC06M132680	GENECARDS
HOXD13	GC02P176092	GENECARDS
MIR141	GC12P008231	GENECARDS
EGFR	GC07P055019	GENECARDS
SOD2	GC06M159669	GENECARDS
TBK1	GC12P064451	GENECARDS
FYN	GC06M111660	GENECARDS
INS	GC11M002159	GENECARDS
ACTN4	GC19P038647	GENECARDS
FURIN	GC15P090868	GENECARDS
APPL1	GC03P057227	GENECARDS
PRSS1	GC07P144938	GENECARDS
ST14	GC11P130159	GENECARDS
AGER	GC06M032180	GENECARDS
IL18	GC11M112143	GENECARDS
KNG1	GC03P186717	GENECARDS
HAVCR2	GC05M157063	GENECARDS
TRIM28	GC19P058544	GENECARDS
POLR1C	GC06P047535	GENECARDS
PF4	GC04M073980	GENECARDS
SOCS5	GC02P046698	GENECARDS

ISG20	GC15P088635	GENECARDS
KLF2	GC19P023268	GENECARDS
SPN	GC16P029662	GENECARDS
BANP	GC16P087949	GENECARDS
ADPRH	GC03P119579	GENECARDS
CLEC4A	GC12P008267	GENECARDS
TMPRSS13	GC11M117900	GENECARDS
IFITM5	GC11M000298	GENECARDS
TMPRSS11E	GC04P068447	GENECARDS
GYPE	GC04M143870	GENECARDS
LINC01191	GC02P115314	GENECARDS
PSMB8-AS1	GC06P032844	GENECARDS
SCGB1C1	ENSG00000188076,ENST00000342878.3	OMIM
ODF3	ENSG00000177947,ENST00000525282.1	OMIM
BET1L	ENSG00000177951,ENST00000382762.8	OMIM
RIC8A	ENSG00000177963,ENST00000526104.6	OMIM
SIRT3	ENSG00000142082,ENST00000524564.5	OMIM
PSMD13	ENSG00000185627,ENST00000532097.6	OMIM
NLRP6	ENSG00000174885,ENST00000534750.6	OMIM
PGGHG	ENSG00000142102,ENST00000409548.7	OMIM
B4GALNT4	ENSG00000182272,ENST00000329962.11	OMIM
PKP3	ENSG00000184363,ENST00000331563.7	OMIM
SIGIRR	ENSG00000185187,ENST00000397632.7	OMIM
PTDSS2	ENSG00000174915,ENST00000308020.6	OMIM
RNH1	ENSG00000023191,ENST00000397615.6	OMIM
LRRC56	ENSG00000161328,ENST00000270115.8	OMIM
HRAS	ENSG00000174775,ENST00000451590.5	OMIM

RASSF7	ENSG00000099849,ENST0000039758 2.7	OMIM
MIR210	ENSG00000199038,ENST0000036216 8.1	OMIM
PHRF1	ENSG00000070047,ENST0000026455 5.10	OMIM
CDHR5	ENSG00000099834,ENST0000035835 3.8	OMIM
SCT	ENSG00000070031,ENST0000017619 5.4	OMIM
DRD4	ENSG00000069696,ENST0000017618 3.6	OMIM
DEAF1	ENSG00000177030,ENST0000068330 7.1	OMIM
EPS8L2	ENSG00000177106,ENST0000031856 2.13	OMIM
TALDO1	ENSG00000177156,ENST0000031900 6.8	OMIM
CEND1	ENSG00000184524,ENST0000033010 6.5	OMIM
SLC25A22	ENSG00000177542,ENST0000053121 4.5	OMIM
PIDD1	ENSG00000177595,ENST0000034775 5.10	OMIM
RPLP2	ENSG00000177600,ENST0000032115 3.9	OMIM
PNPLA2	ENSG00000177666,ENST0000033661 5.9	OMIM
CRACR2B	ENSG00000177685,ENST0000052507 7.2	OMIM
CD151	ENSG00000177697,ENST0000039742 0.9	OMIM
TSPAN4	ENSG00000214063,ENST0000039740 6.5	OMIM
CHID1	ENSG00000177830,ENST0000043610 8.6	OMIM
AP2A2	ENSG00000183020,ENST0000033223 1.9	OMIM
MUC6	ENSG00000184956,ENST0000042167 3.7	OMIM
MUC2	ENSG00000198788,ENST0000036155 8.7	OMIM
MUC5AC	ENSG00000215182,ENST0000062122 6.2	OMIM

MUC5B	ENSG00000117983,ENST0000052968 1.5	OMIM
TOLLIP	ENSG00000078902,ENST0000053054 1.1	OMIM
BRSK2	ENSG00000174672,ENST0000052884 1.6	OMIM
MOB2	ENSG00000182208,ENST0000032995 7.7	OMIM
DUSP8	ENSG00000184545,ENST0000039737 4.8	OMIM
KRTAP5-1	ENSG00000205869,ENST0000038217 1.2	OMIM
IFITM10	ENSG00000244242,ENST0000034013 4.5	OMIM
CTSD	ENSG00000117984,ENST0000023667 1.7	OMIM
SYT8	ENSG00000149043,ENST0000038197 8.7	OMIM
TNNI2	ENSG00000130598,ENST0000038190 6.5	OMIM
LSP1	ENSG00000130592,ENST0000040595 7.6	OMIM
TNNT3	ENSG00000130595,ENST0000038157 9.7	OMIM
MRPL23	ENSG00000214026,ENST0000039729 8.8	OMIM
H19	ENSG00000130600,ENST0000041479 0.7	OMIM
MIR675	ENSG00000284010,ENST0000039016 8.6	OMIM

Supplementary Table S4:

By employing the available TCMSP databases and wide-scale searches of the literature, including PubMed and China National Knowledge Infrastructure (CNKI) databases, we obtained 245 CSTRP related targets supplementary Table S4

Entry	Entry name	Protein names	Gene name
P43115	PE2R3_HUMAN	Prostaglandin E2 receptor EP3 subtype	PTGER3
P11511	CP19A_HUMAN	Aromatase	CYP19A 1
P11802	CDK4_HUMAN	Cyclin-dependent kinase 4	CDK4

P45844	ABCG1_HUMAN	ATP-binding cassette sub-family G member 1	ABCG1
P11021	BIP_HUMAN	Endoplasmic reticulum chaperone BiP	HSPA5
Q01469	FABP5_HUMAN	Fatty acid-binding protein 5	FABP5
P01857	IGHG1_HUMAN	Immunoglobulin heavy constant gamma 1	IGHG1
P29474	NOS3_HUMAN	Nitric oxide synthase, endothelial	NOS3
O43451	MGA_HUMAN	Maltase-glucoamylase, intestinal [Includes: Maltase	MGAM
P80108	PHLD_HUMAN	Phosphatidylinositol-glycan-specific phospholipase D	GPLD1
P01344	IGF2_HUMAN	Insulin-like growth factor II	IGF2
P10415	BCL2_HUMAN	Apoptosis regulator Bcl-2	BCL2
O95433	AHSA1_HUMAN	Activator of 90 kDa heat shock protein ATPase homolog 1	AHSA1
P29965	CD40L_HUMAN	CD40 ligand	CD40LG
P28340	DPOD1_HUMAN	DNA polymerase delta catalytic subunit	POLD1
Q92819	HYAS2_HUMAN	Hyaluronan synthase 2	HAS2
P28482	MK01_HUMAN	Mitogen-activated protein kinase 1	MAPK1
P09848	LPH_HUMAN	Lactase-phlorizin hydrolase	LCT
O14939	PLD2_HUMAN	Phospholipase D2	PLD2
Q04206	TF65_HUMAN	Transcription factor p65	RELA
P05771	KPCB_HUMAN	Protein kinase C beta type	PRKCB
P00441	SODC_HUMAN	Superoxide dismutase [Cu-Zn]	SOD1
Q13393	PLD1_HUMAN	Phospholipase D1	PLD1
P60484	PTEN_HUMAN	Phosphatidylinositol 3,4,5-trisphosphate 3-phosphatase and dual-specificity protein phosphatase PTEN	PTEN
Q8IV08	PLD3_HUMAN	5'-3' exonuclease PLD3	PLD3
P01106	MYC_HUMAN	Myc proto-oncogene protein	MYC
P15309	PPAP_HUMAN	Prostatic acid phosphatase	ACP3
P08254	MMP3_HUMAN	Stromelysin-1	MMP3
P06400	RB_HUMAN	Retinoblastoma-associated protein	RB1
P49585	PCY1A_HUMAN	Choline-phosphate cytidylyltransferase A	PCYT1A
P08294	SODE_HUMAN	Extracellular superoxide dismutase [Cu-Zn]	SOD3
Q8IWA5	CTL2_HUMAN	Choline transporter-like protein 2	SLC44A2
P42574	CASP3_HUMAN	Caspase-3	CASP3
P04798	CP1A1_HUMAN	Cytochrome P450 1A1	CYP1A1

P11836	CD20_HUMAN	B-lymphocyte antigen CD20	MS4A1
P55211	CASP9_HUMAN	Caspase-9	CASP9
P24385	CCND1_HUMAN	G1/S-specific cyclin-D1	CCND1
Q01094	E2F1_HUMAN	Transcription factor E2F1	E2F1
P01584	IL1B_HUMAN	Interleukin-1 beta	IL1B
P60709	ACTB_HUMAN	Actin, cytoplasmic 1	ACTB
P04040	CATA_HUMAN	Catalase	CAT
P09917	LOX5_HUMAN	Polyunsaturated fatty acid 5-lipoxygenase	ALOX5
Q9UII4	HERC5_HUMAN	E3 ISG15--protein ligase HERC5	HERC5
P27361	MK03_HUMAN	Mitogen-activated protein kinase 3	MAPK3
P49895	IOD1_HUMAN	Type I iodothyronine deiodinase	DIO1
Q8IVI9	NOSTN_HUMAN	Nostrin	NOSTRIN
P05231	IL6_HUMAN	Interleukin-6	IL6
P05412	JUN_HUMAN	Transcription factor AP-1	JUN
Q9BYJ1	LOXE3_HUMAN	Hydroperoxide isomerase ALOXE3	ALOXE3
P01375	TNFA_HUMAN	Tumor necrosis factor	TNF
P15692	VEGFA_HUMAN	Vascular endothelial growth factor A	VEGFA
O43242	PSMD3_HUMAN	26S proteasome non-ATPase regulatory subunit 3	PSMD3
P04049	RAF1_HUMAN	RAF proto-oncogene serine/threonine-protein kinase	RAF1
P19793	RXRA_HUMAN	Retinoic acid receptor RXR-alpha	RXRA
Q9NS23	RASF1_HUMAN	Ras association domain-containing protein 1	RASSF1
P23975	SC6A2_HUMAN	Sodium-dependent noradrenaline transporter	SLC6A2
Q13950	RUNX2_HUMAN	Runt-related transcription factor 2	RUNX2
Q14524	SCN5A_HUMAN	Sodium channel protein type 5 subunit alpha	SCN5A
P27169	PON1_HUMAN	Serum paraoxonase/arylesterase 1	PON1
P11137	MTAP2_HUMAN	Microtubule-associated protein 2	MAP2
P03973	SLPI_HUMAN	Antileukoproteinase	SLPI
P78380	OLR1_HUMAN	Oxidized low-density lipoprotein receptor 1	OLR1
P05164	PERM_HUMAN	Myeloperoxidase	MPO
Q01959	SC6A3_HUMAN	Sodium-dependent dopamine transporter	SLC6A3
P04179	SODM_HUMAN	Superoxide dismutase [Mn], mitochondrial	SOD2
P28161	GSTM2_HUMAN	Glutathione S-transferase Mu 2	GSTM2

Q07869	PPARA_HUMAN	Peroxisome proliferator-activated receptor alpha	PPARA
P47712	PA24A_HUMAN	Cytosolic phospholipase A2	PLA2G4 A
P14780	MMP9_HUMAN	Matrix metalloproteinase-9	MMP9
Q99801	NKX31_HUMAN	Homeobox protein Nkx-3.1	NKX3-1
P41143	OPRD_HUMAN	Delta-type opioid receptor	OPRD1
P49841	GSK3B_HUMAN	Glycogen synthase kinase-3 beta	GSK3B
Q14432	PDE3A_HUMAN	cGMP-inhibited 3',5'-cyclic phosphodiesterase A	PDE3A
Q14994	NR1I3_HUMAN	Nuclear receptor subfamily 1 group I member 3	NR1I3
P15941	MUC1_HUMAN	Mucin-1	MUC1
P35228	NOS2_HUMAN	Nitric oxide synthase, inducible	NOS2
Q06455	MTG8_HUMAN	Protein CBFA2T1	RUNX1T 1
P05121	PAI1_HUMAN	Plasminogen activator inhibitor 1	SERPINE 1
P55786	PSA_HUMAN	Puromycin-sensitive aminopeptidase	NPEPPS
P20936	RASA1_HUMAN	Ras GTPase-activating protein 1	RASA1
Q96PH1	NOX5_HUMAN	NADPH oxidase 5	NOX5
P23219	PGH1_HUMAN	Prostaglandin G/H synthase 1	PTGS1
Q05655	KPCD_HUMAN	Protein kinase C delta type	PRKCD
P11387	TOP1_HUMAN	DNA topoisomerase 1	TOP1
P55072	TERA_HUMAN	Transitional endoplasmic reticulum ATPase	VCP
P19320	VCAM1_HUMAN	Vascular cell adhesion protein 1	VCAM1
O14684	PTGES_HUMAN	Prostaglandin E synthase	PTGES
P18031	PTN1_HUMAN	Tyrosine-protein phosphatase non-receptor type 1	PTPN1
P06401	PRGR_HUMAN	Progesterone receptor	PGR
Q00613	HSF1_HUMAN	Heat shock factor protein 1	HSF1
P12004	PCNA_HUMAN	Proliferating cell nuclear antigen	PCNA
O75469	NR1I2_HUMAN	Nuclear receptor subfamily 1 group I member 2	NR1I2
Q15788	NCOA1_HUMAN	Nuclear receptor coactivator 1	NCOA1
P17612	KAPCA_HUMAN	cAMP-dependent protein kinase catalytic subunit alpha	PRKACA
Q13162	PRDX4_HUMAN	Peroxiredoxin-4	PRDX4
P13726	TF_HUMAN	Tissue factor	F3
Q99973	TEP1_HUMAN	Telomerase protein component 1	TEP1
P11388	TOP2A_HUMAN	DNA topoisomerase 2-alpha	TOP2A

Q9Y233	PDE10_HUMAN	cAMP and cAMP-inhibited cGMP 3',5'-cyclic phosphodiesterase 10A	PDE10A
P09874	PARP1_HUMAN	Poly [ADP-ribose] polymerase 1	PARP1
P04637	P53_HUMAN	Cellular tumor antigen p53	TP53
P00750	TPA_HUMAN	Tissue-type plasminogen activator	PLAT
P31645	SC6A4_HUMAN	Sodium-dependent serotonin transporter	SLC6A4
P42224	STAT1_HUMAN	Signal transducer and activator of transcription 1-alpha/beta	STAT1
P11217	PYGM_HUMAN	Glycogen phosphorylase, muscle form	PYGM
P08912	ACM5_HUMAN	Muscarinic acetylcholine receptor M5	CHRM5
P10275	ANDR_HUMAN	Androgen receptor	AR
O14625	CXL11_HUMAN	C-X-C motif chemokine 11	CXCL11
Q13255	GRM1_HUMAN	Metabotropic glutamate receptor 1	GRM1
P07339	CATD_HUMAN	Cathepsin D	CTSD
P09488	GSTM1_HUMAN	Glutathione S-transferase Mu 1	GSTM1
P08684	CP3A4_HUMAN	Cytochrome P450 3A4	CYP3A4
P00742	FA10_HUMAN	Coagulation factor X	F10
P14635	CCNB1_HUMAN	G2/mitotic-specific cyclin-B1	CCNB1
P20813	CP2B6_HUMAN	Cytochrome P450 2B6	CYP2B6
P99999	CYC_HUMAN	Cytochrome c	CYCS
P02778	CXL10_HUMAN	C-X-C motif chemokine 10	CXCL10
P35368	ADA1B_HUMAN	Alpha-1B adrenergic receptor	ADRA1B
P55210	CASP7_HUMAN	Caspase-7	CASP7
P52789	HXK2_HUMAN	Hexokinase-2	HK2
P10145	IL8_HUMAN	Interleukin-8	CXCL8
P25963	IKBA_HUMAN	NF-kappa-B inhibitor alpha	NFKBIA
O15111	IKKA_HUMAN	Inhibitor of nuclear factor kappa-B kinase subunit alpha	CHUK
O14920	IKKB_HUMAN	Inhibitor of nuclear factor kappa-B kinase subunit beta	IKBKB
P08235	MCR_HUMAN	Mineralocorticoid receptor	NR3C2
P16581	LYAM2_HUMAN	E-selectin	SELE
P08034	CXB1_HUMAN	Gap junction beta-1 protein	GJB1
P49327	FAS_HUMAN	Fatty acid synthase	FASN
P01148	GON1_HUMAN	Progonadoliberin-1	GNRH1
Q13873	BMPR2_HUMAN	Bone morphogenetic protein receptor type-2	BMPR2
P08173	ACM4_HUMAN	Muscarinic acetylcholine receptor M4	CHRM4
P27487	DPP4_HUMAN	Dipeptidyl peptidase 4	DPP4

Q9NRD8	DUOX2_HUMAN	Dual oxidase 2	DUOX2
P20309	ACM3_HUMAN	Muscarinic acetylcholine receptor M3	CHRM3
Q15822	ACHA2_HUMAN	Neuronal acetylcholine receptor subunit alpha-2	CHRNA2
P11229	ACM1_HUMAN	Muscarinic acetylcholine receptor M1	CHRM1
P25100	ADA1D_HUMAN	Alpha-1D adrenergic receptor	ADRA1D
Q03135	CAV1_HUMAN	Caveolin-1	CAV1
P35222	CTNB1_HUMAN	Catenin beta-1	CTNNB1
P27338	AOFB_HUMAN	Amine oxidase [flavin-containing] B	MAOB
P08588	ADRB1_HUMAN	Beta-1 adrenergic receptor	ADRB1
O15392	BIRC5_HUMAN	Baculoviral IAP repeat-containing protein 5	BIRC5
O14757	CHK1_HUMAN	Serine/threonine-protein kinase Chk1	CHEK1
P38936	CDN1A_HUMAN	Cyclin-dependent kinase inhibitor 1	CDKN1A
P35348	ADA1A_HUMAN	Alpha-1A adrenergic receptor	ADRA1A
Q16678	CP1B1_HUMAN	Cytochrome P450 1B1	CYP1B1
P08913	ADA2A_HUMAN	Alpha-2A adrenergic receptor	ADRA2A
P56817	BACE1_HUMAN	Beta-secretase 1	BACE1
P06850	CRF_HUMAN	Corticoliberin	CRH
P17302	CXA1_HUMAN	Gap junction alpha-1 protein	GJA1
P11926	DCOR_HUMAN	Ornithine decarboxylase	ODC1
P31749	AKT1_HUMAN	RAC-alpha serine/threonine-protein kinase	AKT1
P54289	CA2D1_HUMAN	Voltage-dependent calcium channel subunit alpha-2/delta-1	CACNA2 D1
P36544	ACHA7_HUMAN	Neuronal acetylcholine receptor subunit alpha-7	CHRNA7
P22303	ACES_HUMAN	Acetylcholinesterase	ACHE
P11712	CP2C9_HUMAN	Cytochrome P450 2C9	CYP2C9
Q96JK2	DCAF5_HUMAN	DDB1- and CUL4-associated factor 5	DCAF5
P21397	AOFA_HUMAN	Amine oxidase [flavin-containing] A	MAOA
P00918	CAH2_HUMAN	Carbonic anhydrase 2	CA2
Q13085	ACACA_HUMAN	Acetyl-CoA carboxylase 1	ACACA
P08172	ACM2_HUMAN	Muscarinic acetylcholine receptor M2	CHRM2
O14493	CLD4_HUMAN	Claudin-4	CLDN4
Q07812	BAX_HUMAN	Apoptosis regulator BAX	BAX

O96017	CHK2_HUMAN	Serine/threonine-protein kinase Chk2	CHEK2
P41594	GRM5_HUMAN	Metabotropic glutamate receptor 5	GRM5
P06213	INSR_HUMAN	Insulin receptor	INSR
O75840	KLF7_HUMAN	Krueppel-like factor 7	KLF7
P04626	ERBB2_HUMAN	Receptor tyrosine-protein kinase erbB-2	ERBB2
P08709	FA7_HUMAN	Coagulation factor VII	F7
P15407	FOSL1_HUMAN	Fos-related antigen 1	FOSL1
P60568	IL2_HUMAN	Interleukin-2	IL2
P61925	IPKA_HUMAN	cAMP-dependent protein kinase inhibitor alpha	PKIA
P03372	ESR1_HUMAN	Estrogen receptor	ESR1
P56537	IF6_HUMAN	Eukaryotic translation initiation factor 6	EIF6
P19419	ELK1_HUMAN	ETS domain-containing protein Elk-1	ELK1
Q08462	ADCY2_HUMAN	Adenylate cyclase type 2	ADCY2
P20248	CCNA2_HUMAN	Cyclin-A2	CCNA2
P01100	FOS_HUMAN	Proto-oncogene c-Fos	FOS
P21860	ERBB3_HUMAN	Receptor tyrosine-protein kinase erbB-3	ERBB3
P42330	AK1C3_HUMAN	Aldo-keto reductase family 1 member C3	AKR1C3
P35869	AHR_HUMAN	Aryl hydrocarbon receptor	AHR
P05090	APOD_HUMAN	Apolipoprotein D	APOD
Q14790	CASP8_HUMAN	Caspase-8	CASP8
P00533	EGFR_HUMAN	Epidermal growth factor receptor	EGFR
Q92731	ESR2_HUMAN	Estrogen receptor beta	ESR2
P01583	IL1A_HUMAN	Interleukin-1 alpha	IL1A
P03956	MMP1_HUMAN	Interstitial collagenase	MMP1
P17538	CTRB1_HUMAN	Chymotrypsinogen B	CTRB1
Q16539	MK14_HUMAN	Mitogen-activated protein kinase 14	MAPK14
P42262	GRIA2_HUMAN	Glutamate receptor 2	GRIA2
P45983	MK08_HUMAN	Mitogen-activated protein kinase 8	MAPK8
P17252	KPCA_HUMAN	Protein kinase C alpha type	PRKCA
Q9GZT9	EGLN1_HUMAN	Egl nine homolog 1	EGLN1
P02751	FINC_HUMAN	Fibronectin	FN1
P30968	GNRHR_HUMAN	Gonadotropin-releasing hormone receptor	GNRHR
Q02156	KPCE_HUMAN	Protein kinase C epsilon type	PRKCE

Q12809	KCNH2_HUMAN	Potassium voltage-gated channel subfamily H member 2	KCNH2
P01579	IFNG_HUMAN	Interferon gamma	IFNG
P17936	IBP3_HUMAN	Insulin-like growth factor-binding protein 3	IGFBP3
P10914	IRF1_HUMAN	Interferon regulatory factor 1	IRF1
P09601	HMOX1_HUMAN	Heme oxygenase 1	HMOX1
P05112	IL4_HUMAN	Interleukin-4	IL4
P05362	ICAM1_HUMAN	Intercellular adhesion molecule 1	ICAM1
P22301	IL10_HUMAN	Interleukin-10	IL10
P08253	MMP2_HUMAN	72 kDa type IV collagenase	MMP2
P14598	NCF1_HUMAN	Neutrophil cytosol factor 1	NCF1
Q08209	PP2BA_HUMAN	Serine/threonine-protein phosphatase 2B catalytic subunit alpha isoform	PPP3CA
P04792	HSPB1_HUMAN	Heat shock protein beta-1	HSPB1
P07900	HS90A_HUMAN	Heat shock protein HSP 90-alpha	HSP90AA1
Q15113	PCOC1_HUMAN	Procollagen C-endopeptidase enhancer 1	PCOLCE
P15408	FOSL2_HUMAN	Fos-related antigen 2	FOSL2
Q16236	NF2L2_HUMAN	Nuclear factor erythroid 2-related factor 2	NFE2L2
O95644	NFAC1_HUMAN	Nuclear factor of activated T-cells, cytoplasmic 1	NFATC1
Q9UBS5	GABR1_HUMAN	Gamma-aminobutyric acid type B receptor subunit 1	GABBR1
P08100	OPSD_HUMAN	Rhodopsin	RHO
P35372	OPRM_HUMAN	Mu-type opioid receptor	OPRM1
Q07820	MCL1_HUMAN	Induced myeloid leukemia cell differentiation protein Mcl-1	MCL1
Q00987	MDM2_HUMAN	E3 ubiquitin-protein ligase Mdm2	MDM2
P08581	MET_HUMAN	Hepatocyte growth factor receptor	MET
Q15596	NCOA2_HUMAN	Nuclear receptor coactivator 2	NCOA2
P42345	MTOR_HUMAN	Serine/threonine-protein kinase mTOR	MTOR
Q9BZD4	NUF2_HUMAN	Kinetochore protein Nuf2	NUF2
P09960	LKHA4_HUMAN	Leukotriene A-4 hydrolase	LTA4H
Q8NHU6	TDRD7_HUMAN	Tudor domain-containing protein 7	TDRD7
P49767	VEGFC_HUMAN	Vascular endothelial growth factor C	VEGFC
P19875	CXCL2_HUMAN	C-X-C motif chemokine 2	CXCL2

P14672	GLUT4_HUMAN	Solute carrier family 2, facilitated glucose transporter member 4	SLC2A4
Q16665	HIF1A_HUMAN	Hypoxia-inducible factor 1-alpha	HIF1A
P48023	TNFL6_HUMAN	Tumor necrosis factor ligand superfamily member 6	FASLG
Q9NUW8	TYDP1_HUMAN	Tyrosyl-DNA phosphodiesterase 1	TDP1
P00734	THRB_HUMAN	Prothrombin	F2
P14679	TYRO_HUMAN	Tyrosinase	TYR
P07477	TRY1_HUMAN	Trypsin-1	PRSS1
P35354	PGH2_HUMAN	Prostaglandin G/H synthase 2	PTGS2
P10451	OSTP_HUMAN	Osteopontin	SPP1
P37231	PPARG_HUMAN	Peroxisome proliferator-activated receptor gamma	PPARG
Q03181	PPARD_HUMAN	Peroxisome proliferator-activated receptor delta	PPARD
P07204	TRBM_HUMAN	Thrombomodulin	THBD
P00749	UROK_HUMAN	Urokinase-type plasminogen activator	PLAU
P35968	VGFR2_HUMAN	Vascular endothelial growth factor receptor 2	KDR
P07550	ADRB2_HUMAN	Beta-2 adrenergic receptor	ADRB2
Q07817	B2CL1_HUMAN	Bcl-2-like protein 1	BCL2L1
Q14209	E2F2_HUMAN	Transcription factor E2F2	E2F2
P04150	GCR_HUMAN	Glucocorticoid receptor	NR3C1
