# Supplementary figures and images for: Anti-inflammatory effects of Chaishi Tuire Granules on influenza A treatment by mediating TRAF6/MAPK14 axis
Source: Front Med (Lausanne). 2022 Nov 14;9:943681. doi: 10.3389/fmed.2022.943681 (PMC9701735; doi:10.3389/fmed.2022.943681)

Raw, uncropped Western blot image

Actin:

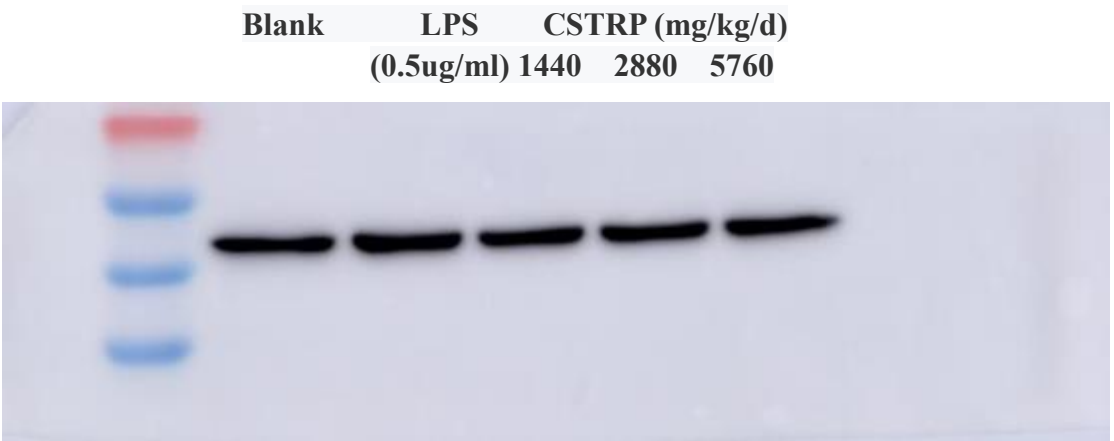

TRAF6:

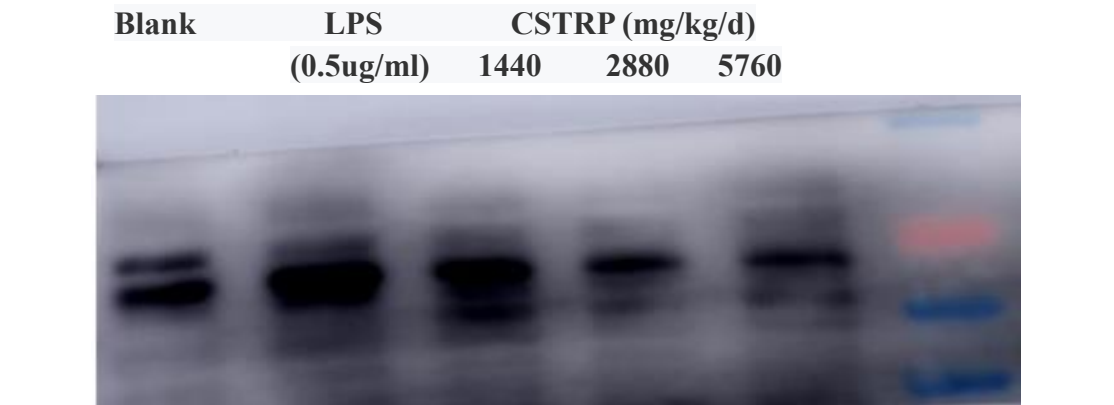

MAPK14:

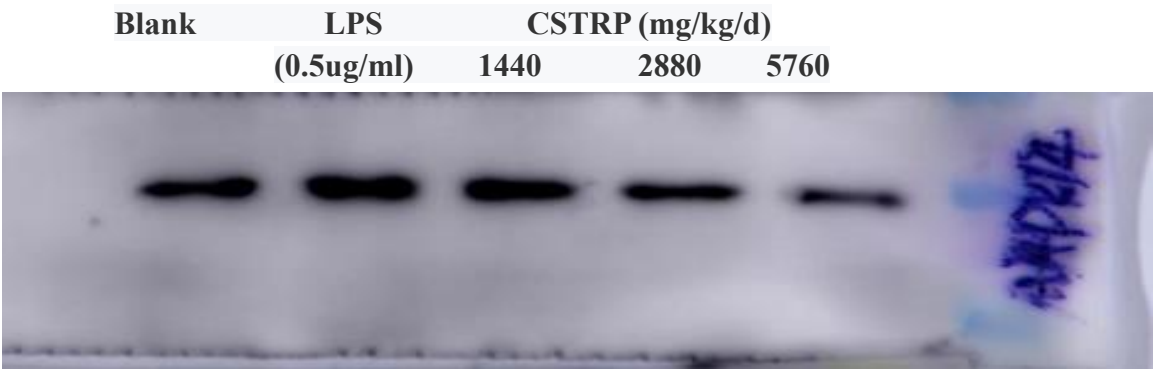

Supplement: Supplementary file 1 [file Data_Sheet_1.ZIP › Raw Data/Raw, uncropped Western blot image.pdf]

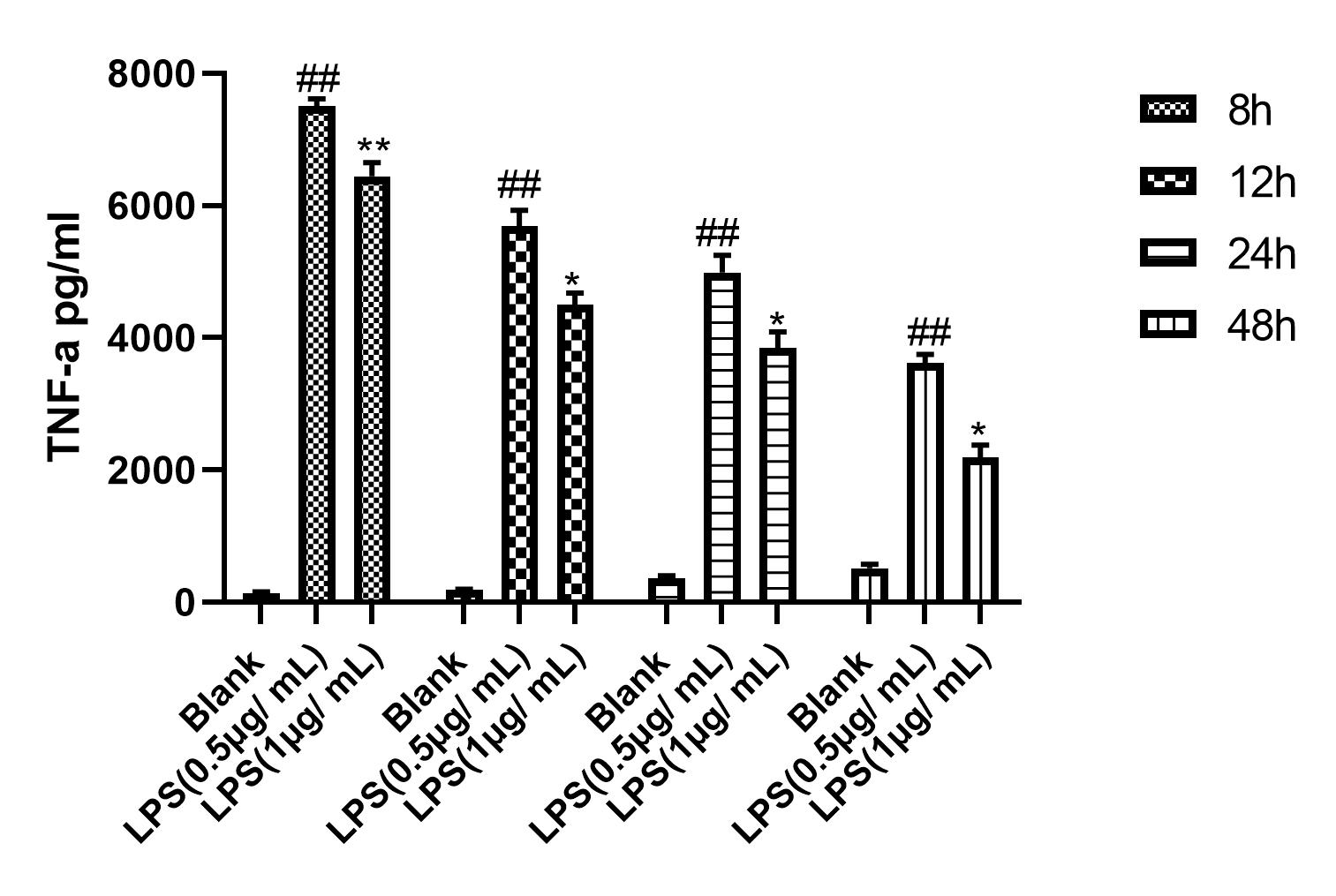

Supplement: Supplementary file 1 [file Data_Sheet_1.ZIP › Raw Data/Supplementary Material/Supplementary Fig. S2.jpg]

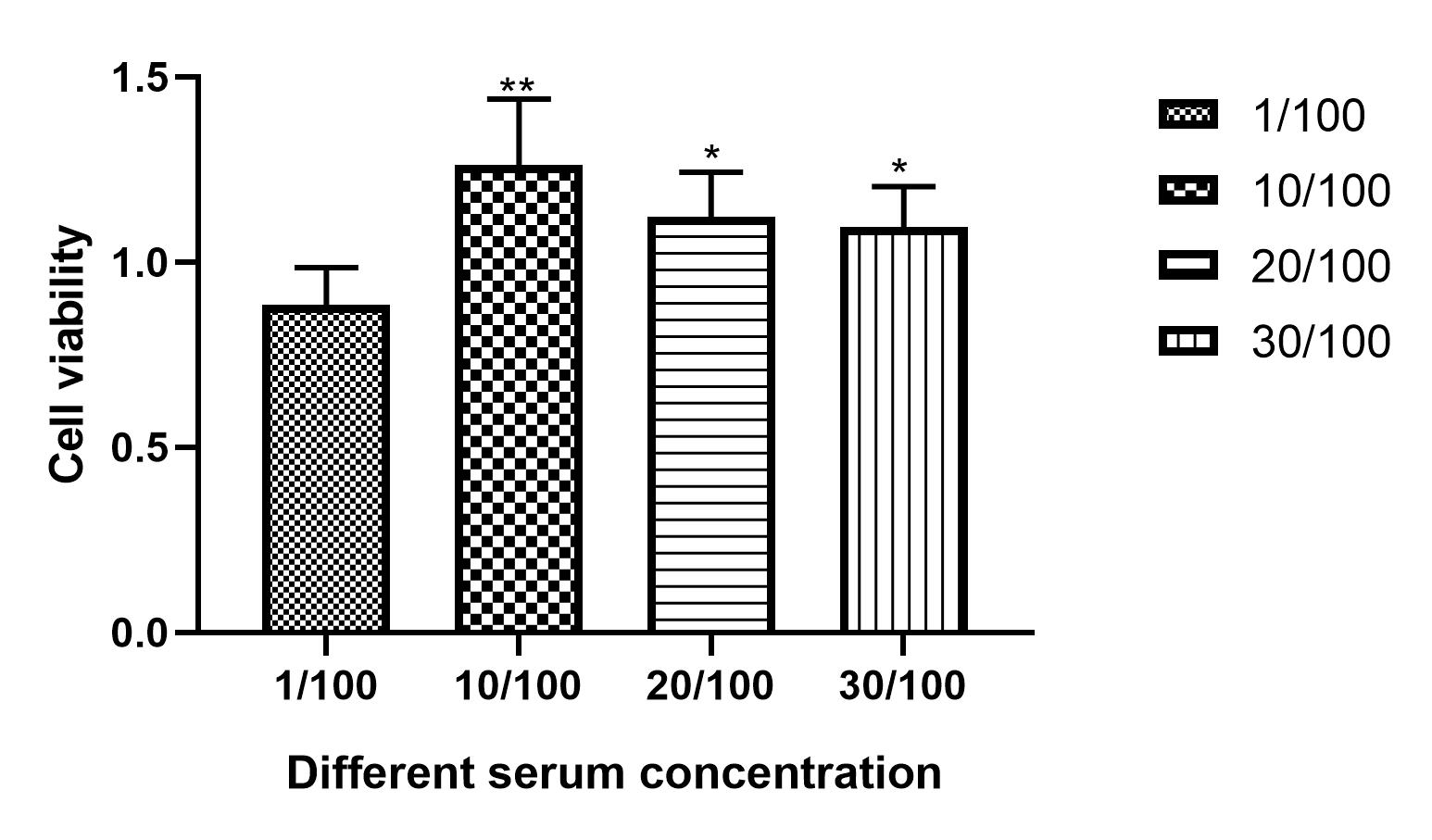

Supplement: Supplementary file 1 [file Data_Sheet_1.ZIP › Raw Data/Supplementary Material/Supplementary Fig.S1.jpg]
